# *ATAD3* gene cluster deletions cause cerebellar dysfunction associated with altered mitochondrial DNA and cholesterol metabolism

**DOI:** 10.1093/brain/awx094

**Published:** 2017-05-24

**Authors:** Radha Desai, Ann E. Frazier, Romina Durigon, Harshil Patel, Aleck W. Jones, Ilaria Dalla Rosa, Nicole J. Lake, Alison G. Compton, Hayley S. Mountford, Elena J. Tucker, Alice L. R. Mitchell, Deborah Jackson, Abdul Sesay, Miriam Di Re, Lambert P. van den Heuvel, Derek Burke, David Francis, Sebastian Lunke, George McGillivray, Simone Mandelstam, Fanny Mochel, Boris Keren, Claude Jardel, Anne M. Turner, P. Ian Andrews, Jan Smeitink, Johannes N. Spelbrink, Simon J. Heales, Masakazu Kohda, Akira Ohtake, Kei Murayama, Yasushi Okazaki, Anne Lombès, Ian J. Holt, David R. Thorburn, Antonella Spinazzola

**Affiliations:** 1 MRC Laboratory, Mill Hill, London NW71AA, UK; 2 Murdoch Childrens Research Institute, Royal Children’s Hospital and Department of Paediatrics, University of Melbourne, Melbourne VIC 3052, Australia; 3 Department of Clinical Neurosciences, Institute of Neurology, Royal Free Campus, University College London, NW3 2PF, UK; 4 Bioinformatics and Biostatistics, Francis Crick Institute, 1 Midland Road, London NW1 1AT, UK; 5 Mitochondrial Biology Unit, Hills Road, Cambridge, CB2 0XY, UK; 6 Radboud Center for Mitochondrial Medicine, Radboud University Medical Center, Nijmegen, The Netherlands; 7 Department of Genetics and Genomic Medicine, Institute of Child Health, University College London, London, UK and Laboratory Medicine, Great Ormond Street Hospital, London, UK; 8 Victorian Clinical Genetics Services, Murdoch Children’s Research Institute, Melbourne VIC 3052, Australia; 9 Department of Pathology, University of Melbourne, Melbourne 3052, Australia; 10 Royal Women’s Hospital, Melbourne, VIC 3052, Australia; 11 The Florey Institute of Neuroscience and Mental Health Melbourne, Australia; 12 Departments of Radiology and Paediatrics, University of Melbourne, Melbourne, Australia; 13 AP-HP, Department of Genetics, GHU Pitié-Salpêtrière, Paris, F-75651 France; 14 Inserm U975; CNRS UMR 7225, ICM; F-75013, Paris, France; 15 AP-HP, Service de Biochimie Métabolique et Centre de Génétique moléculaire et chromosomique, GHU Pitié-Salpêtrière, Paris, F-75651 France; 16 Inserm U1016; CNRS UMR 8104; Université Paris-Descartes-Paris 5; Institut Cochin, 75014 Paris, France; 17 Department of Clinical Genetics, Sydney Children’s Hospital, Sydney, NSW, Australia; 18 School of Women’s and Children’s Health, University of New South Wales, Kensington, NSW, Australia; 19 Department of Paediatric Neurology, Sydney Children’s Hospital, Sydney, NSW, Australia; 20 Department of Molecular Neuroscience, Institute of Neurology, University College London, Queen Square, London, UK; 21 Division of Translational Research, Research Center for Genomic Medicine, Saitama Medical University, Hidaka-shi, Saitama, Japan; 22 Department of Pediatrics, Saitama Medical University, Moroyama-machi, Iruma-gun, Saitama, Japan; 23 Department of Metabolism, Chiba Children’s Hospital, Chiba, Japan; 24 Division of Functional Genomics and Systems Medicine, Research Center for Genomic Medicine, Saitama Medical University, Hidaka-shi, Saitama, Japan; 25 Biodonostia Health Research Institute, 20014 San Sebastián, Spain. IKERBASQUE, Basque Foundation for Science, 48013 Bilbao, Spain; 26 MRC Centre for Neuromuscular Diseases, UCL Institute of Neurology and National Hospital for Neurology and Neurosurgery, Queen Square, London WC1N 3BG, UK

**Keywords:** mitochondrial DNA, mitochondrial disease, cerebellar hypoplasia, ATAD3, cholesterol

## Abstract

Although mitochondrial disorders are clinically heterogeneous, they frequently involve the central nervous system and are among the most common neurogenetic disorders. Identifying the causal genes has benefited enormously from advances in high-throughput sequencing technologies; however, once the defect is known, researchers face the challenge of deciphering the underlying disease mechanism. Here we characterize large biallelic deletions in the region encoding the *ATAD3C*, *ATAD3B* and *ATAD3A* genes. Although high homology complicates genomic analysis of the *ATAD3* defects, they can be identified by targeted analysis of standard single nucleotide polymorphism array and whole exome sequencing data. We report deletions that generate chimeric *ATAD3B*/*ATAD3A* fusion genes in individuals from four unrelated families with fatal congenital pontocerebellar hypoplasia, whereas a case with genomic rearrangements affecting the *ATAD3C*/*ATAD3B* genes on one allele and *ATAD3B*/*ATAD3A* genes on the other displays later-onset encephalopathy with cerebellar atrophy, ataxia and dystonia. Fibroblasts from affected individuals display mitochondrial DNA abnormalities, associated with multiple indicators of altered cholesterol metabolism. Moreover, drug-induced perturbations of cholesterol homeostasis cause mitochondrial DNA disorganization in control cells, while mitochondrial DNA aggregation in the genetic cholesterol trafficking disorder Niemann-Pick type C disease further corroborates the interdependence of mitochondrial DNA organization and cholesterol. These data demonstrate the integration of mitochondria in cellular cholesterol homeostasis, in which ATAD3 plays a critical role. The dual problem of perturbed cholesterol metabolism and mitochondrial dysfunction could be widespread in neurological and neurodegenerative diseases.

## Teaser

Mitochondrial DNA dysfunction causes a range of neurological diseases. Desai, Frazier *et al*. show that deletions in the *ATAD3* gene cluster create chimeric proteins that are associated with cerebellar defects, mitochondrial DNA disorganisation and perturbed cholesterol homeostasis. The findings link mitochondrial DNA, cholesterol, and brain development and function.

## Introduction

Mutations in mtDNA and in the nuclear-encoded factors required for mtDNA maintenance and expression result in a broad range of human diseases, most of which affect the CNS (Area-Gomez and Schon, 2014). Originally believed to float free in the mitochondrial matrix unfettered by packaging proteins, it is now established that mtDNA is organized in nucleoprotein complexes, called nucleoids, which are associated with the inner mitochondrial membrane (Spelbrink, 2010). The apparatus mediating the interaction with the inner mitochondrial membrane is predicted to have a role in mtDNA organization and distribution, and its dysfunction might result in alteration of nucleoid structure and composition, which in turn could adversely affect mtDNA segregation and membrane architecture, leading to disease. Cholesterol co-sediments with the mtDNA (Gerhold *et al.*, 2015) and if the association is physiologically meaningful, perturbed cholesterol homeostasis should result in mtDNA abnormalities, as should defects in factors effecting cholesterol–mtDNA interactions. A candidate to participate in such interactions is ATAD3, ATPase family, AAA+ domain containing 3, which was assigned as a detergent-resistant component of mitochondrial nucleoids with potential roles in mtDNA organization and segregation (He *et al.*, 2007) and enhancing hormonal-induced steroidogenesis (Issop *et al.*, 2015). Moreover, ATAD3 co-purifies with SPTLC1 and SPTLC2 (serine palmitoyltransferase, long chain base subunits 1 and 2) (He *et al.*, 2012) that synthesize sphingolipids, which form membrane micro-domains with cholesterol, and a small fraction of ATAD3 forms an 800 kDa complex that co-migrates with a labelled cholesterol probe (Rone *et al.*, 2012). Hence, ATAD3 may have overlapping roles in cholesterol distribution in mitochondria and mtDNA organization. ATAD3 has also been linked to adipogenesis and lipid metabolism (Hoffmann *et al.*, 2009), mitochondrial translation (He *et al.*, 2012) and iron and heme homeostasis (van den Ecker *et al.*, 2015).

Most species have a single *ATAD3* gene but hominids have a cluster of three genes arranged in tandem close to the telomere of chromosome 1p: *ATAD3C*, *ATAD3B* and *ATAD3A.* A recurrent *de novo* dominant missense mutation in *ATAD3A* was recently shown to cause a phenotype comprising global developmental delay, hypotonia, optic atrophy, axonal neuropathy, and hypertrophic cardiomyopathy in five unrelated subjects (Harel *et al.*, 2016). That study also identified a homozygous *ATAD3A* missense mutation in siblings with congenital cataract, ataxia and seizures plus biallelic deletions of *ATAD3A* and adjacent *ATAD3* genes in one subject with severe cerebellar hypoplasia and neonatal death. A dominant mutation in *ATAD3A* was later described to cause hereditary spastic paraplegia and axonal neuropathy (Cooper *et al.*, 2017). Here, we report six subjects with cerebellar pathology, all associated with biallelic genomic rearrangements affecting the *ATAD3* gene cluster. In four of five families, the affected individuals had fatal congenital pontocerebellar hypoplasia with a simplified gyral pattern, and the single adult case had cerebellar atrophy with dystonia and ataxia. At the cellular level, we demonstrated that ATAD3 deficiency causes aberrant mtDNA organization and is associated with elevated free cholesterol and increased expression of genes involved in cholesterol metabolism. We also show that genetic or pharmacological perturbations of cellular cholesterol homeostasis perturb mtDNA organization. The consequences of the *ATAD3* deletions for mtDNA organization and cholesterol metabolism offer a pathogenetic explanation for the disorder.

## Materials and methods

### Determination of deletion and breakpoints

Molecular karyotyping of DNA was performed with the Illumina HumanCytoSNP-12 (version 2.1) or Infinium CoreExome-24 arrays, as previously described (Bruno *et al.*, 2011). Automated detection of long contiguous segments of homozygosity was performed with the CNVPartition v3.1.6 algorithm in KaryoStudio software. SNP genotypes were generated in GenomeStudio software (Illumina) with data from a set of 102 intra-run samples.

A custom comparative genomic hybridization (CGH) NimbleGen 12 × 135 K array (Roche Diagnostics) was designed to densely tile 1034 MitoExome genes encoding known mitochondrial proteins (Calvo *et al.*, 2012). Targeted exonic regions were tiled to an average probe spacing of 50 bp, intronic regions to an average of 900 bp, and regions directly upstream and downstream of targeted genes were covered forming a low-resolution backbone tiled at 3600 bp. CGH arrays were performed, scanned and analysed in accordance with manufacturer’s recommendations using gender matched, pooled DNA from seven unaffected individuals as a control.

Intragenic deletions were further investigated by long-range PCR amplification and sequencing of the junction region (BigDye® v3.1 terminators; Applied Biosystems) to better define the breakpoints. The primer sequences used in this study and their binding sites within the *ATAD3* region are shown in Supplementary Fig. 1.

### Gene-specific RNA studies

RNA was extracted from cultured fibroblasts using the Illustra RNAspin Mini Kit (GE healthcare) and cDNA was generated using the SuperScript® III First strand synthesis system (Invitrogen) as per manufacturers’ protocols. For analysis of nonsense-mediated decay and mRNA splicing, fibroblasts were cultured in medium with and without 100 ng/μl cycloheximide for 24 h before RNA preparation (Lamande *et al.*, 1998). Quantitative reverse transcription (qRT)-PCR analysis of fibroblast *ATAD3A* and *ATAD3B* expression was performed as previously described for other genes (Tucker *et al.*, 2013), with the following modifications; each 20 μl PCR reaction contained 2.5 μl of cDNA synthesized from 800 ng of mRNA, 10 μl of SensiFAST^™^ SYBR® Green (Bioline) and 0.5 μM each of forward and reverse primers, and was measured in triplicate on each plate. Nucleotide variation between *ATAD3A* and *ATAD3B* transcripts and primers used are highlighted in Supplementary Fig. 1B. Results were normalized to *HPRT* expression (primers 5’-CCTGGCGTCGTGATTAGTGA and 5’-CGAGCAAGACGTTCAGTCCT) and Sanger sequencing of amplicons confirmed specificity.

### RNA-Seq analysis

For each sample, total RNA was extracted from ∼8 × 10^6^ fibroblasts using TRIzol® reagent (Sigma), quantified by NanoDrop and quality checked using the Agilent Bioanalyzer. Samples that showed minimal degradation as measured by RNA integrity number (RIN) > 8.0 were further processed for Illumina sequencing library preparation with the TruSeq Stranded mRNA HT Sample Prep Kit (Illumina, Part# RS-122-2103). Libraries were generated from 1 μg of total RNA and sequenced on the Illumina HiSeq 4000, using the paired-end 101 bp dual indexing protocol. FastQ files were generated using CASAVA BCL to FastQ (version 2.16). Sequencing yield was typically ∼70 million strand-specific paired-end reads. The RSEM package (version 1.2.29) (Li and Dewey, 2011) in conjunction with the STAR alignment algorithm (version 2.5.1b) (Dobin *et al.*, 2013) was used for the mapping and subsequent gene-level counting of the sequenced reads with respect to hg19 Ensembl genes downloaded from the UCSC Table Browser (Karolchik *et al.*, 2004) on 14 April 2016. The ‘–forward-prob’ parameter was set to ‘0’ and all other parameters were kept as default. Differential expression analysis was performed with the DESeq2 package (version 1.10.1) (Love *et al.*, 2014) within the R programming environment (version 3.2.3) (R Core Team, 2015). An adjusted *P*-value of ≤ 0.05 was used as the significance threshold for the identification of differentially expressed genes. All raw RNA-Seq sequence data and per sample transcript per million counts generated by RSEM can be accessed via GEO (GEO ID GSE86550).

### Pathway analysis

Gene set enrichment analysis for differentially expressed genes was performed by Gene Ontology Pathway and Biological processes using GeneGo MetaCore (https://portal.genego.com/).

### Gene set enrichment analysis

Genes from each given pairwise comparison were ranked using the Wald statistic. Gene set enrichment analysis (GSEA; Subramanian *et al.*, 2005) pre-ranked analysis was performed with respect to MSigDB (version 5.1) C2 canonical pathways and C5 GO biological process. All parameters were kept as default except for enrichment statistic (classic), min size (5) and max size (50 000). Gene signatures with a false discovery rate (FDR) q-value of ≤ 0.05 were considered to be significant.

### Cell culture, DNA and enzyme analysis

Primary fibroblasts were cultured in Dulbecco’s modified Eagle’s medium (DMEM, Life Technologies) supplemented with 10% foetal bovine serum (Hyclone), 1% penicillin and streptomycin (PS, Life Technologies) at 37°C in a 5% CO_2_ atmosphere. All cells were negative for mycoplasma based on regular screening using LookOut® Mycoplasma PCR Detection Kit (Sigma). Total DNA was isolated from cultured human fibroblasts using DNeasy® Blood and Tissue Kit (QIAGEN), or from fibroblasts or blood using a NucleoBond® CB20 DNA Extraction kit (Scientifix), according to the manufacturer’s protocol. Estimation of mtDNA copy number was performed by quantitative PCR, as described previously for tissue biopsies (Pagnamenta *et al.*, 2006) and cultured fibroblasts (Dalla Rosa *et al.*, 2016).

Spectrophotometric enzyme assays assessing mitochondrial OXPHOS enzyme activities were performed in cultured fibroblast mitochondria and skeletal muscle or liver biopsy post-nuclear supernatants from Subjects S1a, S1b and S3 as described previously (Frazier and Thorburn, 2012). OXPHOS enzymes in skeletal muscle post-nuclear supernatants from Subject S5 were assayed as described elsewhere (Medja *et al.*, 2009).

### Immunoblotting

Protein fractionation, transfer and immuno-detection were performed as described (Dalla Rosa *et al.*, 2014), with some modifications. Muscle and liver samples were prepared as previously described (Cooper *et al.*, 2003). Cells were lysed on ice in phosphate-buffered saline (PBS), 0.1% n-dodecyl β-d-maltoside (DDM), 1% SDS, 1 × protease inhibitor cocktail (Roche), 50 U Benzonase® and phosphatase inhibitor complexes (Cell signalling), or in RIPA buffer containing 1× protease inhibitor cocktail (Roche). Protein concentration was measured by Lowry assay (DC^™^ Reagent, Bio-Rad) or BCA assay, and 10 μg of lysate analysed per lane. Primary antibodies were: mouse anti-GAPDH (1:20 000, Abcam), mouse anti-NDUFB8 (1:1000, Abcam), mouse anti-COX II (1:2000, Abcam), mouse anti-VDAC1 (Porin) (1:10000, Merck), rabbit anti-ATAD3 (1:60 000, gift from John Walker), rabbit anti-SREBF2 (1:1000, Abcam), rabbit anti-CES-1 (1:2000, Proteintech).

### Immunocytochemistry and cell imaging

Fibroblast cultures were incubated with 20 μM BrdU for 8 h and fixed with 2% paraformaldehyde for 15 min at room temperature, then treated with PBS containing 0.2% Triton^™^ X-100. After a 5 min PBS wash, cells were incubated for 90 min at 40°C in 2N HCl. Cells were blocked with 5% goat serum in PBS for 1 h, then incubated with primary antibody in PBS at 4°C overnight and subsequently with secondary antibodies for 2 h at ambient temperature. A 90 min incubation at room temperature with Alexa Fluor® 488 conjugated streptavidin (Invitrogen) was followed with a final set of PBS washes. The coverslips were mounted on glass slides using Progold with DAPI. For staining that did not include BrdU the HCl antigen retrieval step was omitted. Primary antibodies: mouse anti-DNA Progen (1:200, AC-30-10); rat anti-BrdU Bio-Rad (1:200, MCA2060); rabbit anti-Tom20 (1:400 Santa Cruz). Secondary antibodies: Alexa Fluor® 488 goat anti-mouse (1:500); Alexa Fluor® 488 goat anti-rat (1:500); Alexa Fluor® 568 goat anti-rabbit (1:1000). Unesterified cholesterol in fibroblasts was stained with filipin, using a cholesterol assay kit (Abcam), detected by wide-field fluorescence microscopy and quantified using ImageJ.

### Mitochondrial DNA foci sizing and counting

Nucleoid size was analysed using 3D confocal images of human fibroblast cells stained with anti-DNA antibody. Images were acquired using parameters set for a control field of cells at the beginning of each microscopy session. Individual files were put through particle analysis using Fiji (ImageJ), with a fluorescence intensity threshold set manually, such that only signal above background was considered. This threshold was maintained for all images. Then the Fiji plugin, 3D particle count, was used to count the number of particles across the stack of optical sections. The plug-in counts each particle and defines its size, generating datasets based on ∼1500–5000 particles. The particle information was transferred to a spreadsheet and arranged in ascending order by size. The total number of particles were then binned into 20 size categories (bins) and presented as a histogram. No attempt was made to create parameter settings that would produce an accurate estimate of the total number of mtDNA foci (nucleoid number) in a cell.

## Results

### Clinical and biochemical features associated with deletions in the *ATAD3* gene cluster

**Figure 1:**
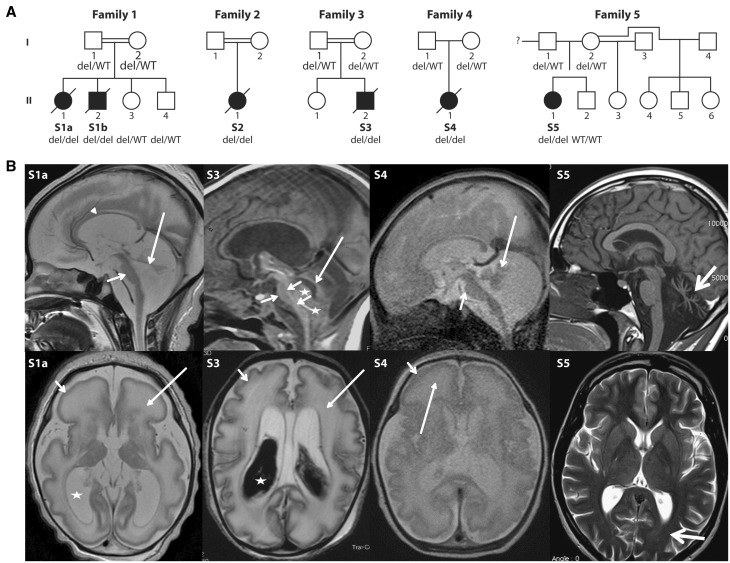
**Pedigrees and brain MRI from five unrelated families with cerebellar disorders.** (**A**) Pedigrees and *ATAD3* genotypes for available members of Families 1–5. (**B**) Brain MRI of Subjects S1a, S3, S4 and S5. *Top row*: Sagittal images of Subjects S1a, S3 and S4 in the neonatal period and Subject S5 at 22 years of age. The neonates have severe brainstem and cerebellar hypoplasia with flat pons (short arrows) and tiny cerebellar vermis (long arrows). There is increase of the tegmento-vermian angle and *ex vacuo* enlargement of the posterior fossa CSF spaces. Arrowheads indicate the thin corpus callosum. Stars in Subject S3 show isointense blood products within and below the fourth ventricle. Subject S5 presents with severe hypoplasia/atrophy of the cerebellar vermis (thick arrow) with *ex vacuo* enlargement of the fourth ventricle; brainstem and normal corpus callosum are normal. *Bottom row*: Axial T_2_-weighted images show simplified sulcation and gyration more marked frontally (short arrow) and diffuse white matter T_2_ signal abnormality (long arrow) in Subjects S1a and S4. Similar but less severe changes are seen in Subject S3 with shallow simplified sulcation. Both subjects had a thin cortical ribbon, decreased white matter volumes with marked T_2_ hyperintensity, *ex vacuo* ventriculomegaly (stars) and prominence of the extra-axial CSF spaces in keeping with brain atrophy. Hypointense material within the lateral ventricles of Subject S3 is haemorrhage. Subject S5 has normal ventricles and subtle ‘frosted glass’ aspect of the posterior periventricular white matter (thick arrow). del = *ATAD3* deletion; WT = wild-type.

Six subjects from five unrelated families were included in the study (Fig. 1A), with four from consanguineous families. Informed consent for diagnostic and research studies was obtained for all subjects in accordance with the Declaration of Helsinki protocols and approved by local institutional review boards. Clinical features are summarized in Table 1, with brain imaging shown in Fig. 1B. Five of six subjects had an antenatal disease, associating lack of foetal movements or polyhydramnios, dysmorphic features and severe encephalopathy with marked pontocerebellar hypoplasia with a simplified gyral pattern. Subject S5 had a milder presentation characterized by mental retardation and dystonia in childhood, followed by onset of cerebellar ataxia and atrophy in adulthood. Detailed clinical descriptions are provided in the Supplementary material. Elevated blood or CSF lactate was noted in all the live-born neonatal subjects but not in Subject S5 (Table 1). Borderline deficiencies of OXPHOS enzymes were observed in tissues or cell lines from some patients, but these features were variable and did not lead to definitive diagnosis of mitochondrial disease (Table 1 and Supplementary Table 1; see also de Koning *et al.*, 1999).

**Table 1:** Clinical and laboratory findings in individuals with deletions in the *ATAD3* gene cluster

Subject ID	Sex Ethnicity Consanguinity	*ATAD3* deletion	Age at onset	Age at death	Symptoms	Brain imaging	Mitochondrial investigations
S1a	Female Iranian Consanguineous	ATAD3B/ATAD3A 38 054 bp deletion	38 weeks gestation	Day 5	Polyhydramnios, reduced foetal and no postnatal spontaneous movements, required ventilation, dysmorphic features, severe encephalopathy	Cerebellar and brainstem hypoplasia with simplified anterior gyral pattern anteriorly, diffuse white matter signal abnormality, marked *ex vacuo* ventricular dilatation consistent with global atrophy	Plasma lactate elevated up to 6.2 mM, borderline low CI, CIII, CIV in skeletal muscle but normal in skin fibroblasts. Decreased steady state levels of a critical CI and CIV subunit in fibroblasts and RNASeq results indicative of decreased expression of a broad range of OXPHOS factors.
S1b	Male Iranian Consanguineous	ATAD3B/ATAD3A 38 054 bp deletion	33 weeks gestation	Day 1	Reduced foetal movements, foetus died intrapartum during a lengthy labour	Not performed	Borderline-low CI, CIII, CIV in skeletal muscle and borderline- low CI, CII, CIII in liver.
S2	Female Dutch Consanguineous	ATAD3B/ATAD3A 38 667 bp deletion	33 weeks gestation	Day 5	Polyhydramnios and lack of foetal movements. Caesarean section at 33 weeks due to foetal distress. No spontaneous movements, required ventilation, dysmorphic features, severe encephalopathy	Cerebellar hypoplasia, abnormalities of cortical gyration and periventricular white matter abnormalities	Plasma lactate elevated (2.7 to 4.4 mM), elevated urine 3-methylglutaconate and 3-methylglutatarate. Decreased CI, CIII, CIV in skin fibroblasts but normal in skeletal muscle and liver.
S3	Male Indian Consanguineous	ATAD3B/ATAD3A 38 667 bp deletion	34 weeks gestation	Day 2	Polyhydramnios. Caesarean section at 33 weeks due to foetal distress. Required ventilation, dysmorphic features, severe encephalopathy	Pontocerebellar hypoplasia, supratentorial white matter and cortical abnormalities, ventricular dilatation, simplified gyral pattern	Plasma lactate levels slightly elevated at 2.6 mM.
S4	Male Japanese Non-consanguineous	ATAD3B/ATAD3A 38 054 and 38 667 bp deletions	37 weeks gestation	7 months and 10 days	Reduced foetal movements, spontaneous delivery, cyanosis, no spontaneous respiration with Apgar score of 6/6, required ventilation throughout his life. Progressive cardiac hypertrophy	Cerebral, cerebellar and brain stem atrophy, simplified gyral pattern in the frontal lobes, haemorrhage from bilateral lateral ventricles	CSF lactate elevated (3.4 mM). Low oxygen consumption rate in skin fibroblasts but activities of CI, CII, CII+III, CIII and CIV were normal.
S5	Female French Non-consanguineous	ATAD3C/ATAD3B deletion ATAD3B/ATAD3A genomic rearrangement	Childhood	Alive at 30 years	Moderate mental retardation since childhood, dystonia, psychiatric problems, cerebellar ataxia since about 25 years	Cerebellar atrophy	Normal metabolic investigations. Normal histological analysis of muscle biopsy. In fibroblasts, decreased steady state levels of a critical CI and CIV subunit, and RNASeq results indicative of decreased expression of a broad range of OXPHOS factors (albeit less pronounced than for Subject S1a).

### *ATAD3* gene cluster deletions

**Figure 2:**
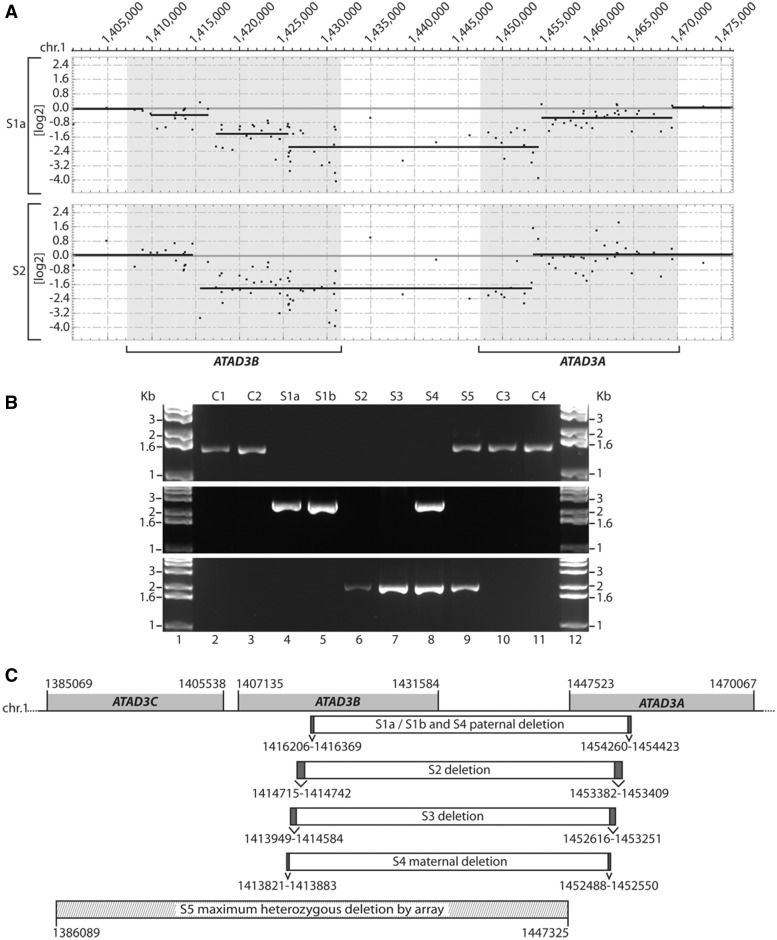
**Identification of genomic *ATAD3* deletions.** (**A**) A custom CGH array was used to delineate homozygous deletions detected on chromosome 1 p in DNA from Subjects S1a and S2. Shaded boxes indicate the location of the *ATAD3C*, *ATAD3B* and *ATAD3A* genes. Details of deleted regions predicted by SNP and CGH arrays are summarized in Supplementary Table 2. (**B**) Long-range PCRs were performed on genomic DNA from subjects and controls. Primers OT472 and OT473 (**middle panel**) flank the *ATAD3* deletion breakpoints predicted in Subject S1a by array CGH. Primers OT572 and OT575 (**bottom panel**) flank the *ATAD3* deletion breakpoints predicted in S2 by array CGH. As a control, primers OT570 and OT575 (**top panel**) were used since primer OT570 is located within the predicted deleted *ATAD3B/ATAD3A* region. (**C**) Genomic DNA sequencing of the breakpoint-spanning PCR products determined the *ATAD3B*/*ATAD3A* deletion boundaries in each subject, with chromosome 1 coordinates indicated (hg19). Ambiguous regions flanking the deletion boundaries that have identical sequence in *ATAD3B* and *ATAD3A* are identified by dark grey boxes. The deletion predicted by high-density SNP array for Subject S5 is indicated, with maximum and minimum deletion boundaries labelled by hatched boxes.

Deletions in the ATAD3 gene cluster were identified in affected individuals, using SNP and CGH arrays (Fig. 2A and Supplementary Table 2), and validated by long-range PCR (Fig. 2B and Supplementary Fig. 2). The five neonates with pontocerebellar hypoplasia all had biallelic deletions in the *ATAD3B*/*ATAD3A* region. In each case the exact breakpoints could not be precisely defined due to sequence identity within the breakpoint regions (i.e. substantial portions of the *ATAD3* genes constitute direct repeats) (Supplementary Fig. 2). Subjects S1a and S1b had a homozygous deletion of 38 054 bp while Subjects S2 and S3 both had homozygous deletions of 38 667 bp but with different breakpoints (Fig. 2A–C and Supplementary Fig. 2). Subject S4 was compound heterozygous for 38 054 bp and 38 667 bp deletions. Analysis of DNA from available unaffected members of each family indicated they each carried at least one wild-type *ATAD3* allele (Supplementary Fig. 3).

**Figure 3:**
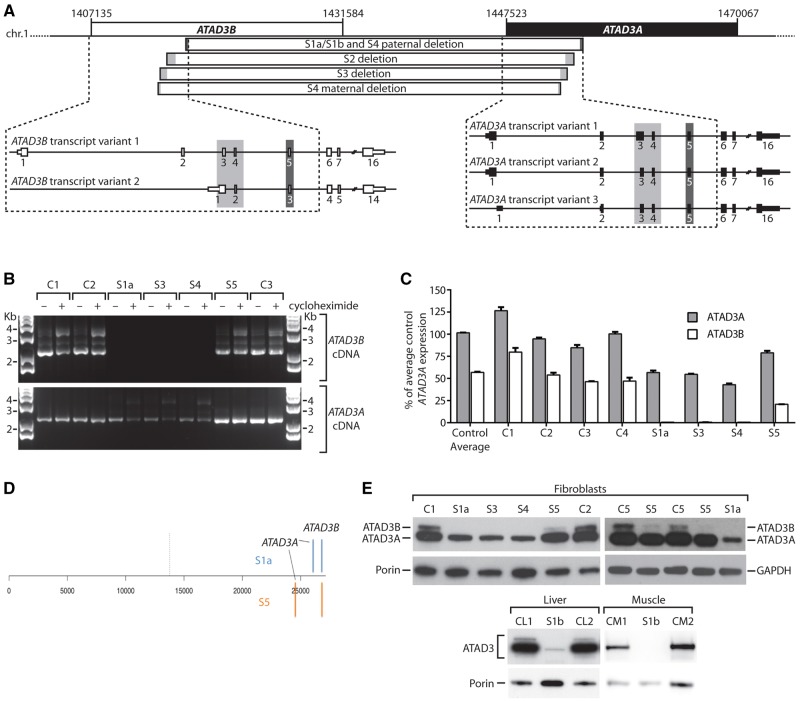
***ATAD3* gene cluster deletions result in decreased expression of *ATAD3A*, and the loss of *ATAD3B* at the mRNA and protein level.** (**A**) *ATAD3* deletions in relation to exon structure of full length *ATAD3B* and *ATAD3A* isoforms for Subjects S1a, S1b, S2 and S3. (**B**) Full length *ATAD3A* and *ATAD3B* isoforms were amplified from control and subject cDNA prepared from fibroblasts grown ± cycloheximide. Primers OT441 and OT443 were used to amplify *ATAD3A*, while primers OT441 and OT445 were used to amplify *ATAD3B.* The *ATAD3A* product amplified in S1a, S3 and S4 represents the fusion cDNA due to cross-hybridization of primer OT441. (**C**) The relative expression levels of *ATAD3A* versus *ATAD3B* were determined by qRT-PCR from four controls and Subjects S1a, S3, S4 and S5. Results were normalized to *HPRT* expression and presented as percent of average control *ATAD3A* expression; *n* = 3, error = SEM. Two-way ANOVA comparing all controls and subjects showed *ATAD3B* expression was significantly reduced in all subjects compared to individual controls (*P* < 0.0001), as was ATAD3A in S1a, S3 and S4 (*P* < 0.0001). For Subject S5, the reduction in ATAD3A was not significant in comparison to all controls. (**D**) Ranked gene expression list showing the positions of ATAD3A and ATAD3B in Subjects S1a and S5 versus control. The dotted line represents the position of genes that are expressed at the same level in subject and control samples; to the left, genes expressed more highly in the subjects; to the right, genes expressed less than the control. Total number of genes 26 689. S5 fibroblasts manifested reduced expression of *ATAD3B*, with log_2_ fold change (FC) = −1.31; and to a lesser extent the intact ATAD3A log_2_FC = −0.73. The transcripts of the *ATAD3B*/*ATAD3A* chimera of Subject S1a registered as decreased expression of both original genes; ATAD3B, log_2_FC−3.0, *ATAD3A*, log2FC = −1.50. (**E**) ATAD3 proteins detected by immunoblotting using a pan-specific antibody in tissues and fibroblasts from subjects, relative to controls. Porin or GAPDH was used as a loading control.

The deletions in Subjects S1 to S4 all predict the generation of a fusion gene between *ATAD3B* and *ATAD3A* (Fig. 2C and Supplementary Fig. 2A and C). S1a, S1b and paternal S4 deletions predict an mRNA fusion occurring near exon 5 while S2, S3 and S4 maternal deletions predict an mRNA fusion near exons 3 and 4 (Fig. 3A and Supplementary Fig. 4A–C). Hence, the expected transcripts would use the branch point and acceptor splice site for *ATAD3B* exon 5 in S1 and paternal S4 alleles, and the *ATAD3A* sites for the S2, S3 and maternal S4 alleles (*cf.*Supplementary Fig. 4B and 4C). Because of 100% homology of exons 3, 4 and 5 of *ATAD3B* and *ATAD3A*, the structure of the mRNA transcripts in all subjects was expected to be identical and cDNA amplification and sequencing was consistent with this interpretation (Fig. 3B and Supplementary Figs 4D and 5A). The predicted ATAD3B/ATAD3A fusion protein should be identical to ATAD3A isoform 2, apart from two missense variants (p.I7V and p.D73E, Supplementary Fig. 5C), and transcription under the *ATAD3B* promoter. qRT-PCR, using primers designed to distinguish between *ATAD3B* and *ATAD3A*, indicated that fibroblasts from Subjects S1a, S3 and S4 lacked any detectable *ATAD3B* mRNA and had decreased expression of *ATAD3A* (Fig. 3C).

High density SNP array analysis of Subject S5 indicated that one allele carried an *ATAD3C*/*ATAD3B* deletion of 43 to 61 kbp in size, while long-range PCR suggested the second allele had a similar *ATAD3B*/*ATAD3A* deletion to Subjects S2 and S3 (Fig. 2B and C). However, RNA analyses showed that the second allele is more complicated than a simple fusion gene. Unlike in Subjects S2 and S3, full length *ATAD3B* cDNA could be amplified from S5 fibroblasts but there was no cDNA sequence corresponding to the *ATAD3B*/*ATAD3A* fusion transcripts (Fig. 3B and Supplementary Fig. 4D). Sequencing of *ATAD3A* cDNA suggested one allele had at least one exon (exon 8) replaced by *ATAD3B* sequence, resulting in a minimum of two missense variants in the ATAD3A isoform 2 sequence (p.L269A and p.A271T, Supplementary Fig. 5C and E). Transcriptome analysis of Subjects S1a, S5 and control fibroblasts suggested the *ATAD3C* gene is not expressed in fibroblasts and indicated that *ATAD3A* and *ATAD3B* were among the most altered of the full set of expressed genes in both Subjects S1a and S5 (Fig. 3D); qRT-PCR confirmed that *ATAD3B*, and to a lesser extent *ATAD3A*, expression were decreased in Subject S5 (Fig. 3C). The precise genomic rearrangement on the second ATAD3 allele has not been defined but the simplest explanation that reconciles the DNA and RNA results is that both the *ATAD3A* and *ATAD3B* genes on the second allele have some sequence replaced by elements of the corresponding gene via interlocus gene conversion; as documented for other disease mutations, particularly between pairs of genes with high homology (Casola *et al.*, 2012; Dumont, 2015).

Immunoblotting of ATAD3 detected two species in control samples, as previously described (He *et al.*, 2007), the lower one being compatible with the 66 kDa ATAD3A isoform 2. Only one species of ∼65 kDa was detected in Subjects’ S1a, S3 and S4 samples, at much lower abundance than control cell lines. A more marked loss of the lower band was seen in liver and muscle of Subject S1b compared with fibroblasts (Fig. 3E). Thus, the protein data are fully concordant with the genomic mapping that predicted an ATAD3A/ATAD3B fusion protein indistinguishable in size from ATAD3A. Moreover, because *ATAD3A* is invariably more highly expressed than *ATAD3B* [see for example, He *et al.* (2007) and Fig. 3D], the decrease in expression can be attributed largely to the fusion gene being under the control of the *ATAD3B* promoter. In Subject S5, the band of the size expected for ATAD3B (72.6 kDa) was barely detectable (Fig. 3E), the residual signal can be explained by low expression of *ATAD3B*, or expression of the 71.4 kDa ATAD3A isoform 1. ATAD3A isoform 2 was less abundant than in controls, but greatly exceeded that of the fatal neonatal subjects (Fig. 3E), providing a ready explanation for the milder disease phenotype of Subject S5.

### Mitochondrial DNA abnormalities associated with *ATAD3* deletions

**Figure 4:**
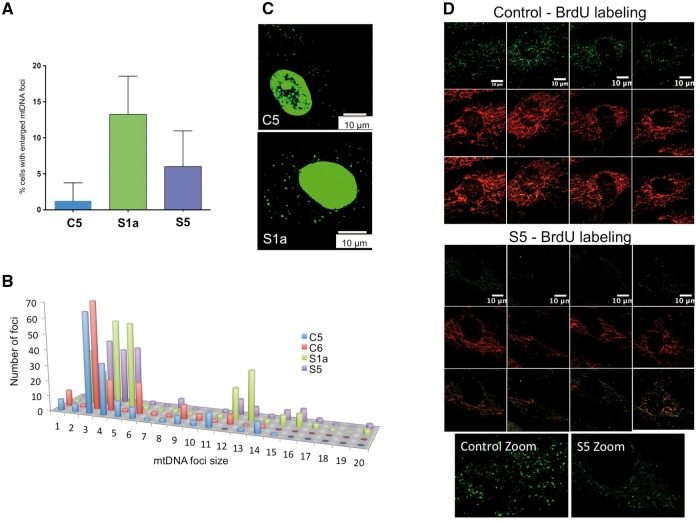
**ATAD3 deficiency is associated with mtDNA abnormalities.** The DNA of human fibroblasts was stained with anti-DNA antibody and the number of cells with enlarged mtDNAs was scored for cell lines C1, S1a and S5. (**A**) A minimum of 150 cells was counted for each cell line (*n* = 4 independent experiments, error bars are 1 standard deviation from the mean). See Supplementary Fig. 6 for representative images for Subject S5. (**B**) The signals of foci in the cytoplasm (mtDNA) plotted as frequency distribution after particle point analysis of a minimum of 20 cells. To smooth the data, signals were sorted into 20 ‘bins’ based on intensity. Foci falling in bins 1–6 all correspond to one mtDNA molecule based on detailed study of the images, including ones subjected to deconvolution analysis (Akman *et al.*, 2016), and the fact that most mtDNAs are organized as single copies (Kukat *et al.*, 2011), with the variation across the range expected to be the result of differences in condensation (packing) and efficiency of antibody access and coating. Many of the foci of bins 7–9 could be resolved to two or three separate or overlapping smaller dots indicating they contained two or three mtDNA molecules, whereas larger foci in bins 10 and above contained more than three copies of mtDNA. Very large mtDNA foci (bins > 11) were significantly more numerous in Subjects S1a (*P* < 0.0001) and S5 (*P* < 0.0001) fibroblasts than controls. (**C**) PicoGreen® staining of mtDNA reveals larger foci in cells of S1a than those of controls. Representative images showing the PicoGreen® stained structures in the cytoplasm (mtDNA). Nuclear DNA staining with PicoGreen® is highly variable (often it is undetectable) (He *et al.*, 2007) and so cannot serve as a reference. Microscope settings and image capture parameters were identical. PicoGreen® staining was as previously described (Ashley *et al.*, 2005). (**D**) BrdU incorporation into mtDNA detected by immunofluorescence. Single confocal optical sections of C1 and S5 fibroblasts treated with 20 μM BrdU for 8 h and with anti-BrdU antibody (green) and anti-ATAD3 antibody (red).

Mitochondrial DNA abnormalities are a major cause of mitochondrial dysfunction (Area-Gomez and Schon, 2014) and ATAD3A and ATAD3B co-purify with mtDNA (He *et al.*, 2007). Therefore, we determined the abundance of mtDNA in available tissues and cells; there were some differences in mtDNA copy number in Subjects S1a, S1b and S5 compared to controls (Supplementary Table 1), but not sufficient to regard as mtDNA depletion. Previously, modulating the expression of *ATAD3* in aneuploid cells produced only modest effects on mtDNA copy number but perturbed mtDNA organization (He *et al.*, 2007, 2012). Therefore, we analysed the size and number of mtDNA foci in fibroblasts from Subjects S1a and S5 (Supplementary Fig. 6), after anti-DNA labelling. The number of ATAD3-deficient cells with enlarged mtDNA foci was approximately three (Subject S5) to seven times (Subject S1a) greater than that of a representative control (Fig. 4A). Assessment of the frequency distribution of mtDNA foci sizes in the cells by particle point analysis showed that large mtDNA foci were significantly more numerous in Subject S1a and S5 fibroblasts than those of control cells (Fig. 4B), suggesting ATAD3 deficiency causes localized mtDNA aggregation (impaired distribution). Moreover, there was a substantial shift in signal from bin 3 (control) to bins 4 and 5 (Subjects S1a and S5), which is too small a change to represent an increase in the number of copies of mtDNA per foci, and more likely reflects altered packing or topology of individual mtDNA molecules. Consistent with this interpretation, PicoGreen® staining of the mtDNA (which is highly dependent on DNA topology; He *et al.*, 2007) showed that most foci of Subject S1a cells appeared larger than those of the control (Fig. 4C). ATAD3 deficiency was also associated with reduced labelling of mtDNA with BrdU in S5 fibroblasts, suggesting slow mtDNA synthesis (Fig. 4D).

### Pharmacological perturbation of cholesterol metabolism induces mtDNA clustering

**Figure 5:**
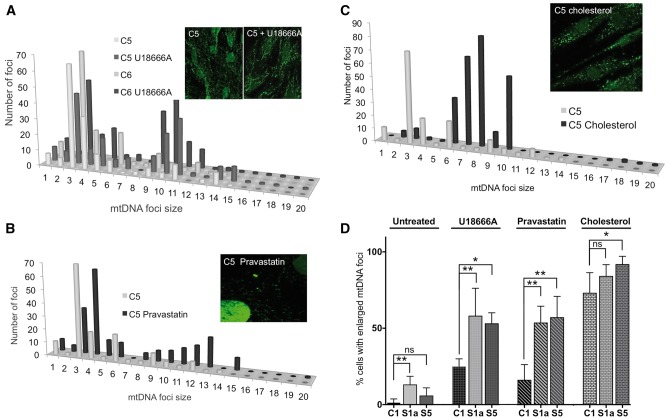
**U186666A, pravastatin and cholesterol increase the size of mitochondrial nucleoids in human fibroblasts.** The DNA of control human fibroblasts was stained with anti-DNA antibody after treating cells for 7 days without or with 5 μM U18666A (**A**), or pravastatin (**B**); or for 5 days with 5 mM cholesterol (**C**). *P*-values for the difference between large mtDNA foci (bins > 8) of treated versus untreated cells were *P* = 0.0116 (U18666A); *P* = 0.0011 (pravastatin); *P* = 0.0002 (cholesterol). The data are plotted as frequency distributions after particle point analysis. Insets are representative images. (**D**) The proportion of fibroblasts with enlarged mtDNA foci, after treatment with U18666A, pravastatin, or cholesterol, compared to untreated cells, based on a minimum count of 50 cells from each of three independent experiments, error bars are 1 standard deviation from the mean. Fibroblasts of Subjects S1a and S5 carry deletions in the *ATAD3* gene cluster. Data for the ‘untreated’ cells, derived from experiments carried out in parallel, are reproduced from Fig. 4A. Probability was determined using a one-way ANOVA test (uncorrected Fisher’s LSD). *P < 0.05; **P < 0.01, ***P < 0.001.

The effects of ATAD3 deficiency on mtDNA organization (Fig. 4A) could relate to its interactions with cholesterol (see ‘Introduction’ section). Therefore, we investigated whether disrupting cholesterol homeostasis exerted effects on the mtDNA similar to those resulting from ATAD3 deficiency. Control human fibroblasts were exposed to U18666A, an intracellular cholesterol transport inhibitor (Sparrow *et al.*, 1999); or the HMG-CoA reductase inhibitor pravastatin, one of a class of drugs used to reduce cholesterol synthesis in humans (Gould *et al.*, 1998), or soluble cholesterol. Treatment of human fibroblasts with 5 μM U18666A (Fig. 5A), or 5 μM pravastatin (Fig. 5B), for 7 days led to an increase in the number of large mtDNA foci. Cholesterol supplementation (5 mM for 5 days) produced an increase in the size of the majority of mtDNA foci, but individual nucleoids were not enlarged to the same extent as with U18666A or pravastatin treatment (Fig. 5C). Therefore, increases or decreases in cholesterol availability modify the organization of mtDNA in human cells. This suggests that strict maintenance of the cholesterol content of mitochondrial membranes in the vicinity of the mtDNA is necessary to support mtDNA segregation and distribution within the mitochondrial network.

U18666A or pravastatin treatment of ATAD3-deficient fibroblasts increased the number of cells with enlarged mtDNA foci, maintaining or amplifying the distinction from control cells (Fig. 5D). There were also marked effects on the size of individual foci, particularly in Subject S1a (Supplementary Fig. 7A and B). In contrast to these drugs, cholesterol supplementation reduced the mtDNA differences between ATAD3-deficient and control fibroblasts (Fig. 5D and Supplementary Fig. 7C).

### The cholesterol trafficking disorder, Niemann-Pick disease type C, is associated with mtDNA aggregation

**Figure 6:**
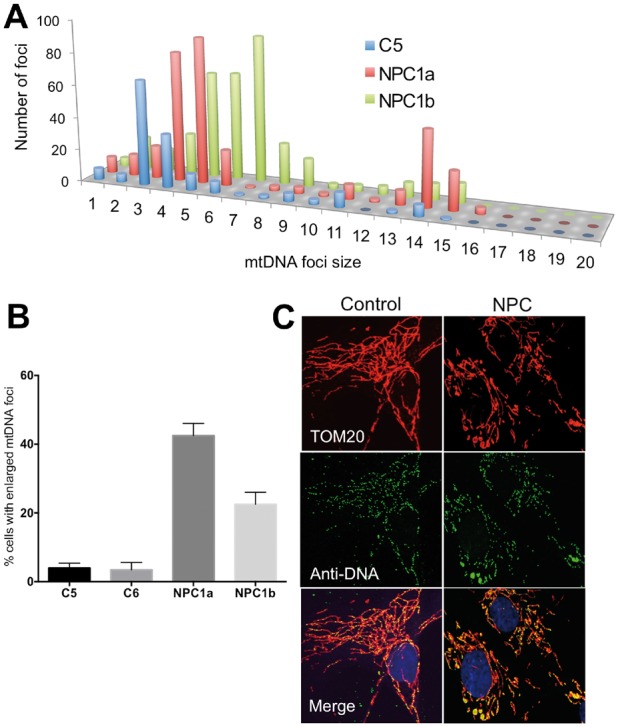
**Niemann-Pick type C disease is associated with mtDNA disorganization.** (**A**) Frequency distribution of mtDNA foci size in fibroblasts from a control and two individuals with NPC1 defects. (**B**) Proportion of cells with mtDNA clusters, error bars are 1 standard deviation from the mean. (**C**) Representative images. Anti-DNA (green) and anti-TOM20 labelling of fibroblasts. Yellow foci, and green foci bounded by red, are mtDNAs.

If changes in cholesterol and lipid metabolism were responsible for the mtDNA phenotypes displayed by ATAD3-deficient cells (Fig. 4), then mtDNA abnormalities should be evident in genetic disorders of cholesterol metabolism such as Niemann-Pick disease type C, caused by mutations in the intracellular cholesterol transporters NPC1 (MIM 257220) or NPC2 (MIM 607625) (Roff *et al.*, 1991). Recently, mitochondrial distension has been reported in cells of individuals with *NPC1* mutations (Schultz *et al.*, 2016). Such swellings (or bulbs) within the mitochondrial network have been associated with mtDNA clustering (Ban-Ishihara *et al.*, 2013; Dalla Rosa *et al.*, 2014). Analysis of the mitochondrial network in fibroblasts harbouring *NPC1* mutations showed morphological changes similar to those reported earlier (Schultz *et al.*, 2016). The distended mitochondria contained clusters of mtDNAs, and nucleoid size was greater than control cells (Fig. 6) implying that a genetic cholesterol trafficking defect perturbs mtDNA organization and distribution.

### ATAD3-deficient cells display perturbed cholesterol and lipid metabolism

**Figure 7:**
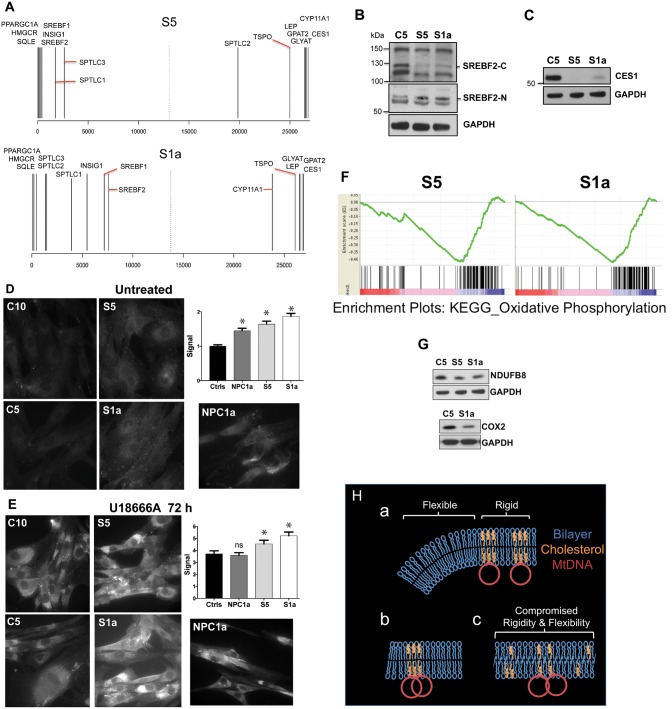
**Abnormal cholesterol homeostasis and reduced expression of OXPHOS factors associated with ATAD3 gene cluster deletions.** (**A**) Rank gene expression list showing the position of selected factors for Subjects S1a and S5 versus control (see also Supplementary Figs 8–10 and Supplementary material). (**B** and **C**) Proteins from fibroblasts of controls and S1a and S5 were fractionated by SDS-PAGE and probed with the indicated antibodies after blot transfer. There was no increase in the steady-state level of the larger isoforms of SREBF2 (located in the cytoplasm, SREBF2-C) among the subject samples but a SCAP processed SREBF2 isoform, which translocates to the nucleus (SREBF2-N) and resolves at ∼55 kDa, was increased in S1a and S5 samples (this particular species may be a phosphorylated form of SREBF2 (Krycer *et al.*, 2012); CES1 was greatly diminished. (**D** and **E**) Free cholesterol detected by filipin labelling of fibroblasts exposed to (**D**) no treatment, or (**E**) 5 μM U18666A for 72 h. *P < 0.05, ns = not significant. (**F**) Gene set enrichment plots for OXPHOS in Subjects S1a and S5. Each vertical black line represents an mRNA, with the most positively correlated to the left (in the red zone). Clustering at the negatively correlated end of the spectrum (blue zone) indicates the pathway is repressed compared to the reference. Consistent changes across a gene set give sharp curves (green lines) and S1a gave the sharper curve of the two. Moreover, for S1a OXPHOS was the third most negatively differentially expressed pathway or process, whereas it was 17th for S5. These observations are consistent with greater OXPHOS impairment in S1a than S5. (**G**) Immunoblots of respiratory chain components of complex IV (COX2) and complex I (NDUFB8). (**H**) Proposed arrangement of cholesterol in the mitochondrial inner membrane, the mtDNA in particular is not to scale. (**a**) In normal conditions localized high concentrations of cholesterol impart rigidity to the membrane for optimal organization and segregation of the mtDNA, whereas other regions require greater flexibility to form the highly invaginated membranes characteristic of the inner mitochondrial membrane. (**b**) If cholesterol is scarce there is insufficient sterol to permit mtDNA segregation. (**c**) Alternatively, if cholesterol is present in normal amounts but dispersed, both rigidity and flexibility are suboptimal; hence, a key role of ATAD3 may be to concentrate cholesterol where the mtDNA is located. Such membrane abnormalities (**b** or **c**) could cause the increase in mitochondrial turnover (mitophagy) reported for other ATAD3 mutants (Harel *et al.*, 2016).

Transcriptome analysis of Subjects S1a, S5 and control cells provided extensive evidence that the ATAD3 deletions perturb cholesterol and lipid metabolism. The most positively enriched pathway in Subject S5 cells was cholesterol biogenesis based on Meta-Core^TM^ software analysis (Supplementary Fig. 8), and the endogenous metabolic networks distinguishing Subject S5 cells most from control cells were linked to lipid metabolism (Supplementary Fig. 9A). Other factors of cholesterol metabolism significantly differentially expressed (FDR < 0.05 and a log_2_FC > 2) in Subject S5 were the endoplasmic reticulum membrane protein INSIG1, which plays a critical role in regulating cholesterol concentrations in the cell; and CYP11A1, which converts cholesterol to pregnenolone in the mitochondria, and has been linked previously to ATAD3 (Rone *et al.*, 2012) (Fig. 7A). SREBF2, the master regulator of cellular cholesterol synthesis (Horton *et al.*, 2002), was also significantly more highly expressed in cells from Subject S5 versus the control (Fig. 7A), and an isoform of SREBF2 was more abundant in Subjects’ S5 and S1a cells at the protein level (Fig. 7B). Although altered cholesterol metabolism was not as marked in Subject S1a’s samples, lipid metabolism again dominated the endogenous metabolic network analysis (Supplementary Fig. 8B), and key rate-limiting enzymes for cholesterol biosynthesis were among the most positively correlated genes of Subject S1a’s cells (Fig. 7A). Substantial and significant changes to two other factors involved in cholesterol homeostasis, carboxylesterase 1 (CES1) (Quiroga *et al.*, 2012; Ross *et al.*, 2014) and leptin (Hongo *et al.*, 2009) were also evident in S1a and S5 cells (Fig. 7A), with the former confirmed at the protein level (Fig. 7C). Moreover, unesterified (free) cholesterol was significantly increased in S1a and S5 fibroblasts (Fig. 7D) and exceeded that of a case of NPC as well as controls, after 72 h exposure to U18666A (Fig. 7E).

GSEA analysis (Subramanian *et al.*, 2005) corroborated the Meta-Core results, highlighting lipid metabolism and cholesterol and sterol biosynthesis as altered in ATAD3-deficient cells, especially those of Subject S5 (Supplementary Fig. 10). In addition, GSEA revealed that highly significant decreases in the OXPHOS system in the ATAD3 deletion cells (FDR q-value < 1 × 10^−16^ for Subjects S1a and S5) correlated with disease severity (Fig. 7F). Individual OXPHOS components tested were slightly decreased at the protein level (Fig. 7G), and thus were concordant with the GSEA.

## Discussion

Defects in mitochondrial function have been linked to pontocerebellar hypoplasia previously, particularly mutations in the *RARS2* tRNA synthetase gene (Edvardson *et al.*, 2007) and occasional cases with a mtDNA deletion (Biancheri *et al.*, 2011) or OXPHOS enzyme defect (as reported for Subject S2; de Koning *et al.*, 1999). We describe four unrelated families in whom chimeric *ATAD3B*/*ATAD3A* gene fusions result in congenital pontocerebellar hypoplasia. A family with a similar deletion and clinical presentation was also recently reported (Harel *et al.*, 2016). In a cohort of 169 cases of pontocerebellar hypoplasia, 106 were linked to mutations in four genes (*TSEN54*, *TSEN2*, *TSEN34* and *RARS2*) (Namavar *et al.*, 2011). The identification of five pontocerebellar hypoplasia families with different ethnicities and *ATAD3B*/*ATAD3A* gene fusions suggests ATAD3 defects could underlie a significant portion of the remaining cases lacking a molecular diagnosis, especially those with associated cortical abnormalities.

The *ATAD3* gene cluster is likely to have been generated via consecutive gene duplication events and the extensive regions of direct repeats predispose to non-allelic homologous recombination and an increased frequency of genomic re-arrangements such as deletions and interlocus gene conversion. The highly repetitive sequences can result in erroneous calling of copy number in SNP arrays due to a paucity of unique SNP markers and cross hybridization of these few markers. Similar issues complicate mapping and detection of *ATAD3* single nucleotide variants and copy number changes by whole exome sequencing, where standard pipelines do not routinely detect most copy number variants. Indeed, SNP array and whole exome sequencing of Subject S3 DNA in a diagnostic laboratory were initially reported as normal. Following diagnosis of Subject S1a, SNP array data for the 1p36.33 region from over 50 000 individuals were reanalysed, flagging the likely homozygous deletion in Subject S3.

Hence, the detection of pathogenic mutations in the *ATAD3* genes is compromised and re-interrogation of previous SNP or exome data for this region is warranted for unsolved cases with relevant phenotypes. Homozygous deletions in the region can be identified from exome sequencing by visual inspection of the aligned read data through absence of coverage (Supplementary Fig. 11), indicating that more specialized calling algorithms should be able to recognize the abnormality in the future. Base level resolution of the breakpoints will, however, in most cases require long read sequencing technology to overcome homology and mapping issues.

### Aberrant mtDNA organization and cholesterol homeostasis in CNS defects associated with ATAD3 deficiency

Neurological dysfunction and developmental impairment are features of many mitochondrial disorders, but the pathogenic mechanisms are poorly understood. The current findings suggest that cholesterol could be a key factor in the pathology linked to ATAD3 defects, a view supported by the cerebellar dysfunction present in cholesterol-related genetic diseases. Hypoplasia of the cerebellum is classically associated with Smith-Lemli-Opitz syndrome (MIM 270400), a recessive disorder of cholesterol synthesis, and cerebellar dysfunction is a feature of Niemann-Pick disease Type C. On the other hand, the mtDNA abnormalities evident in Niemann-Pick disease Type C (Fig. 6) and *ATAD3* deletion disorders (Fig. 4) suggest these could be the primary driver of the cerebellar pathologies.

Mitochondria also play an important role in steroidogenesis. Purkinje cells are the main sites of neurosteroid production in the brain (Tsutsui and Yamazaki, 1995; Usui *et al.*, 1995) and progesterone and its metabolite, allopregnanolone, are high in neonatal life, when cerebellar circuits form in mammals to promote dendritic growth, spine formation and maintenance of the Purkinje cells (Sakamoto *et al.*, 2002). Hence, a shortage of the products of cholesterol provides a plausible alternative explanation for the cerebellar pathology accompanying the *ATAD3* deletions.

In contrast to the fatal infantile *ATAD3B*/*ATAD3A* gene fusion defects, recessive and *de novo* dominant *ATAD3A* missense mutations are associated with milder forms of cerebellar dysfunction and a broader range of neurological and multisystem disorders (Harel *et al.*, 2016; Cooper *et al.*, 2017). This suggests that *ATAD3A* missense mutations result in loss of a subset of ATAD3’s functions, or cause it to malfunction, rather than the severe protein deficiency caused by biallelic deletions in the *ATAD3A* locus. Subject S5 differs from other reported individuals with ATAD3 defects in having biallelic genomic rearrangements of the *ATAD3* locus but with a phenotype more similar to that caused by *ATAD3A* missense mutations. This presumably results from the complicated rearrangement in one allele allowing expression of one wild-type *ATAD3A* allele but affecting expression of both ATAD3B alleles.

### Mitochondria, ATAD3 and cholesterol homeostasis

The perturbations of cholesterol homeostasis associated with ATAD3 deficiency suggest mitochondria are a key organelle affecting cholesterol metabolism and that ATAD3 regulates the supply of cholesterol to the mitochondria. That the mitochondrial signal for cholesterol biosynthesis precipitated by ATAD3 deficiency overrides those of the remainder of the cell, where there is no apparent shortage of cholesterol, speaks to the importance of maintaining mitochondrial cholesterol levels within strict limits. The impact on the mtDNA of perturbed cholesterol metabolism (Figs 4–6) suggests insertion of the sterol in the inner mitochondrial membrane is important for mtDNA replication and segregation, which occur at mitochondrial–endoplasmic reticulum junctions (Murley *et al.*, 2013; Lewis *et al.*, 2016). Given the central role of the endoplasmic reticulum in cholesterol metabolism (Iaea and Maxfield, 2015), such contact sites are the obvious route for cholesterol channelling to mitochondria, and the ATAD3 associated with the mtDNA and the inner mitochondrial membrane (He *et al.*, 2007) is ideally placed to ensure cholesterol is concentrated here. Low or dispersed cholesterol in the inner mitochondrial membrane would be expected to alter its rigidity and thereby impede mtDNA segregation (Fig. 7H).

Cholesterol and mitochondrial integrity have both been linked to neurodegeneration (Karasinska and Hayden, 2011). Hitherto these were seen as distinct ideas, which can now be accommodated in a single hypothesis, where defects in either cholesterol metabolism or mitochondrial dysfunction will adversely impact the other. It will therefore be of considerable interest to learn how mtDNA organization and maintenance operate in a broad range of neurodegenerative disorders.

## Supplementary Material

Supplementary Data
