# Supplementary material for: ATAD3 gene cluster deletions cause cerebellar dysfunction associated with altered mitochondrial DNA and cholesterol metabolism
Source: Brain. 2017 May 24;140(6):1595–610. doi: 10.1093/brain/awx094 (PMC5445257; doi:10.1093/brain/awx094)
Supplement: Supplementary Data [file awx094_Supp.pdf]

Supplemental Data

ATAD3 gene cluster deletions cause cerebellar dysfunction associated with altered mitochondrial DNA and cholesterol metabolism

Radha Desai, Ann E. Frazier, Romina Durigon, Harshil Patel, Aleck W. Jones, Ilaria Dalla Rosa, Nicole J. Lake, Alison G. Compton, Hayley S. Mountford, Elena J Tucker, Alice L.R. Mitchell, Deborah Jackson, Abdul Sesay, Miriam Di Re, Lambert P. van den Heuvel, Derek Burke, David Francis, Sebastian Lunke, George McGillivray, Simone Mandelstam, Fanny Mochel, Boris Keren, Claude Jardel, Anne M. Turner, P. Ian Andrews, Jan Smeitink, Johannes N. Spelbrink, Simon J. Heales, Masakazu Kohda, Akira Ohtake, Kei Murayama, Yasushi Okazaki, Anne Lombès, Ian J. Holt, David R. Thorburn and Antonella Spinazzola.

Clinical Descriptions

Family 1

Subjects 1a (female) and 1b (male) (S1a and S1b in Figure 1A) were affected siblings born to Australian parents, who are first cousins of Iranian descent. The first pregnancy (S1a) was complicated by polyhydramnios, reduced fetal movement and breech presentation, which prompted Caesarian section at 38 weeks. The baby had reduced respiratory effort and central cyanosis, and was intubated and ventilated. Head circumference was on the 10th centile and birth weight was 2.12 kg, below the 10th centile. She had bilateral corneal clouding, micrognathia, bilateral talipes, clasped thumbs and a single palmar crease. She showed no spontaneous movement, minor dystonic response to pain and absent Moro, grasp and suck reflexes. Fundi were normal but for indistinct discs. Absent eye opening or eye movements, absent jaw jerk, absent gag reflex and minimal respiratory effort suggested severe hindbrain dysfunction. She also showed diffuse hyper-reflexia. EEG showed burst-suppression pattern, consistent with a severe encephalopathy. MRI showed cerebellar and brainstem hypoplasia, simplified sulcation and gyration, more marked in the frontal regions, thin corpus callosum, diffusely abnormal white matter, and increased extra-

axial fluid and ex-vacuo ventriculomegaly implying brain atrophy (Figure 1B). Serum lactates were persistently elevated (2.7 - 6.2 mmol/L - N <2.0 mmol/L) and lactate was also increased in the urine metabolic screen. Other abnormal features included mild thrombocytopenia and hypoalbuminemia. Respiratory support was withdrawn and she died at 4 days of age. At post-mortem, external brain examination revealed a small brain, indistinct gyration especially in the temporal lobes and marked pontocerebellar hypoplasia. Microscopy revealed normal cortical lamination with mild superficial neuronal loss and diffuse cortical gliosis; moderate neuronal loss and gliosis of the basal ganglia; disorganized hippocampal architecture; rarefied supratentorial white matter with diffuse edema and mild gliosis; severe neuronal loss and gliosis throughout the midbrain, pons and medulla and malformed gliotic olivary nuclei; and near complete loss of the external granule, Purkinje and internal granule cells of the cerebellum with severe gliosis and cavitation in cerebellar white matter. Areas of vascular proliferation reminiscent of Leigh Syndrome (MIM 256000) were prominent in the midbrain, pons, medulla, cerebellum and spinal cord. The eyes had corneal edema, a total loss of ganglion cells, including macula, stunted photoreceptors, and mild atrophy of retinal pigmentary epithelium, suggestive of a mitochondrial disorder. Skeletal muscle showed excessive variation in fiber size, Type II fiber predominance and large, coarse, somewhat sparse mitochondria, without ragged red fibers or cytochrome C oxidase negative fibers. Other organs were normal.

In the next pregnancy, reduced fetal movements were noted at 33 weeks and the male fetus (S1b) died intrapartum during a lengthy labor. Two subsequent pregnancies resulted in healthy female and male babies, with the last one undergoing prenatal diagnosis by SNP array, which excluded a homozygous copy number variant in the *ATAD3* gene cluster.

Family 2

Subject 2 (S2 in Figure 1A) has been described previously (de Koning et al., 1999); she presented at 33 weeks gestation as the first child of second cousin Dutch parents. The pregnancy was complicated by polyhydramnios and lack of fetal movements. She had no spontaneous movements, multiple contractures with some dysmorphic features, severe encephalopathy, with brain MRI consistent with pontocerebellar hypoplasia and died at 5 days of age.

Family 3

Subject 3 (S3 in Figure 1A) was born to consanguineous Australian parents of Indian origin, who had previously had a healthy 4-year-old girl. The mother's pregnancy was uncomplicated with low risk first-trimester screening and a normal 20-week morphology scan. At 34 weeks' gestation, increased fundal height was noted and follow-up ultrasound imaging at 36+3 weeks identified polyhydramnios (Amniotic Fluid Index 30.4) and edema of the fetal chest and abdominal wall. Brain changes were not identified. Cardiotocography was non-reassuring and a male infant was delivered by emergency caesarean section. The infant (S3) was apneic at delivery requiring positive pressure ventilation and <60 seconds of chest compressions for a low initial heart rate. Apgar scores were 4^{1} and 7^{5}. On admission to the special care unit, birth weight was 3092 g (50th – 75th centile) and head circumference 33 cm (25th to 50th centile). Length was not recorded. The infant required intubation and ventilation for failure to establish spontaneous respiration and was transferred to a perinatal intensive care unit. Dysmorphic features were noted, including adducted thumbs, mild camptodactyly, single palmar crease bilaterally, short palpebral fissures, undescended testes and a posteriorly placed anus. The neurological examination showed dystonic posturing with extension of the lower limbs, crossed lower limb adductors, lower limb hypertonia and reduced deep tendon reflexes. Chest x-ray and echocardiography were normal. Significant seizure activity was noted at 8 hours of age, necessitating phenobarbitone treatment. A burst suppression pattern persisted on aEEG monitoring. Early cranial ultrasound demonstrated dilatation of the lateral ventricles and a suspicion of a significant congenital brain anomaly. Brain MRI was undertaken on Day 1 and revealed marked pontocerebellar hypoplasia with extensive supratentorial white matter and cortical abnormalities, including simplified gyration and ex-vacuo dilatation of the lateral ventricles (Figure 1B).

Routine biochemical analysis was unremarkable with normoglycemia and normal acid base balance. Septic work-up was negative. On Day 2, after discussion with his parents, care was redirected to palliation and he died 90 minutes after withdrawal of ventilatory support. There was no spontaneous respiration demonstrated. Postmortem examination showed a male infant with growth and development in keeping with 36 weeks' gestation with many dysmorphic features. Fresh brain weight was less than the

5th centile. Cortical gyration was simplified for gestational age but the cortical layer was not thickened. Lateral cerebral ventricles appeared enlarged with loss of white matter and thinning of the corpus callosum. Brainstem and cerebellum were strikingly hypoplastic and there was hypoplasia of the optic nerve, basal ganglia and pons. Microscopy of the cerebellum showed extensive hypoplasia of the internal granular layer and no appreciable Purkinje cell layer and diffuse gliosis of the white matter, of the midbrain and medulla. The dentate nucleus was absent but there were no abnormalities of cortical gyration.

Family 4

Subject 4 (S4 in Figure 1A) was born to healthy non-consanguineous Japanese parents. No pregnancy complications were reported prior to decreased fetal movement being noted at 37 weeks gestation. Following spontaneous delivery at 37+5 weeks, the neonate showed few movements, had prominent cyanosis and did not cry. He was immediately intubated and spontaneous respiration was never established. Birth weight was 2,596 g and Apgar scores were 6/6 at birth. The limbs were rigid in extension. He exhibited the absence of spontaneous eye opening, poor movement and flat electroencephalography. He also showed concentric hypertrophy at birth. He died at 7 months and 10 days of age from heart failure precipitated by an infection. Brain MRI showed cerebral, cerebellar and brain stem atrophy, simplified gyral pattern in the frontal lobes, and hemorrhage from bilateral lateral ventricles at 3 days of age. Prenatal hypoxic-ischemic encephalopathy was initially suspected. MRS in the patient indicated a decrease in NAA and Cr, and an increase in lactate. His CSF lactate was elevated (3.4 mmol/L lactate; 0.12 mmol/L pyruvate; L/P ratio: 26.5) at 5 days of age, whereas lactate in blood was normal (1 mmol/L lactate). Newborn mass screening showed a normal range of blood amino acids and urinary organic acids.

Family 5

Subject 5 (S5 in Figure 1A) was born to unrelated French parents and differed markedly from the other cases in surviving to adulthood. The subject is presently 30 years old. She displayed moderate mental retardation from childhood, dystonia starting around 13 years of age, psychiatric problems starting in late teens, and

cerebellar ataxia with onset around 25 years of age. Brain imaging shows cerebellar atrophy (Figure 1B) but no abnormalities of cortical gyration.

A

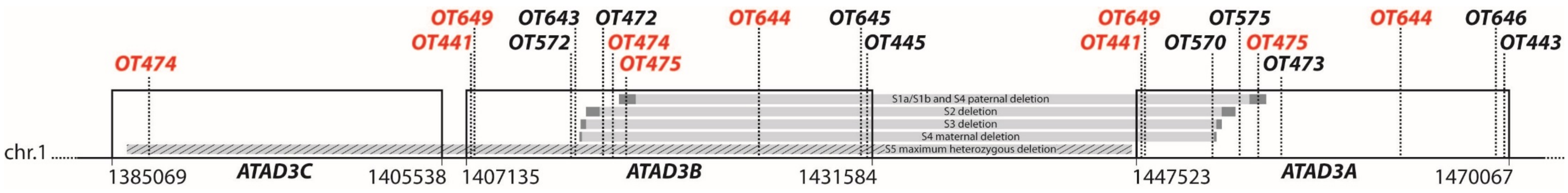

B

```
                          OT644
ATAD3B          GCTCGGCTGACGGAGGGCATGTCGGGCCGGGAGATCGCTCAGCTGGCCGTGTCCTGGCAG
ATAD3A          GCTCGGCTGACGGAGGGCATGTCGGGCCGGGAGATCGCTCAGCTGGCCGTGTCCTGGCAG
                ************************************************************

ATAD3B          GCCACGGCATATGCCTCCAAGGACGGGGTCCTCACTGAGGCCATGATGGACGCCTGTGTG
ATAD3A          GCCACGGCGTATGCCTCCGAGGACGGGGTCCTGACCGAGGCCATGATGGACACCCGCGTG
                ******** ********* ************* ** *************** ** * ***
                                  OT645
ATAD3B          CAAGATGCTGTCCAGCAGTACCGACAGAAGATGCGCTGGCTGAAGGCGGAGGGGCCTGGG
ATAD3A          CAAGATGCTGTCCAGCAGCACCAGCAGAAGATGTGCTGGCTGAAGGCGGAAGGGCCTGGG
                ****************** ***  ********* **************** *********
                                  OT646
```

C

Primer	Sequence	Orientation	Region of chromosome (hg19)	Gene[a]
OT441	5’-AGGACAAATGGAGCAACTTCG	FWD	chr1:1,407,395-1,407,415<br>chr1:1,447,779-1,447,799	*ATAD3B* and *ATAD3A*
OT443	5’-CCTCCTCCCTCCTCTCTCAG	REV	chr1:1,469,944-1,469,963	*ATAD3A*

OT445	5’-GCCCCACTTCTGTCTAGTCCT	REV	chr1:1,431,422-1,431,442	*ATAD3B*
OT472	5’-GGTCTCTGGGTCTATGAGAAAAGC	FWD	chr1:1,415,790-1,415,813	*ATAD3B*
OT473	5’-AGCTTGAGAAGGGAGGAGAAGC	REV	chr1:1,456,147-1,456,168	*ATAD3A*
OT474	5’-GTCACCCGTGTCTGTGTCAG	FWD	chr1:1,387,269-1,387,288<br>chr1: 1,416,090-1,416,109	*ATAD3C* and *ATAD3B*
OT475	5’-CAGAGGTGTCTTCCGAGAGG	REV	chr1:1,416,466-1,416,485<br>chr1: 1,454,520-1,454,539	*ATAD3B* and *ATAD3A*
OT570	5’-TGTGCCTGCCTTGTTTCATA	FWD	chr1:1,452,082-1,452,101	*ATAD3A*
OT572	5’-GAGGGGGTCTTCTTCACAT	FWD	chr1:1,413,006-1,413,025	*ATAD3B*
OT575	5’-GTATGTTTGCGGGTGTGTGA	REV	chr1:1,453,511-1,453,530	*ATAD3A*
OT643	5’-TCATCACAGTCCAAAAGTGAGC	FWD	chr1:1,413,716-1,413,737	*ATAD3B*
OT644	5’-TGACGGAGGGCATGTCGG	FWD	chr1:1,425,999-1,426,016<br>chr1:1,464,659-1,464,676	*ATAD3B* and *ATAD3A*
OT645	5’-AGCGCATCTTCTGTCGGTAC	REV	chr1:1,430,942-1,430,961	*ATAD3B*
OT646	5’-AGCACATCTTCTGCTGGTGC	REV	chr1:1,469,383-1,469,402	*ATAD3A*
OT649	5’- CTGTCGGCAGCCACTTCC	FWD	chr1:1,407,556-1,407,575<br>chr1:1,447,940-1,447,959	*ATAD3B* and *ATAD3A*

[a]Some of the DNA oligonucleotides are not specific and bind to several *ATAD3* regions, as indicated.

Figure S1. Sequences and map positions of primers to the *ATAD3* region on chromosome 1. (A) *ATAD3C*, *ATAD3B* and *ATAD3A* genes drawn roughly to scale, with coordinates corresponding to build hg19. Primers that anneal to multiple regions within *ATAD3* are indicated in red. Deleted regions found within reported subjects are indicated by light gray boxes, and unresolved breakpoint regions indicated by dark gray boxes. **(B)**

An alignment of the *ATAD3B* and *ATAD3A* transcripts used in the qRT-PCR assay to determine the relative expression of *ATAD3A* and *ATAD3B* in human fibroblasts. Primers were designed to span an exon-exon junction (exon 15/16, highlighted in blue), where the reverse primer determines the specificity of the amplicon. Sanger sequencing confirmed amplicon specificity. Primer sequences are indicated in red and labeled. DNA sequence variants are highlighted in yellow. **(C)** PCR primer sequences.

A

```
                       rs777586714  rs111612395  rs553559133  rs373408190  rs541102985
chr1:1454138-1454187   GCCTGCACATTCGGGCACCGTCACC-----CCCCGCAAACGGGCACCGTC-----ACACC
chr1:1416071-1416130   GACTGCCGTCCCAGCCGATGTCACCCGTGTCTGTGTCAGGGTGCGGCGTCTGCAGGTCCC
S1a                    GACTGCCGTCCCAGCCGATGTCACCCGTGTCTGTGTCAGGGTGCGGCGTCTGCAGGTCCC
S4 paternal            GACTGCCGTCCCAGCCGATGTCACCCGTGTCTGTGTCAGGGTGCGGCGTCTGCAGGTCCC
                       * ****     * * *   ******     *   *  *  * **  ****        **

                       rs529684857  rs546887358  rs150710180*  rs139052076*  rs114928482*  rs373250285  rs113814750*  rs138680043  rs552132238  rs557549524  rs377665552  rs540673644
chr1:1454188-1454245   CCGCAAACGGGCACACTCACCCCCCTGC-ACACTCGGGCACAGTCATCCCCCGCACACA-
chr1:1416131-1416190   CAGGTGCCCAGGACGCTTGGAGTTCTGTGGTCCTGGGGCGGACGCAACCTCTGGATTGGT
S1a                    CAGGTGCCCAGGACGCTTGGAGTTCTGTGGTCCTGGGGCGGACGCAACCTCTGGATTGGT
S4 paternal            CAGGTGCCCAGGACGCTTGGAGTTCTGTGGTCCTGGGGCGGACGCAACCTCTGGATTGGT
                       * *    *  * ** **       ***     ** ****  *  ** ** * * *

                       rs757541501  rs780047550  rrs750441299  rs749282295
                                        1454260
                                        1416206                                   5
chr1:1454246-1454303   --TGGGCACAGTCACAGGTTTTAAAGGCTTTTCTCTTTTTCTGCGGCTTCTTCTCAGCAA
chr1:1416191-1416249   GTTGAGCA-TTTTTCTGGTTTTAAAGGCTTTTCTCTTTTTCTGCGGCTTCTTCTCAGCAA
S1a                    GTTGAGCA-TTTTTCTGGTTTTAAAGGCTTTTCTCTTTTTCTGCGGCTTCTTCTCAGCAA
S4 paternal            GTTGAGCA-TTTTTCTGGTTTTAAAGGCTTTTCTCTTTTTCTGCGGCTTCTTCTCAGCAA
                         ** ***   *  * ********************************************

chr1:1454304-1454363   CTTCTCAATGAGGAGAATTTACGGAAGCAGGAGGAGTCCGTGCAGAAGCAGGAAGCCATG
chr1:1416250-1416309   CTTCTCAATGAGGAGAATTTACGGAAGCAGGAGGAGTCCGTGCAGAAGCAGGAAGCCATG
S1a                    CTTCTCAATGAGGAGAATTTACGGAAGCAGGAGGAGTCCGTGCAGAAGCAGGAAGCCATG
S4 paternal            CTTCTCAATGAGGAGAATTTACGGAAGCAGGAGGAGTCCGTGCAGAAGCAGGAAGCCATG
                       ************************************************************

                                                                             1454423
                                                                             1416369
chr1:1454364-1454423   CGGCGAGGTAGGCTGTCTGCTCTCCTGGCTGGGGCGGAGGTGGCGGGGGCTGCTTGTGGA
chr1:1416310-1416369   CGGCGAGGTAGGCTGTCTGCTCTCCTGGCTGGGGCGGAGGTGGCGGGGGCTGCTTGTGGA
S1a                    CGGCGAGGTAGGCTGTCTGCTCTCCTGGCTGGGGCGGAGGTGGCGGGGGCTGCTTGTGGA
S4 paternal            CGGCGAGGTAGGCTGTCTGCTCTCCTGGCTGGGGCGGAGGTGGCGGGGGCTGCTTGTGGA
                       ************************************************************

                       rs147532057/
                       rs1153096*
chr1:1454424-1454483   CCCGGCGTGCACTCTGAGCCTGAGTTCTGCCGCCCGGCCCCTCATAGCTACCAGTGCAGT
chr1:1416370-1416429   TCCGGCGTGCACTCTGAGCCTGAGTTCTGCCGCCCGGCCCCTCATAGCTACCAGTGCAGT
S1a                    CCCGGCGTGCACTCTGAGCCTGAGTTCTGCCGCCCGGCCCCTCATAGCTACCAGTGCAGT
S4 paternal            CCCGGCGTGCACTCTGAGCCTGAGTTCTGCCGCCCGGCCCCTCATAGCTACCAGTGCAGT
                        ***********************************************************

                       rs1149269/              rs370448805*/
                       rs371215775*            rs1149270
chr1:1454484-1454543   GGGCGAGGCCTGCTGGGGCTCCGCGGGTGGGGCTGCCTCTCGGAAGACACCTCTGTCTG
chr1:1416430-1416489   GGGCGAGGCCTGCTGGGGCTCTGCGGGTGGGGCTCCCTCTCGGAAGACACCTCTGTCTG
S1a                    GGGCGAGGCCTGCTGGGGCTCCGCGGGTGGGGCTGCCTCTCGGAAGACACCTCTGTCTG
S4 paternal            GGGCGAGGCCTGCTGGGGCTCCGCGGGTGGGGCTGCCTCTCGGAAGACACCTCTGTCTG
                       ********************* ************ *************************

chr1:1454544-1454603   CGAGTGGACGCCAGGATCTGTTCAGGGAGGGCAGGAGCTGCTTCACTTCATGGGAAGTAC
chr1:1416490-1416549   CGAGTGGACGCCAGGATCTGTTCAGGGAGGGCAGGAGCTGCTTCACTTCATGGGAAGTAC
S1a                    CGAGTGGACGCCAGGATCTGTTCAGGGAGGGCAGGAGCTGCTTCACTTCATGGGAAGTAC
S4 paternal            CGAGTGGACGCCAGGATCTGTTCAGGGAGGGCAGGAGCTGCTTCACTTCATGGGAAGTAC
                       ************************************************************

chr1:1454604-1454663   AGGGGCCTTTTTTTTTTTTTTGAGACGGAGTCTCGCTCTGTCACCCAGGCAGGAGTGCAA
chr1:1416550-1416609   AGGGGCCTTTTTTTTTTTTTTGAGACGGAGTCTCGCTCTGTCACCCAGGCAGGAGTGCAA
S1a                    AGGGGCCTTTTTTTTTTTTTTGAGACGGAGTCTCGCTCTGTCACCCAGGCAGGAGTGCAA
S4 paternal            AGGGGCCTTTTTTTTTTTTTTGAGACGGAGTCTCGCTCTGTCACCCAGGCAGGAGTGCAA
                       ************************************************************

chr1:1454664-1454723   TAGCACGATCTCAGCTCACTGCAACCTCTGCCTCCCAGGTTTAAGCAATTCTCCTGCCTC
chr1:1416610-1416669   TAGCACGATCTCAGCTCACTGCAACCTCTGCCTCCCAGGTTTAAGCAATTCTCCTGCCTC
S1a                    TAGCACGATCTCAGCTCACTGCAACCTCTGCCTCCCAGGTTTAAGCAATTCTCCTGCCTC
S4 paternal            TAGCACGATCTCAGCTCACTGCAACCTCTGCCTCCCAGGTTTAAGCAATTCTCCTGCCTC
                       ************************************************************

chr1:1454724-1454783   AGCCTCCCGAGTAGCTGGGATTATAGGCTCCCGCCACCACGCCCAGCTAATTTTTTTGTA
chr1:1416670-1416729   AGCCTCCCGAGTAGCTGGGATTATAGGCTCCCGCCACCACGCCCAGCTAATTTTTTTGTA
S1a                    AGCCTCCCGAGTAGCTGGGATTATAGGCTCCCGCCACCACGCCCAGCTAATTTTTTTGTA
S4 paternal            AGCCTCCCGAGTAGCTGGGATTATAGGCTCCCGCCACCACGCCCAGCTAATTTTTTTGTA
                       ************************************************************

chr1:1454784-1454843   TCTTCAGTAGAGAAAGGGTTTCACTGTGTTGGCCAGGCTGGTCTTGAACTTCTTGATCTC
chr1:1416730-1416789   TCTTCAGTAGAGAAAGGGTTTCACTGTGTTGGCCAGGCTGGTCTTGAACTTCTTGATCTC
S1a                    TCTTCAGTAGAGAAAGGGTTTCACTGTGTTGGCCAGGCTGGTCTTGAACTTCTTGATCTC
S4 paternal            TCTTCAGTAGAGAAAGGGTTTCACTGTGTTGGCCAGGCTGGTCTTGAACTTCTTGATCTC
                       ************************************************************
```

```
chr1:1454844-1454903     ATTATCCGCCTGCCTTGGCCTTCCACAGTGCTGGGATTACAGGCGTGAGCCTCTGCGTTC
chr1:1416790-1416849     ATTATCCGCCTGCCTTGGCCTTCCACAGTGCTGGGATTACAGGCGTGAGCCTCTGCGTTC
S1a                      ATTATCCGCCTGCCTTGGCCTTCCACAGTGCTGGGATTACAGGCGTGAGCCTCTGCGTTC
S4 paternal              ATTATCCGCCTGCCTTGGCCTTCCACAGTGCTGGGATTACAGGCGTGAGCCTCTGCGTTC
                         ************************************************************

chr1:1454904-1454963     TGCCTAGAACATGGGTCTTTACTGTCCTGGTTTCAGTGGGGATCACAGGTATTTGGTGCC
chr1:1416850-1416909     TGCCTAGAACATGGGTCTTTACTGTCCTGGTTTCAGTGGGGATCACAGGTATTTGGTGCC
S1a                      TGCCTAGAACATGGGTCTTTACTGTCCTGGTTTCAGTGGGGATCACAGGTATTTGGTGCC
S4 paternal              TGCCTAGAACATGGGTCTTTACTGTCCTGGTTTCAGTGGGGATCACAGGTATTTGGTGCC
                         ************************************************************

chr1:1454964-1455023     ATGTGGCATTTGTTGGCGAGTGCTCCAGGCAAACGTCTGTCACCACTCTTCACCGTGGGT
chr1:1416910-1416969     ATGTGGCATTTGTTGGCGAGTGCTCCAGGCAAACGTCTGTCACCACTCTTCACCGTGGGT
S1a                      ATGTGGCATTTGTTGGCGAGTGCTCCAGGCAAACGTCTGTCACCACTCTTCACCGTGGGT
S4 paternal              ATGTGGCATTTGTTGGCGAGTGCTCCAGGCAAACGTCTGTCACCACTCTTCACCGTGGGT
                         ************************************************************

chr1:1455024-1455083     GGGCTTGTGGCGAGGTGTGTGCGTTTAATGTTCAGTAGCCAGGCACGTGGCACGTCACGC
chr1:1416970-1417029     GGGCTTGTGGCGAGGTGTGTGCGTTTAATGTTCAGTAGCCAGGCACGTGGCACGTCACGC
S1a                      GGGCTTGTGGCGAGGTGTGTGCGTTTAATGTTCAGTAGCCAGGCACGTGGCACGTCACGC
S4 paternal              GGGCTTGTGGCGAGGTGTGTGCGTTTAATGTTCAGTAGCCAGGCACGTGGCACGTCACGC
                         ************************************************************

chr1:1455084-1455143     GTGTCTGAGTTCTGACAGCTGTGTTTCTGTGTGAGGGGGGCTTCCTTCAGAACTCCGCGT
chr1:1417030-1417089     GTGTCTGAGTTCTGACAGCTGTGTTTCTGTGTGAGGGGGGCTTCCTTCAGAACTCCGCGT
S1a                      GTGTCTGAGTTCTGACAGCTGTGTTTCTGTGTGAGGGGGGCTTCCTTCAGAACTCCGCGT
S4 paternal              GTGTCTGAGTTCTGACAGCTGTGTTTCTGTGTGAGGGGGGCTTCCTTCAGAACTCCGCGT
                         ************************************************************

chr1:1455144-1455203     TCTGGTTTTTTGCTTCAAAGAGCTCGTCCTGAGAAGTTGCCTAGGCCTCTGGGTCGGATT
chr1:1417090-1417149     TCTGGTTTTTTGCTTCAAAGAGCTCGTCCTGAGAAGTTGCCTAGGCCTCTGGGTCGGATT
S1a                      TCTGGTTTTTTGCTTCAAAGAGCTCGTCCTGAGAAGTTGCCTAGGCCTCTGGGTCGGATT
S4 paternal              TCTGGTTTTTTGCTTCAAAGAGCTCGTCCTGAGAAGTTGCCTAGGCCTCTGGGTCGGATT
                         ************************************************************

chr1:1455204-1455263     TCTGCCCTAATCCATGGGCAGGGCCGGCCTGTGGCGCTGTCCCTACCAAGGTCTGTGTGT
chr1:1417150-1417209     TCTGCCCTAATCCATGGGCAGGGCCGGCCTGTGGCGCTGTCCCTACCAAGGTCTGTGTGT
S1a                      TCTGCCCTAATCCATGGGCAGGGCCGGCCTGTGGCGCTGTCCCTACCAAGGTCTGTGTGT
S4 paternal              TCTGCCCTAATCCATGGGCAGGGCCGGCCTGTGGCGCTGTCCCTACCAAGGTCTGTGTGT
                         ************************************************************

                         rs562411025 (T); 1455280 / 1417226; rs531412566/rs2801368 (G)
chr1:1455264-1455289     GTCTGTGGCATGGACCT----------------------------------GTCCGTGGC
chr1:1417210-1417269     GTCTGTGGCACGGGCCTGTCCATGGACTGGGCTTGTCCGTGGAGTGGGTCGGTCCATGGC
S1a                      GTCTGTGGCATGGACCT----------------------------------GTCCGTGGC
S4 paternal              GTCTGTGGCATGGACCT----------------------------------GTCCGTGGC
                         ********** ** ***                                  **** ****

                         rs547936524 (A); rs2015388*/rs557136555 (T); rs2015387* (T); rs368400191 (G); rs191228211/rs542213104 (C)
chr1:1455290-1455349     CTTAGCCTATTGGCGGCGTGGGCCTGTCTGTGGCGTGGGCCTGTCCGTGGCCTTAGCCTG
chr1:1417270-1417329     CTTAGCCTGTTGGTGGCGTGGGCCGGTCCACGGCATGGGCCTGTCTGTGGCGTGGGCCGG
S1a                      CTTAGCCTATTGGCGGCGTGGGCCTGTCTGTGGCGTGGGCCTGTCCGTGGCCTTAGCCTG
S4 paternal              CTTAGCCTATTGGCGGCGTGGGCCGGTCCGTGGCGTGGGCCTGTCCGTGGCCTTAGCCTG
                         ******** **** ********** ***   *** ********** ***** *  *** *

                         rs705580* (T)
chr1:1455350-1455392     TCAGCAGTGTGGGCCTGTCC-----------------ATGGCGTGGGCCGGTCCGTGGCA
chr1:1417330-1417389     TCCGTGGTGCGGGCCTGTCCGTGGCCTTAGCCTGTTGGTGGCGTGGGCCGGTCCGTGGCA
S1a                      TCAGCAGTGTGGGCCTGTCC-----------------ATGGCGTGGGCCGGTCCGTGGCA
S4 paternal              TCAGCAGTGTGGGCCTGTCC-----------------ATGGCGTGGGCCGGTCCGTGGCA
                         ** *  *** **********                  **********************

chr1:1455393-1455452     TGGGCCTGTCTGTGGCGTTGGTCTGTCCGTGGCGTGGGCCGGTCCGTGGCGTGGGCCGGT
chr1:1417390-1417449     TGGGCCTGTCTGTGGCGTTGGTCTGTCCGTGGCGTGGGCCGGTCCGTGGCGTGGGCCGGT
S1a                      TGGGCCTGTCTGTGGCGTTGGTCTGTCCGTGGCGTGGGCCGGTCCGTGGCGTGGGCCGGT
S4 paternal              TGGGCCTGTCTGTGGCGTTGGTCTGTCCGTGGCGTGGGCCGGTCCGTGGCGTGGGCCGGT
                         ************************************************************
```

B chr1: 1454138-1455452 and chr1:1416071-1417449

SNP	Minor Allele Frequency (MAF)
rs150710180	G=0.0290
rs139052076	A=0.0290
rs114928482	T=0.0162
rs373250285	T=0.0002
rs113814750	C=0.0715
rs138680043	T=0.0002
rs552132238	T=0.0002
rs557549524	A=ND
rs377665552	T=0.0002
rs540673644	A=0.0028
rs757541501	T=ND
rs780047550	-TT=ND
rs750441299	A=ND
rs749282295	C=ND
rs147532057	T=ND
rs1153096	C=0.4371
rs1149269	T=ND
rs371215775	C=0.0713
rs370448805	C=0.0321
rs1149270	G=ND
rs562411025	C=0.0008
rs531412566	A=0.0002
rs2801368	G=ND
rs547936524	G=0.0002
rs2015388	T=0.2859
rs557136555	A=0.0002
rs2015387	T=0.2867
rs368400191	G=ND
rs191228211	T=0.0016
rs542213104	C=0.0012
rs705580	T=0.4832

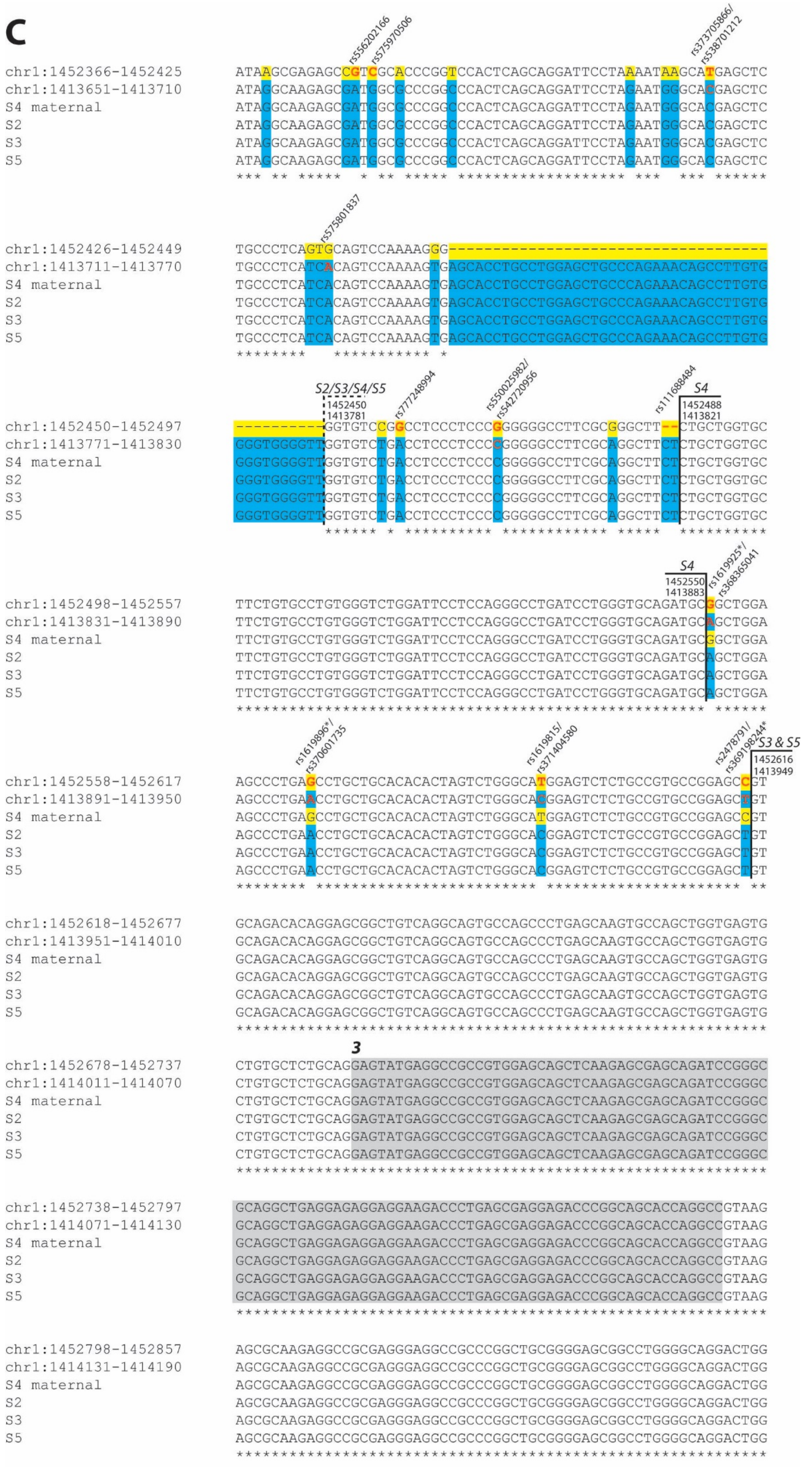
C
rs556202166
rs575970506
rs373705866/
rs538701212
chr1:1452366-1452425 ATAAGCGAGAGCCGTCGCACCCGGTCCACTCAGCAGGATTCCTAAAATAAGCATGAGCTC
chr1:1413651-1413710 ATAGGCAAGAGCGATGGCGCCCGGCCCACTCAGCAGGATTCCTAGAATGGGCACGAGCTC
S4 maternal ATAGGCAAGAGCGATGGCGCCCGGCCCACTCAGCAGGATTCCTAGAATGGGCACGAGCTC
S2 ATAGGCAAGAGCGATGGCGCCCGGCCCACTCAGCAGGATTCCTAGAATGGGCACGAGCTC
S3 ATAGGCAAGAGCGATGGCGCCCGGCCCACTCAGCAGGATTCCTAGAATGGGCACGAGCTC
S5 ATAGGCAAGAGCGATGGCGCCCGGCCCACTCAGCAGGATTCCTAGAATGGGCACGAGCTC
*** ** ***** * ** ***** ****************** *** *** ******
rs575801837
chr1:1452426-1452449 TGCCCTCAGTGCAGTCCAAAAGGG------------------------------------
chr1:1413711-1413770 TGCCCTCATCACAGTCCAAAAGTGAGCACCTGCCTGGAGCTGCCCAGAAACAGCCTTGTG
S4 maternal TGCCCTCATCACAGTCCAAAAGTGAGCACCTGCCTGGAGCTGCCCAGAAACAGCCTTGTG
S2 TGCCCTCATCACAGTCCAAAAGTGAGCACCTGCCTGGAGCTGCCCAGAAACAGCCTTGTG
S3 TGCCCTCATCACAGTCCAAAAGTGAGCACCTGCCTGGAGCTGCCCAGAAACAGCCTTGTG
S5 TGCCCTCATCACAGTCCAAAAGTGAGCACCTGCCTGGAGCTGCCCAGAAACAGCCTTGTG
******** *********** *
S2/S3/S4/S5
1452450
1413781
rs777248994
rs550025982/
rs542720956
rs111688484
S4
1452488
1413821
chr1:1452450-1452497 ----------GGTGTCCGGCCTCCCTCCCGGGGGGCCTTCGCGGGCTT--CTGCTGGTGC
chr1:1413771-1413830 GGGTGGGGTTGGTGTCTGACCTCCCTCCCCGGGGGCCTTCGCAGGCTTCTCTGCTGGTGC
S4 maternal GGGTGGGGTTGGTGTCTGACCTCCCTCCCCGGGGGCCTTCGCAGGCTTCTCTGCTGGTGC
S2 GGGTGGGGTTGGTGTCTGACCTCCCTCCCCGGGGGCCTTCGCAGGCTTCTCTGCTGGTGC
S3 GGGTGGGGTTGGTGTCTGACCTCCCTCCCCGGGGGCCTTCGCAGGCTTCTCTGCTGGTGC
S5 GGGTGGGGTTGGTGTCTGACCTCCCTCCCCGGGGGCCTTCGCAGGCTTCTCTGCTGGTGC
****** * ********** ************ ***** *********
S4
1452550
1413883
rs1619925*/
rs368365041
chr1:1452498-1452557 TTCTGTGCCTGTGGGTCTGGATTCCTCCAGGGCCTGATCCTGGGTGCAGATGCGGCTGGA
chr1:1413831-1413890 TTCTGTGCCTGTGGGTCTGGATTCCTCCAGGGCCTGATCCTGGGTGCAGATGCAGCTGGA
S4 maternal TTCTGTGCCTGTGGGTCTGGATTCCTCCAGGGCCTGATCCTGGGTGCAGATGCGGCTGGA
S2 TTCTGTGCCTGTGGGTCTGGATTCCTCCAGGGCCTGATCCTGGGTGCAGATGCAGCTGGA
S3 TTCTGTGCCTGTGGGTCTGGATTCCTCCAGGGCCTGATCCTGGGTGCAGATGCAGCTGGA
S5 TTCTGTGCCTGTGGGTCTGGATTCCTCCAGGGCCTGATCCTGGGTGCAGATGCAGCTGGA
*** ******
rs1619896*/
rs370601735
rs1619815/
rs371404580
rs2478791/
rs369198244*
S3 & S5
1452616
1413949
chr1:1452558-1452617 AGCCCTGAGCCTGCTGCACACACTAGTCTGGGCATGGAGTCTCTGCCGTGCCGGAGCCGT
chr1:1413891-1413950 AGCCCTGAACCTGCTGCACACACTAGTCTGGGCACGGAGTCTCTGCCGTGCCGGAGCTGT
S4 maternal AGCCCTGAGCCTGCTGCACACACTAGTCTGGGCATGGAGTCTCTGCCGTGCCGGAGCCGT
S2 AGCCCTGAACCTGCTGCACACACTAGTCTGGGCACGGAGTCTCTGCCGTGCCGGAGCTGT
S3 AGCCCTGAACCTGCTGCACACACTAGTCTGGGCACGGAGTCTCTGCCGTGCCGGAGCTGT
S5 AGCCCTGAACCTGCTGCACACACTAGTCTGGGCACGGAGTCTCTGCCGTGCCGGAGCTGT
******** ************************* ********************** **
chr1:1452618-1452677 GCAGACACAGGAGCGGCTGTCAGGCAGTGCCAGCCCTGAGCAAGTGCCAGCTGGTGAGTG
chr1:1413951-1414010 GCAGACACAGGAGCGGCTGTCAGGCAGTGCCAGCCCTGAGCAAGTGCCAGCTGGTGAGTG
S4 maternal GCAGACACAGGAGCGGCTGTCAGGCAGTGCCAGCCCTGAGCAAGTGCCAGCTGGTGAGTG
S2 GCAGACACAGGAGCGGCTGTCAGGCAGTGCCAGCCCTGAGCAAGTGCCAGCTGGTGAGTG
S3 GCAGACACAGGAGCGGCTGTCAGGCAGTGCCAGCCCTGAGCAAGTGCCAGCTGGTGAGTG
S5 GCAGACACAGGAGCGGCTGTCAGGCAGTGCCAGCCCTGAGCAAGTGCCAGCTGGTGAGTG
****
3
chr1:1452678-1452737 CTGTGCTCTGCAGGAGTATGAGGCCGCCGTGGAGCAGCTCAAGAGCGAGCAGATCCGGGC
chr1:1414011-1414070 CTGTGCTCTGCAGGAGTATGAGGCCGCCGTGGAGCAGCTCAAGAGCGAGCAGATCCGGGC
S4 maternal CTGTGCTCTGCAGGAGTATGAGGCCGCCGTGGAGCAGCTCAAGAGCGAGCAGATCCGGGC
S2 CTGTGCTCTGCAGGAGTATGAGGCCGCCGTGGAGCAGCTCAAGAGCGAGCAGATCCGGGC
S3 CTGTGCTCTGCAGGAGTATGAGGCCGCCGTGGAGCAGCTCAAGAGCGAGCAGATCCGGGC
S5 CTGTGCTCTGCAGGAGTATGAGGCCGCCGTGGAGCAGCTCAAGAGCGAGCAGATCCGGGC
****
chr1:1452738-1452797 GCAGGCTGAGGAGAGGAGGAAGACCCTGAGCGAGGAGACCCGGCAGCACCAGGCCGTAAG
chr1:1414071-1414130 GCAGGCTGAGGAGAGGAGGAAGACCCTGAGCGAGGAGACCCGGCAGCACCAGGCCGTAAG
S4 maternal GCAGGCTGAGGAGAGGAGGAAGACCCTGAGCGAGGAGACCCGGCAGCACCAGGCCGTAAG
S2 GCAGGCTGAGGAGAGGAGGAAGACCCTGAGCGAGGAGACCCGGCAGCACCAGGCCGTAAG
S3 GCAGGCTGAGGAGAGGAGGAAGACCCTGAGCGAGGAGACCCGGCAGCACCAGGCCGTAAG
S5 GCAGGCTGAGGAGAGGAGGAAGACCCTGAGCGAGGAGACCCGGCAGCACCAGGCCGTAAG
****
chr1:1452798-1452857 AGCGCAAGAGGCCGCGAGGGAGGCCGCCCGGCTGCGGGGAGCGGCCTGGGGCAGGACTGG
chr1:1414131-1414190 AGCGCAAGAGGCCGCGAGGGAGGCCGCCCGGCTGCGGGGAGCGGCCTGGGGCAGGACTGG
S4 maternal AGCGCAAGAGGCCGCGAGGGAGGCCGCCCGGCTGCGGGGAGCGGCCTGGGGCAGGACTGG
S2 AGCGCAAGAGGCCGCGAGGGAGGCCGCCCGGCTGCGGGGAGCGGCCTGGGGCAGGACTGG
S3 AGCGCAAGAGGCCGCGAGGGAGGCCGCCCGGCTGCGGGGAGCGGCCTGGGGCAGGACTGG
S5 AGCGCAAGAGGCCGCGAGGGAGGCCGCCCGGCTGCGGGGAGCGGCCTGGGGCAGGACTGG
****

```
chr1:1452858-1452917   GAGCTGGGTGTGGTCCCGGGGCACTCTGGAGTCAGCCATTAGAGCTGCCCTCGGAACGGC
chr1:1414191-1414250   GAGCTGGGTGTGGTCCCGGGGCACTCTGGAGTCAGCCATTAGAGCTGCCCTCGGAACGGC
S4 maternal            GAGCTGGGTGTGGTCCCGGGGCACTCTGGAGTCAGCCATTAGAGCTGCCCTCGGAACGGC
S2                     GAGCTGGGTGTGGTCCCGGGGCACTCTGGAGTCAGCCATTAGAGCTGCCCTCGGAACGGC
S3                     GAGCTGGGTGTGGTCCCGGGGCACTCTGGAGTCAGCCATTAGAGCTGCCCTCGGAACGGC
S5                     GAGCTGGGTGTGGTCCCGGGGCACTCTGGAGTCAGCCATTAGAGCTGCCCTCGGAACGGC
                       ************************************************************

chr1:1452928-1452977   CTTGCACAAACGCCTAAGACCTGTAAGGTCCCTCACTGCTGAGCCGGACGGGAGGTCCCC
chr1:1414251-1414310   CTTGCACAAACGCCTAAGACCTGTAAGGTCCCTCACTGCTGAGCCGGACGGGAGGTCCCC
S4 maternal            CTTGCACAAACGCCTAAGACCTGTAAGGTCCCTCACTGCTGAGCCGGACGGGAGGTCCCC
S2                     CTTGCACAAACGCCTAAGACCTGTAAGGTCCCTCACTGCTGAGCCGGACGGGAGGTCCCC
S3                     CTTGCACAAACGCCTAAGACCTGTAAGGTCCCTCACTGCTGAGCCGGACGGGAGGTCCCC
S5                     CTTGCACAAACGCCTAAGACCTGTAAGGTCCCTCACTGCTGAGCCGGACGGGAGGTCCCC
                       ************************************************************

chr1:1452978-1453037   GCGCCTCCCCACGTTTGTGTGAGGCTGATGGCGCGTCGGAGTCCCCGGCGCTCCGCCCAG
chr1:1414311-1414370   GCGCCTCCCCACGTTTGTGTGAGGCTGATGGCGCGTCGGAGTCCCCGGCGCTCCGCCCAG
S4 maternal            GCGCCTCCCCACGTTTGTGTGAGGCTGATGGCGCGTCGGAGTCCCCGGCGCTCCGCCCAG
S2                     GCGCCTCCCCACGTTTGTGTGAGGCTGATGGCGCGTCGGAGTCCCCGGCGCTCCGCCCAG
S3                     GCGCCTCCCCACGTTTGTGTGAGGCTGATGGCGCGTCGGAGTCCCCGGCGCTCCGCCCAG
S5                     GCGCCTCCCCACGTTTGTGTGAGGCTGATGGCGCGTCGGAGTCCCCGGCGCTCCGCCCAG
                       ************************************************************

                                                                                  4
chr1:1453038-1453097   TCGGCCCAGACTGCAGCTCCCGGCTGAGATGTGTCTTTGCCGCCCTCTTCTCCCCCAGAG
chr1:1414371-1414430   TCGGCCCAGACTGCAGCTCCCGGCTGAGATGTGTCTTTGCCGCCCTCTTCTCCCCCAGAG
S4 maternal            TCGGCCCAGACTGCAGCTCCCGGCTGAGATGTGTCTTTGCCGCCCTCTTCTCCCCCAGAG
S2                     TCGGCCCAGACTGCAGCTCCCGGCTGAGATGTGTCTTTGCCGCCCTCTTCTCCCCCAGAG
S3                     TCGGCCCAGACTGCAGCTCCCGGCTGAGATGTGTCTTTGCCGCCCTCTTCTCCCCCAGAG
S5                     TCGGCCCAGACTGCAGCTCCCGGCTGAGATGTGTCTTTGCCGCCCTCTTCTCCCCCAGAG
                       ************************************************************

chr1:1453098-1453157   GGCCCAGTATCAAGACAAGCTGGCCCGGCAGCGCTACGAGGACCAACTGAAGCAGCAGGT
chr1:1414431-1414490   GGCCCAGTATCAAGACAAGCTGGCCCGGCAGCGCTACGAGGACCAACTGAAGCAGCAGGT
S4 maternal            GGCCCAGTATCAAGACAAGCTGGCCCGGCAGCGCTACGAGGACCAACTGAAGCAGCAGGT
S2                     GGCCCAGTATCAAGACAAGCTGGCCCGGCAGCGCTACGAGGACCAACTGAAGCAGCAGGT
S3                     GGCCCAGTATCAAGACAAGCTGGCCCGGCAGCGCTACGAGGACCAACTGAAGCAGCAGGT
S5                     GGCCCAGTATCAAGACAAGCTGGCCCGGCAGCGCTACGAGGACCAACTGAAGCAGCAGGT
                       ************************************************************

chr1:1453158-1453217   GAGCTCAGCCTCCCCTGCGAGGCGCCTGCGTCCCTGAGAACGTAGGTGGCTTTGTGGGAC
chr1:1414491-1414550   GAGCTCAGCCTCCCCTGCGAGGCGCCTGCGTCCCTGAGAACGTAGGTGGCTTTGTGGGAC
S4 maternal            GAGCTCAGCCTCCCCTGCGAGGCGCCTGCGTCCCTGAGAACGTAGGTGGCTTTGTGGGAC
S2                     GAGCTCAGCCTCCCCTGCGAGGCGCCTGCGTCCCTGAGAACGTAGGTGGCTTTGTGGGAC
S3                     GAGCTCAGCCTCCCCTGCGAGGCGCCTGCGTCCCTGAGAACGTAGGTGGCTTTGTGGGAC
S5                     GAGCTCAGCCTCCCCTGCGAGGCGCCTGCGTCCCTGAGAACGTAGGTGGCTTTGTGGGAC
                       ************************************************************

                                                   S3 & S5  rs35772049/
                                                   1453251  rs1153101
                                                   1414584
chr1:1453218-1453277   CAGTCAGTGGGTCAGAGGCCACGGGGCAAGAACGCTGGGGTTGCTGACGGTGGGTGCTAG
chr1:1414551-1414610   CAGTCAGTGGGTCAGAGGCCACGGGGCAAGAACGATGGGGTTGCTGACGGTGGGTGCTAG
S4 maternal            CAGTCAGTGGGTCAGAGGCCACGGGGCAAGAACGCTGGGGTTGCTGACGGTGGGTGCTAG
S2                     CAGTCAGTGGGTCAGAGGCCACGGGGCAAGAACGATGGGGTTGCTGACGGTGGGTGCTAG
S3                     CAGTCAGTGGGTCAGAGGCCACGGGGCAAGAACGCTGGGGTTGCTGACGGTGGGTGCTAG
S5                     CAGTCAGTGGGTCAGAGGCCACGGGGCAAGAACGCTGGGGTTGCTGACGGTGGGTGCTAG
                       ********************************** *************************

chr1:1453278-1453337   AGCAGGGGAAACTACTCGGACAGACACGCACCAGCACACGTGTACAGGCACACATGCAGA
chr1:1414611-1414670   AGCAGGGGAAACTACTCGGACAGACACGCACCAGCACACGTGTACAGGCACACATGCAGA
S4 maternal            AGCAGGGGAAACTACTCGGACAGACACGCACCAGCACACGTGTACAGGCACACATGCAGA
S2                     AGCAGGGGAAACTACTCGGACAGACACGCACCAGCACACGTGTACAGGCACACATGCAGA
S3                     AGCAGGGGAAACTACTCGGACAGACACGCACCAGCACACGTGTACAGGCACACATGCAGA
S5                     AGCAGGGGAAACTACTCGGACAGACACGCACCAGCACACGTGTACAGGCACACATGCAGA
                       ************************************************************

                       rs2801370/        rs2801369*/       rs7531221/  rs1153102*  S2
                       rs537721104       rs79585054*       rs2649577               1453382
                                                                                   1414715
chr1:1453338-1453397   CGTGTGCACACATGTACATGGAGACACAGGCACCTACCCACACGGACACACACTCCTCGC
chr1:1414671-1414730   TGTGTGCACACATGTACACGGAGACACAGGCACCTGCCCACACAGACACACACTCCTCGC
S4 maternal            TGTGTGCACACATGTACACGGAGACACAGGCACCTGCCCACACGGACACACACTCCTCGC
S2                     TGTGTGCACACATGTACACGGAGACACAGGCACCTGCCCACACAGACACACACTCCTCGC
S3                     CGTGTGCACACATGTACATGGAGACACAGGCACCTACCCACACGGACACACACTCCTCGC
S5                     TGTGTGCACACATGTACATGGAGACACAGGCACCTGCCCACACGGACACACACTCCTCGC
                        **************** **************** ******* *****************

                                  S2
                                  1453409  rs557784367/                 rs372297233
                                  1414742  rs2936658
chr1:1453398-1453457   ACACACACTCCCAGCACACACAGACAGGTGCACCCACTTCTGCACACACGGGCACACACA
chr1:1414731-1414784   ACACACACTCCCGGCA------GACAGGCACACACACCCCTGCACACATGGGCACACACA
S4 maternal            ACACACACTCCCAGCACACACAGACAGGTGCACCCACTTCTGCACACACGGGCACACACA
S2                     ACACACACTCCCAGCACACACAGACAGGTGCACCCACTTCTGCACACACGGGCACACACA
S3                     ACACACACTCCCAGCACACACAGACAGGTGCACCCACTTCTGCACACACGGGCACACACA
S5                     ACACACACTCCCAGCACACACAGACAGGTGCACCCACTTCTGCACACACGGGCACACACA
                       ************ ***      ******  *** ***  ********* ***********
```

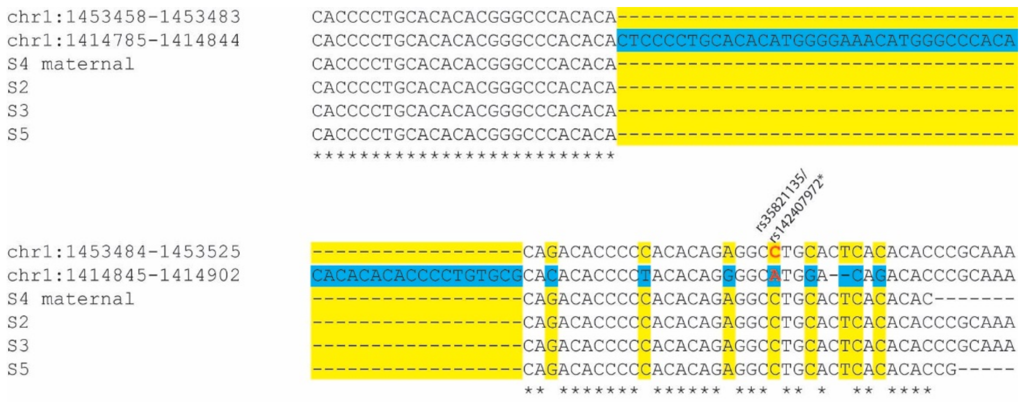

D chr1: 1452366-1453525 and chr1:1413651-1414902

SNP	Minor Allele Frequency (MAF)
rs556202166	A=0.0004
rs575970506	T=0.0002
rs373705866	C=0.0008
rs538701212	T=0.0002
rs575801837	G=0.0004
rs777248994	G=ND
rs550025982	A=0.0008
rs542720956	T=0.0004
rs111688484	+CT=ND
rs1619925	A=0.0268
rs368365041	G=0.0047
rs1619896	A=0.0091
rs370601735	G=0.0027
rs1619815	C=0.0004
rs371404580	T=0.0008
rs2478791	T=0.0038
rs369198244	C=0.0194
rs35772049	A=0.0002
rs1153101	C=ND
rs2801370	T=ND
rs537721104	C=ND
rs2801369	C=0.0917
rs79585054	T=0.0595
rs7531221	A=0.2889
rs2649577	A=ND
rs1153102	G=0.4359
rs557784367	G=0.0004
rs2936658	A=ND
rs372297233	T=ND
rs35821135	T=ND
rs142407972	G=0.0274

Figure S2. gDNA sequence alignments to determine breakpoints. (A) The long range PCR amplicon covering the deletion breakpoints generated from S1a and S4 (paternal) genomic DNA was subjected to Sanger sequencing and multiple overlapping reads combined to create one contiguous sequence. This was aligned with reference sequence regions (hg19) corresponding to *ATAD3B* (chr1:1,416,071-1,417,449) and *ATAD3A* (chr1:1,454,138-1,455,452) using the multiple sequence alignment tool MUSCLE (http://www.ebi.ac.uk/Tools/msa/muscle/). Bases specific to *ATAD3B* are highlighted blue or specific to *ATAD3A* are highlighted yellow. Any specific bases that are SNPs are labeled red and dbSNP numbers indicated (http://www.ncbi.nlm.nih.gov/SNP/) (* indicates an allele frequency >0.005). Exons (gray box) located within the region are numbered. The breakpoints are predicted to fall within the regions chr1:1,416,206-1,416,369 and chr1:1,454,260-1,454,423, based on the reference sequence (solid lines). However, there is some ambiguity about this interpretation due to the presence of 4 polymorphisms that indicates the regions containing the breakpoints may extend out to chr1:1,417,226 and chr1:1,455,280 (dashed line). **(B)** The minor allele frequency (MAF) for the relevant SNPs from **A**, sourced from ExAC (http://exac.broadinstitute.org/), or 1000 Genomes Project (http://www.1000genomes.org/) if the SNP is located outside of the ExAC calling regions. ND= no data. **(C)** The long-range PCR products covering the deletion breakpoints generated from S2, S3, S4 (maternal) and S5 genomic DNA were analyzed and aligned to reference sequence regions corresponding to *ATAD3B* (chr1:1,413,651-1,414,902) and *ATAD3A* (chr1:1,452,366-1,453,525) as in **A**. Based on the reference sequence, the predicted breakpoints regions for S2 are chr1: 1,414,715-1,414,742 and chr1: 1,453,382-1,453,409, for S3 are chr1:1,413,949-1,414,584 and chr1:1,452,616-1,453,251, and for S4 (paternal) are chr1: 1,413,821- 1,413,883 and chr1: 1,452,488- 1,452,550 (solid lines). Again, the presence of multiple polymorphisms in the region may indicate the regions containing the breakpoints are wider than predicted (dashed lines). The apparent deletion detected in S5 has the same predicted breakpoints as S3, however the array data and cDNA evidence indicates that it is more likely to be a complicated chromosomal rearrangement of *ATAD3B* rather than a deletion. SNP haplotyping of the 1p36.33 LCSH regions for the Dutch subject S2 and the Indian subject S3 showed no evidence of haplotype homology around the *ATAD3* cluster, confirming these similar deletions arose from separate *de novo* events rather than the subjects being related to a common ancestor. **(D)** The MAFs for the relevant SNPs from **C**, sourced as described in **B**.

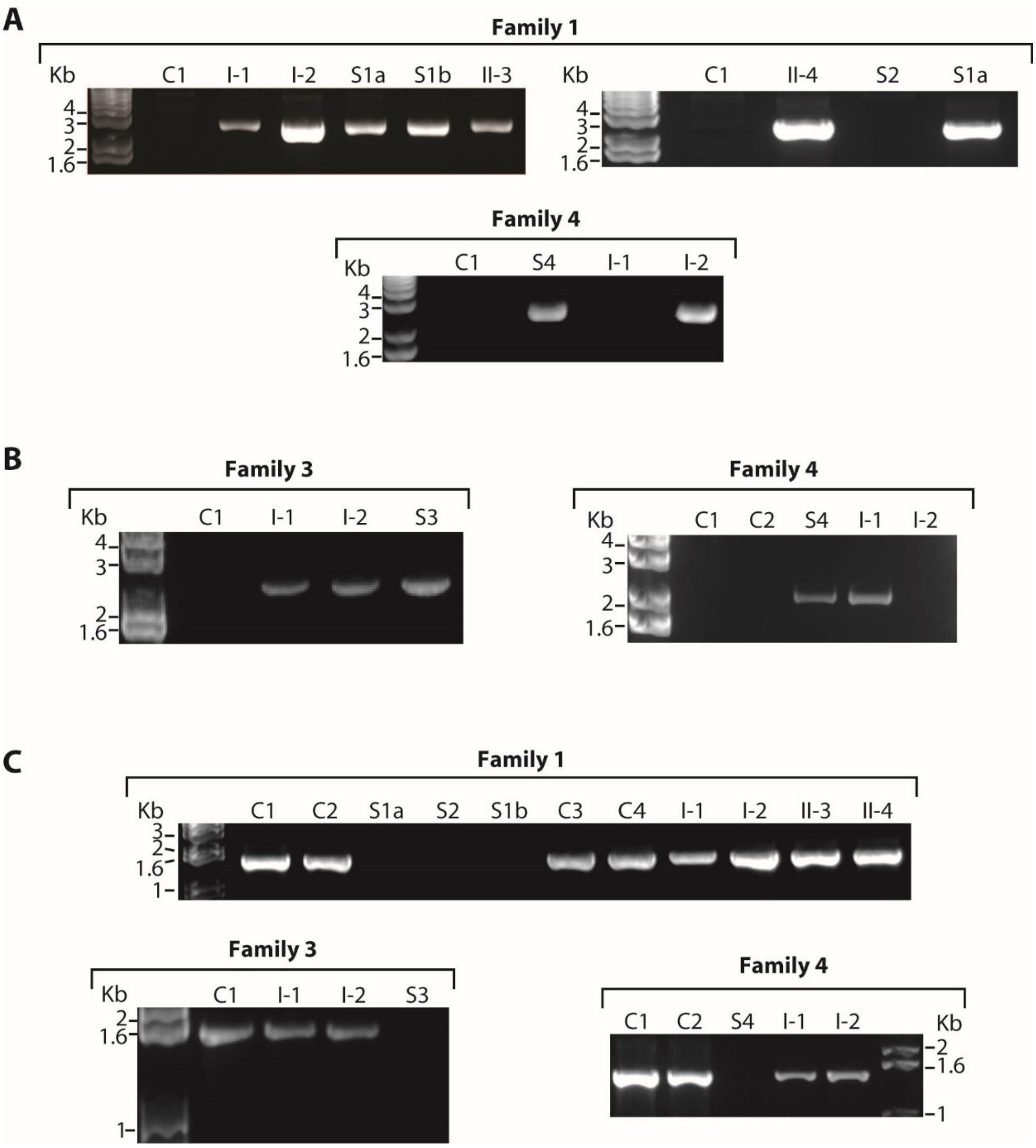

Figure S3. Detection of carrier status in family members. **(A)** Long-range PCR was performed on genomic DNA from controls and family members of S1a/S1b (family 1, Figure 1A) and S4 (family 4, Figure 1A) using primers OT472 and OT473, which flank the *ATAD3* deletion breakpoints detected in S1a by array CGH. **(B)** Long-range PCR was performed on genomic DNA from controls and family members of S3 (family 3, Figure 1A) and S4 (family 4) using primers OT572 and OT575, which flank the *ATAD3* deletion breakpoints detected in S2 by array CGH. **(C)** A control PCR was performed using primers OT570 and OT575 on genomic DNA from controls and family members of S1a/S1b (family 1), S3 (family 3) and S4 (family 4). Primer OT570 is located within the deleted *ATAD3* region from S1a/S1b, S2, S3 and S4, therefore no product is amplified.

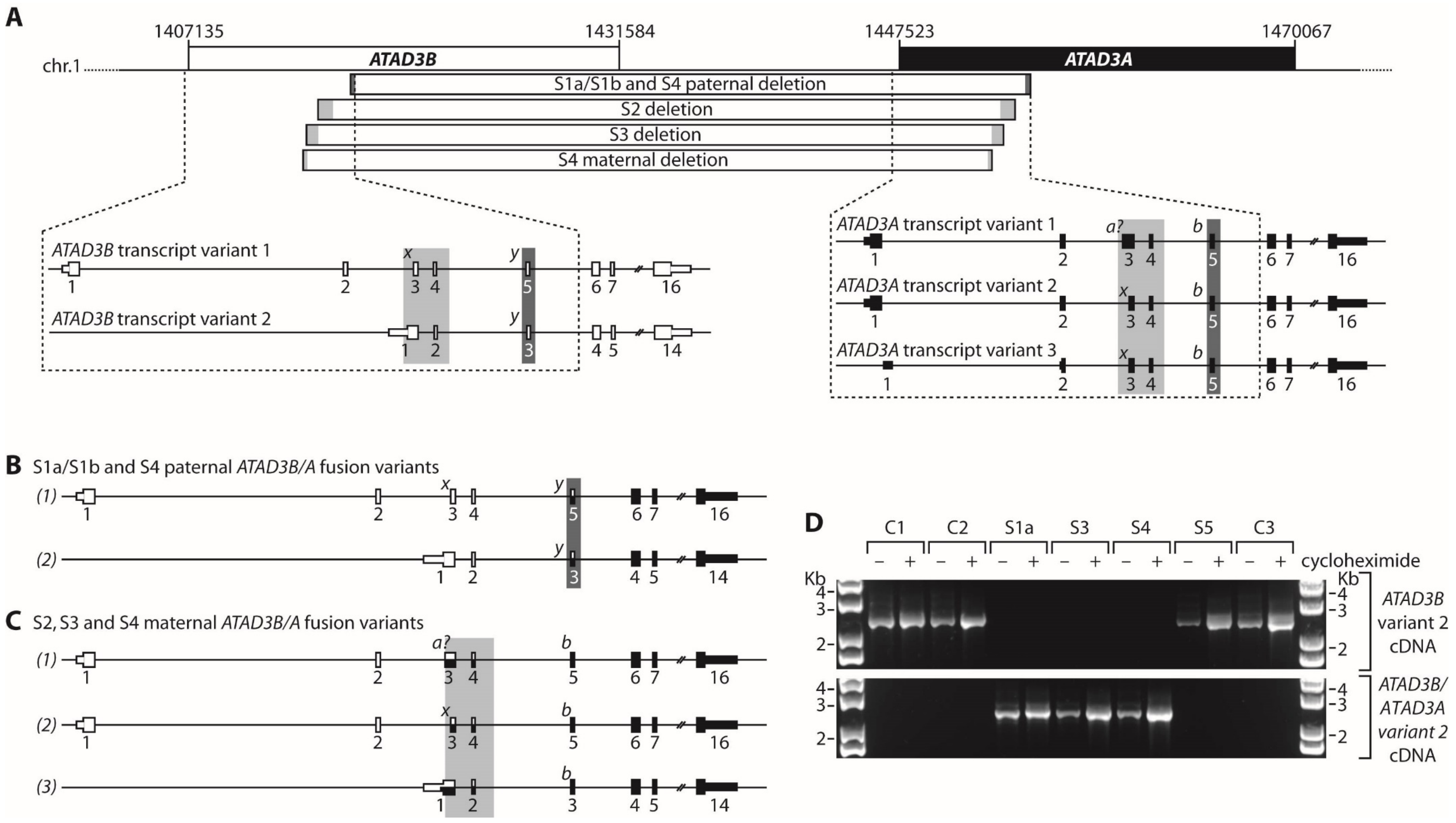
A
1407135
1431584
1447523
1470067
chr.1
ATAD3B
ATAD3A
S1a/S1b and S4 paternal deletion
S2 deletion
S3 deletion
S4 maternal deletion
ATAD3B transcript variant 1
ATAD3B transcript variant 2
ATAD3A transcript variant 1
ATAD3A transcript variant 2
ATAD3A transcript variant 3
B
S1a/S1b and S4 paternal ATAD3B/A fusion variants
C
S2, S3 and S4 maternal ATAD3B/A fusion variants
D
C1
C2
S1a
S3
S4
S5
C3
cycloheximide
Kb
ATAD3B variant 2 cDNA
ATAD3B/ ATAD3A variant 2 cDNA

Figure S4. ***ATAD3B*** **and** ***ATAD3A*** **gene structure and predicted variants. (A)** Deletions in chromosome 1 of S1a/S1b, S2, S3 and S4 are indicated (top). Regions corresponding to ambiguous breakpoint boundaries are indicated by dark grey (S1a/S1b and S4 paternal) and light grey (S2, S3 and S4 maternal) boxes. Magnifications of deletion boundaries indicate gene structure within the region. Relevant predicted branch sites are indicated above the genes (*x, y, a, b*). **(B)** Predicted variants for S1a/S1b and S4 paternal *ATAD3B/ATAD3A* fusion gene. It is likely that exon 5 from transcript variant 1 (and exon 3 from variant 2) will use the splicing branch site for *ATAD3B*. **(C)** Predicted variants for S2, S3 and S4 maternal *ATAD3B/ATAD3A* fusion gene. It is unclear if variant *(1)* will be expressed since there are 3 predicted branch sites for *ATAD3A* variant 1 exon 3 of similar prediction value that fall completely (S2 and S3) or partially (S4) within the deletion boundaries. However, two of these share identical sequence to *ATAD3B*. As well, *ATAD3A* variant 1 is not amplified from mRNA obtained from fibroblasts. **(D)** *ATAD3B* variant 2 was amplified from cDNA prepared from controls and S1a, S3, S4 and S5 fibroblasts grown +/- cycloheximide using primers OT643 and OT445. An *ATAD3B/ATAD3A* fusion cDNA (corresponding to predicted fusion variant 2 shown in (B)) was amplified using primers OT643 and OT443.

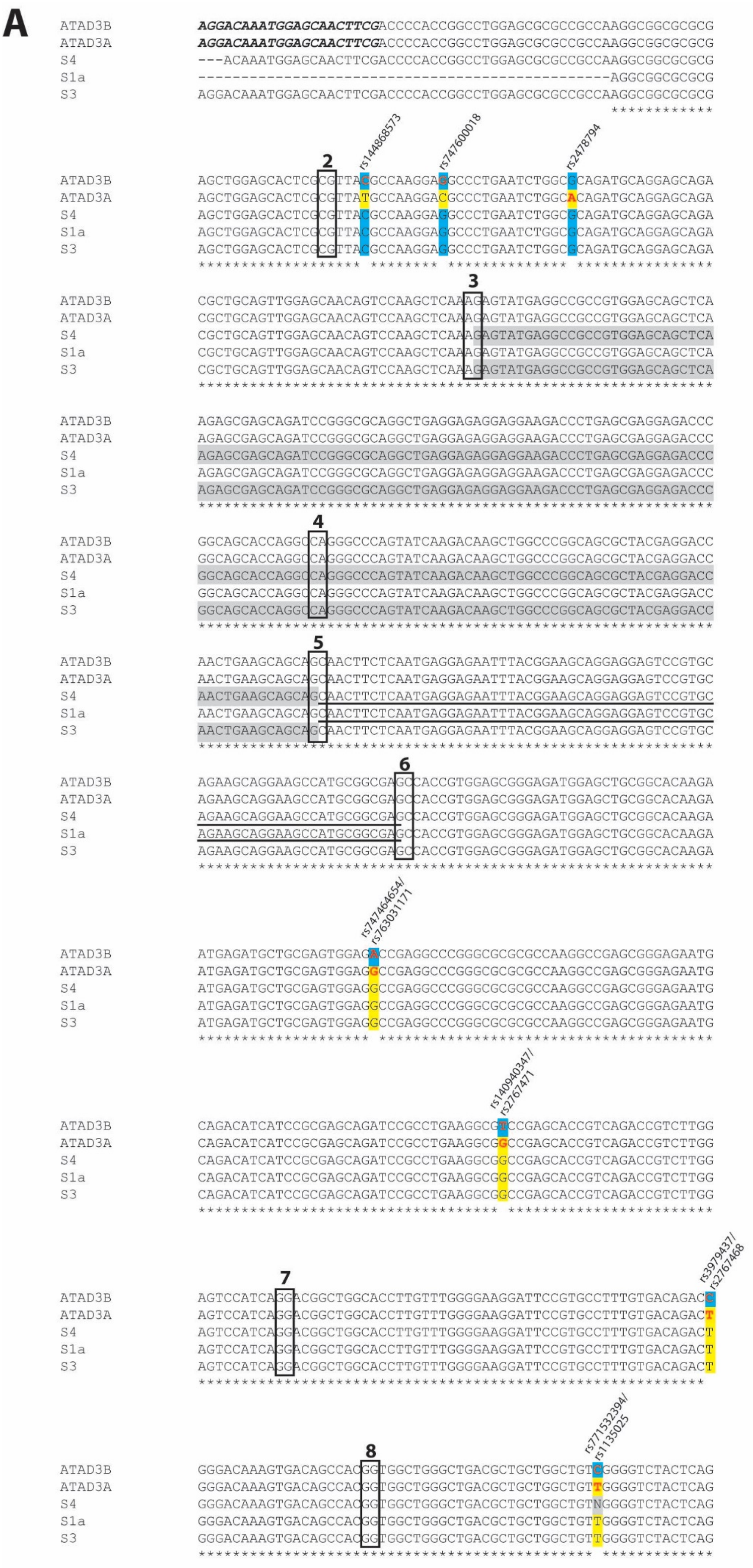
A
ATAD3B AGGACAAATGGAGCAACTTCGACCCCACCGGCCTGGAGCGCGCCGCCAAGGCGGCGCGCG
ATAD3A AGGACAAATGGAGCAACTTCGACCCCACCGGCCTGGAGCGCGCCGCCAAGGCGGCGCGCG
S4 ---ACAAATGGAGCAACTTCGACCCCACCGGCCTGGAGCGCGCCGCCAAGGCGGCGCGCG
S1a --AGGCGGCGCGCG
S3 AGGACAAATGGAGCAACTTCGACCCCACCGGCCTGGAGCGCGCCGCCAAGGCGGCGCGCG
****
2
rs144868573
rs747600018
rs2478794
ATAD3B AGCTGGAGCACTCGCGTTACGCCAAGGAGGCCCTGAATCTGGCGCAGATGCAGGAGCAGA
ATAD3A AGCTGGAGCACTCGCGTTATGCCAAGGACGCCCTGAATCTGGCACAGATGCAGGAGCAGA
S4 AGCTGGAGCACTCGCGTTACGCCAAGGAGGCCCTGAATCTGGCGCAGATGCAGGAGCAGA
S1a AGCTGGAGCACTCGCGTTACGCCAAGGAGGCCCTGAATCTGGCGCAGATGCAGGAGCAGA
S3 AGCTGGAGCACTCGCGTTACGCCAAGGAGGCCCTGAATCTGGCGCAGATGCAGGAGCAGA
******************* ******** ************** ****************
3
ATAD3B CGCTGCAGTTGGAGCAACAGTCCAAGCTCAAAGAGTATGAGGCCGCCGTGGAGCAGCTCA
ATAD3A CGCTGCAGTTGGAGCAACAGTCCAAGCTCAAAGAGTATGAGGCCGCCGTGGAGCAGCTCA
S4 CGCTGCAGTTGGAGCAACAGTCCAAGCTCAAAGAGTATGAGGCCGCCGTGGAGCAGCTCA
S1a CGCTGCAGTTGGAGCAACAGTCCAAGCTCAAAGAGTATGAGGCCGCCGTGGAGCAGCTCA
S3 CGCTGCAGTTGGAGCAACAGTCCAAGCTCAAAGAGTATGAGGCCGCCGTGGAGCAGCTCA
****
ATAD3B AGAGCGAGCAGATCCGGGCGCAGGCTGAGGAGAGGAGGAAGACCCTGAGCGAGGAGACCC
ATAD3A AGAGCGAGCAGATCCGGGCGCAGGCTGAGGAGAGGAGGAAGACCCTGAGCGAGGAGACCC
S4 AGAGCGAGCAGATCCGGGCGCAGGCTGAGGAGAGGAGGAAGACCCTGAGCGAGGAGACCC
S1a AGAGCGAGCAGATCCGGGCGCAGGCTGAGGAGAGGAGGAAGACCCTGAGCGAGGAGACCC
S3 AGAGCGAGCAGATCCGGGCGCAGGCTGAGGAGAGGAGGAAGACCCTGAGCGAGGAGACCC
****
4
ATAD3B GGCAGCACCAGGCCAGGGCCCAGTATCAAGACAAGCTGGCCCGGCAGCGCTACGAGGACC
ATAD3A GGCAGCACCAGGCCAGGGCCCAGTATCAAGACAAGCTGGCCCGGCAGCGCTACGAGGACC
S4 GGCAGCACCAGGCCAGGGCCCAGTATCAAGACAAGCTGGCCCGGCAGCGCTACGAGGACC
S1a GGCAGCACCAGGCCAGGGCCCAGTATCAAGACAAGCTGGCCCGGCAGCGCTACGAGGACC
S3 GGCAGCACCAGGCCAGGGCCCAGTATCAAGACAAGCTGGCCCGGCAGCGCTACGAGGACC
****
5
ATAD3B AACTGAAGCAGCAGCAACTTCTCAATGAGGAGAATTTACGGAAGCAGGAGGAGTCCGTGC
ATAD3A AACTGAAGCAGCAGCAACTTCTCAATGAGGAGAATTTACGGAAGCAGGAGGAGTCCGTGC
S4 AACTGAAGCAGCAGCAACTTCTCAATGAGGAGAATTTACGGAAGCAGGAGGAGTCCGTGC
S1a AACTGAAGCAGCAGCAACTTCTCAATGAGGAGAATTTACGGAAGCAGGAGGAGTCCGTGC
S3 AACTGAAGCAGCAGCAACTTCTCAATGAGGAGAATTTACGGAAGCAGGAGGAGTCCGTGC
****
6
ATAD3B AGAAGCAGGAAGCCATGCGGCGAGCCACCGTGGAGCGGGAGATGGAGCTGCGGCACAAGA
ATAD3A AGAAGCAGGAAGCCATGCGGCGAGCCACCGTGGAGCGGGAGATGGAGCTGCGGCACAAGA
S4 AGAAGCAGGAAGCCATGCGGCGAGCCACCGTGGAGCGGGAGATGGAGCTGCGGCACAAGA
S1a AGAAGCAGGAAGCCATGCGGCGAGCCACCGTGGAGCGGGAGATGGAGCTGCGGCACAAGA
S3 AGAAGCAGGAAGCCATGCGGCGAGCCACCGTGGAGCGGGAGATGGAGCTGCGGCACAAGA
****
rs747464654/
rs763031171
ATAD3B ATGAGATGCTGCGAGTGGAGACCGAGGCCCGGGCGCGCGCCAAGGCCGAGCGGGAGAATG
ATAD3A ATGAGATGCTGCGAGTGGAGGCCGAGGCCCGGGCGCGCGCCAAGGCCGAGCGGGAGAATG
S4 ATGAGATGCTGCGAGTGGAGGCCGAGGCCCGGGCGCGCGCCAAGGCCGAGCGGGAGAATG
S1a ATGAGATGCTGCGAGTGGAGGCCGAGGCCCGGGCGCGCGCCAAGGCCGAGCGGGAGAATG
S3 ATGAGATGCTGCGAGTGGAGGCCGAGGCCCGGGCGCGCGCCAAGGCCGAGCGGGAGAATG
******************** ***************************************
rs140940347/
rs2767471
ATAD3B CAGACATCATCCGCGAGCAGATCCGCCTGAAGGCGTCCGAGCACCGTCAGACCGTCTTGG
ATAD3A CAGACATCATCCGCGAGCAGATCCGCCTGAAGGCGGCCGAGCACCGTCAGACCGTCTTGG
S4 CAGACATCATCCGCGAGCAGATCCGCCTGAAGGCGGCCGAGCACCGTCAGACCGTCTTGG
S1a CAGACATCATCCGCGAGCAGATCCGCCTGAAGGCGGCCGAGCACCGTCAGACCGTCTTGG
S3 CAGACATCATCCGCGAGCAGATCCGCCTGAAGGCGGCCGAGCACCGTCAGACCGTCTTGG
*********************************** ************************
7
rs3979437/
rs2767468
ATAD3B AGTCCATCAGGACGGCTGGCACCTTGTTTGGGGAAGGATTCCGTGCCTTTGTGACAGACC
ATAD3A AGTCCATCAGGACGGCTGGCACCTTGTTTGGGGAAGGATTCCGTGCCTTTGTGACAGACT
S4 AGTCCATCAGGACGGCTGGCACCTTGTTTGGGGAAGGATTCCGTGCCTTTGTGACAGACT
S1a AGTCCATCAGGACGGCTGGCACCTTGTTTGGGGAAGGATTCCGTGCCTTTGTGACAGACT
S3 AGTCCATCAGGACGGCTGGCACCTTGTTTGGGGAAGGATTCCGTGCCTTTGTGACAGACT
****
8
rs771532394/
rs1135025
ATAD3B GGGACAAAGTGACAGCCACGGTGGCTGGGCTGACGCTGCTGGCTGTCGGGGTCTACTCAG
ATAD3A GGGACAAAGTGACAGCCACGGTGGCTGGGCTGACGCTGCTGGCTGTTGGGGTCTACTCAG
S4 GGGACAAAGTGACAGCCACGGTGGCTGGGCTGACGCTGCTGGCTGTNGGGGTCTACTCAG
S1a GGGACAAAGTGACAGCCACGGTGGCTGGGCTGACGCTGCTGGCTGTTGGGGTCTACTCAG
S3 GGGACAAAGTGACAGCCACGGTGGCTGGGCTGACGCTGCTGGCTGTTGGGGTCTACTCAG
** *************

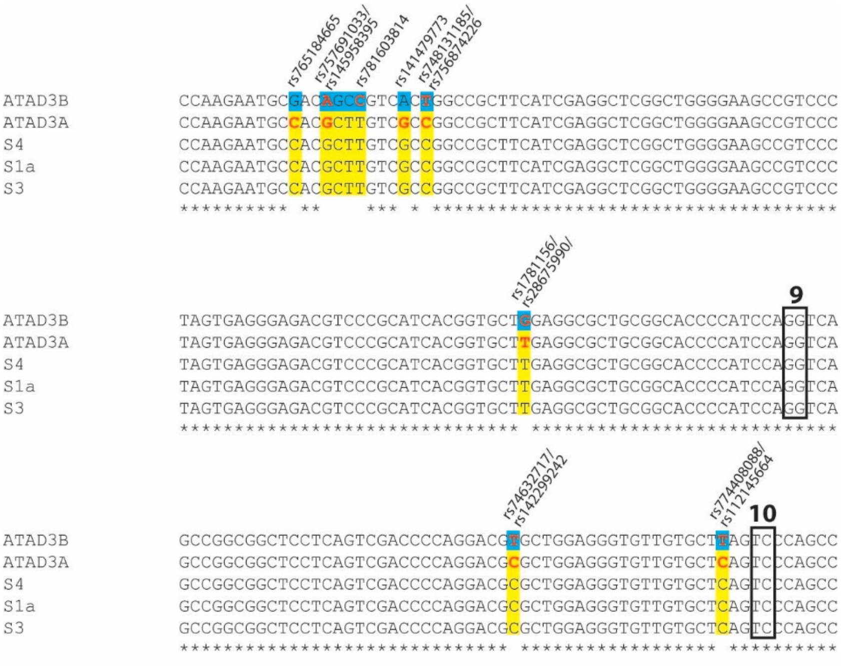
rs765184665
rs757691033/
rs145958395
rs781603814
rs141479773
rs748131185/
rs756874226
ATAD3B CCAAGAATGCGACAGCCGTCACTGGCCGCTTCATCGAGGCTCGGCTGGGGAAGCCGTCCC
ATAD3A CCAAGAATGCCACGCTTGTCGCCGGCCGCTTCATCGAGGCTCGGCTGGGGAAGCCGTCCC
S4 CCAAGAATGCCACGCTTGTCGCCGGCCGCTTCATCGAGGCTCGGCTGGGGAAGCCGTCCC
S1a CCAAGAATGCCACGCTTGTCGCCGGCCGCTTCATCGAGGCTCGGCTGGGGAAGCCGTCCC
S3 CCAAGAATGCCACGCTTGTCGCCGGCCGCTTCATCGAGGCTCGGCTGGGGAAGCCGTCCC
rs1781156/
rs28675990/
9
ATAD3B TAGTGAGGGAGACGTCCCGCATCACGGTGCTGGAGGCGCTGCGGCACCCCATCCAGGTCA
ATAD3A TAGTGAGGGAGACGTCCCGCATCACGGTGCTTGAGGCGCTGCGGCACCCCATCCAGGTCA
S4 TAGTGAGGGAGACGTCCCGCATCACGGTGCTTGAGGCGCTGCGGCACCCCATCCAGGTCA
S1a TAGTGAGGGAGACGTCCCGCATCACGGTGCTTGAGGCGCTGCGGCACCCCATCCAGGTCA
S3 TAGTGAGGGAGACGTCCCGCATCACGGTGCTTGAGGCGCTGCGGCACCCCATCCAGGTCA
rs74632717/
rs142299242
rs774408088/
rs112145664
10
ATAD3B GCCGGCGGCTCCTCAGTCGACCCCAGGACGTGCTGGAGGGTGTTGTGCTTAGTCCCAGCC
ATAD3A GCCGGCGGCTCCTCAGTCGACCCCAGGACGCGCTGGAGGGTGTTGTGCTCAGTCCCAGCC
S4 GCCGGCGGCTCCTCAGTCGACCCCAGGACGCGCTGGAGGGTGTTGTGCTCAGTCCCAGCC
S1a GCCGGCGGCTCCTCAGTCGACCCCAGGACGCGCTGGAGGGTGTTGTGCTCAGTCCCAGCC
S3 GCCGGCGGCTCCTCAGTCGACCCCAGGACGCGCTGGAGGGTGTTGTGCTCAGTCCCAGCC

B

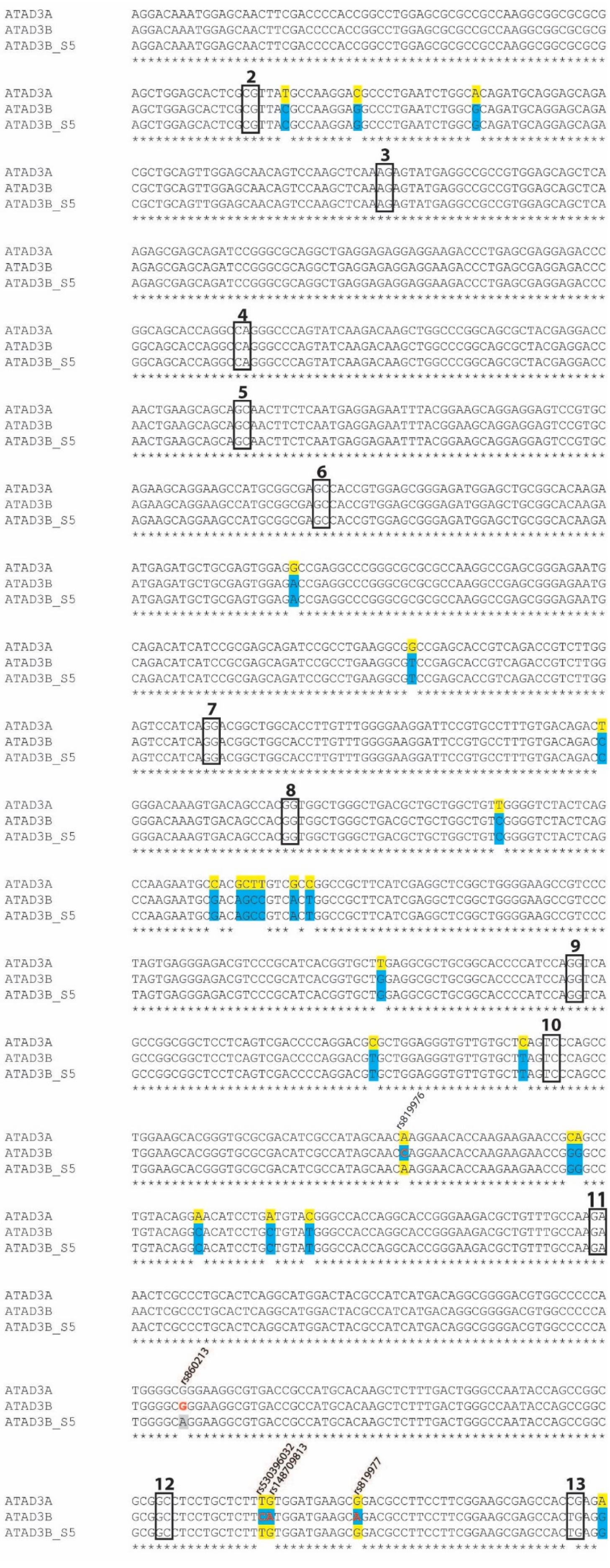
ATAD3A
ATAD3B
ATAD3B_S5
2
3
4
5
6
7
8
9
10
11
12
13
rs819976
rs860213
rs530296032
rs148709813
rs819977

```
ATAD3A          AGATAAGCGAGGACCTCAGGGCCACACTGAACGCCTTCCTGTACCGCACGGGCCAGCACA
ATAD3B          AGATAAGCAAGGACCTCAGAGCCACACTGAACGCCTTCCTGTACCACATGGGCCAACACA
ATAD3B_S5       AGATAAGCAAGGACCTCAGAGCCACACTGAACGCCTTCCTGTACCACATGGGCCAACACA
                ******** ********** ************************* ** ****** ****

                      14
ATAD3A          GCAACAAGTTCATGCTGGTCCTGGCCAGCAACCAACCAGAGCAGTTCGACTGGGCCATCA
ATAD3B          GCAACAAATTCATGCTGGTCCTGGCCAGCAATCTGCCTGAGCAGTTCGACTGTGCCATCA
ATAD3B_S5       GCAACAAATTCATGCTGGTCCTGGCCAGCAATCTGCCTGAGCAGTTCGACTGTGCCATCA
                ******* *********************** *  ** ************** *******

                          rs819980
ATAD3A          ATGACCGCATCAATGAGATGGTCCACTTCGACCTGCCAGGGCAGGAGGAACGGGAGCGCC
ATAD3B          ACAGCCGCATTGACGTGATGGTCCACTTCGACCTGCCGCAGCAGGAGGAGCGGGAGCGCC
ATAD3B_S5       ACAGCCGCATCGACGTGATGGTCCACTTCGACCTGCCGCAGCAGGAGGAGCGGGAGCGCC
                *   ******  * * *********************   ********* **********

                                                                      15
ATAD3A          TGGTGAGAATGTATTTTGACAAGTATGTTCTTAAGCCGGCCACAGAAGGAAAGCAGCGCC
ATAD3B          TGGTGAGACTGCATTTTGACAACTGTGTTCTTAAGCCGGCCACAGAAGGAAAACGGCGCC
ATAD3B_S5       TGGTGAGACTGCATTTTGACAACTGTGTTCTTAAGCCGGCCACAGAAGGAAAACGGCGCC
                ******** ** ********** * *************************** * *****

ATAD3A          TGAAGCTGGCCCAGTTTGACTACGGGAGGAAGTGCTCGGAGGTCGCTCGGCTGACGGAGG
ATAD3B          TGAAGCTGGCCCAGTTTGACTACGGGAGGAAGTGCTCGGAGGTCGCTCGGCTGACGGAGG
ATAD3B_S5       TGAAGCTGGCCCAGTTTGACTACGGGAGGAAGTGCTCGGAGGTCGCTCGGCTGACGGAGG
                ************************************************************

                                                             16
ATAD3A          GCATGTCGGGCCGGGAGATCGCTCAGCTGGCCGTGTCCTGGCAGGCCACGGCGTATGCCT
ATAD3B          GCATGTCGGGCCGGGAGATCGCTCAGCTGGCCGTGTCCTGGCAGGCCACGGCATATGCCT
ATAD3B_S5       GCATGTCGGGCCGGGAGATCGCTCAGCTGGCCGTGTCCTGGCAGGCCACGGCATATGCCT
                **************************************************** *******

ATAD3A          CCGAGGACGGGGTCCTGACCGAGGCCATGATGGACACCCGCGTGCAAGATGCTGTCCAGC
ATAD3B          CCAAGGACGGGGTCCTCACTGAGGCCATGATGGACGCCTGTGTGCAAGATGCTGTCCAGC
ATAD3B_S5       CCAAGGACGGGGTCCTCACTGAGGCCATGATGGACGCCTGTGTGCAAGATGCTGTCCAGC
                ** ************* ** *************** ** * *******************

ATAD3A          AGCACCAGCAGAAGATGTGCTGGCTGAAGGCGGAAGGGCCTGGGCGTGGGGACGAGCCCT
ATAD3B          AGTACCGACAGAAGATGCGCTGGCTGAAGGCGGAGGGGCCTGGGCGCGGGGTCGAGCACC
ATAD3B_S5       AGTACCGACAGAAGATGCGCTGGCTGAAGGCGGAGGGGCCTGGGCGCGGGGTCGAGCACC
                ** ***  ********* **************** *********** **** ***** * 

ATAD3A          CCCCATCCTGAGTCCACAGGGAGATCCACAGCTCACGGAGCCTGGCCGCGGACCCCTCCC
ATAD3B          CCCTATCCGGAGTCCAAGGCGAGACCCTCACCTCATGGAGCCTGGCCACGGACCCCTCCT
ATAD3B_S5       CCCTATCCGGAGTCCAAGGCGAGACCCTCACCTCATGGAGCCTGGCCACGGACCCCTCCT
                *** **** *******  * **** ** ** **** *********** *********** 

ATAD3A          ACCCCTGCCTTGCCGGCCCCTGCACATTTAGGATATGCTCCTGGGTGGGGACTGGGCTGT
ATAD3B          ACCCCTGCCTTGCCGGCCCCTGCACATTTAGGATATGCTCCTGGATGGGGACTGGGCTGT
ATAD3B_S5       ACCCCTGCCTTGCCGGCCCCTGCACATTTAGGATATGCTCCTGGATGGGGACTGGGCTGT
                ******************************************* ****************

ATAD3A          GCCCAGGGCCTCTGTCCCCCAGGATGTCTTGTGGTGCGGGTCGGCCGTTCTGCCCCCCAG
ATAD3B          GCCCAGGGCCTCTGTCCCCCAGGATGTCTTGTGGTGGCGGTCGGCCGTTCTGCCCCCCAG
ATAD3B_S5       GCCCAGGGCCTCTGTCCCCCAGGATGTCTTGTGGTGGCGGTCGGCCGTTCTGCCCCCCAG
                ************************************  **********************

ATAD3A          GGCACCCCCTGTTGTAGGCACTGGCTAGGGAGGGGCAGGCCTCCTTCCTGCCCCTCGAGA
ATAD3B          GGCACCCCCTGTTGTAGGCACTGGCTAGGGAGGGGCAGGCCTCCTTCCTGCCCCTCGAGA
ATAD3B_S5       GGCACCCCCTGTTGTAGGCACTGGCTAGGGAGGGGCAGGCCTCCTTCCTGCCCCTCGAGA
                ************************************************************

ATAD3A          CACTCTTGGGAGATGCATTTTCCGTCTGGCTCACAGGGGGAGGGTGAGGCTTTGCACCCC
ATAD3B          CACTCTTGGGAGATGCATTTTCCGTCTGGCTCACAGGGGGAGGGTGAGGCTTTGTACCCC
ATAD3B_S5       CACTCTTGGGAGATGCATTTTCCGTCTGGCTCACAGGGGGAGGGTGAGGCTTTGTACCCC
                ****************************************************** *****

ATAD3A          AGCCCCTGCCCAGGCCACTGTGAGGGTGGGTGCTGGCTGAGCCCCCGGGGCAGCAGGAGC
ATAD3B          AGCCCCTGCCCAGGCCACTGTGAGGGTGGGTGCTGGCTGAGCCCCTGGGGCAGAAGGAGT
ATAD3B_S5       AGCCCCTGCCCAGGCCACTGTGAGGGTGGGTGCTGGCTGAGCCCCTGGGGCAGAAGGAGT
                ********************************************* ******* ***** 

ATAD3A          CAGGCAGGTGATGTCTTTGTTCTCGGCTCCCACAGCAGAGCCAGGTGAGGGGGCGCCTGC
ATAD3B          GGGGCAGGCGGGGTCTTTGTTCTCGGCTCCCACAGCAGAGCCAGGTGAGGGGGGGCCTGC
ATAD3B_S5       GGGGCAGGCGGGGTCTTTGTTCTCGGCTCCCACAGCAGA---------------------
                  ****** *  ***************************

ATAD3A          CAGGGCCAGACCCAGGTGGGGCAGCCTGAACCCTGCTTCCCCCTGTGGCCGGCATGCCCC
ATAD3B          CAGGACTAGACAGAAGTGGGGCGGCCTGAACCCTGCTTCCAGCCATGGCC----------
ATAD3B_S5       ------------------------------------------------------------

ATAD3A          GATCTTTCACACACTGGTGACCCTGAGAGAGGAGGGAGGAGGGAACCTGGCGGGGGTGTC
ATAD3B          --------------------------------AGGGGCCACGGAACCCGGCAGGGGTGTC
ATAD3B_S5       ------------------------------------------------------------

ATAD3A          TGAGGCCGCACTGTCAGCTGGCCGGTCCAAGCCTGTGGCTGGAGCTGGGGTCTGTTTACC
ATAD3B          TGAGGCCGCCCTGTCAGCTGGCCGGTCCAAGCCTGTGGCTGGAGCTGGTGTGTGTTTATC
ATAD3B_S5       ------------------------------------------------------------

ATAD3A          TAATAAAGTCCCACAGGTGCCTCATTAAAAAAAAAA--------------
ATAD3B          TAATAAAGTCCCACAGGTGCCTCACCAAAAAAAAAAAAAAAAAAAAAAAA
ATAD3B_S5       --------------------------------------------------
```

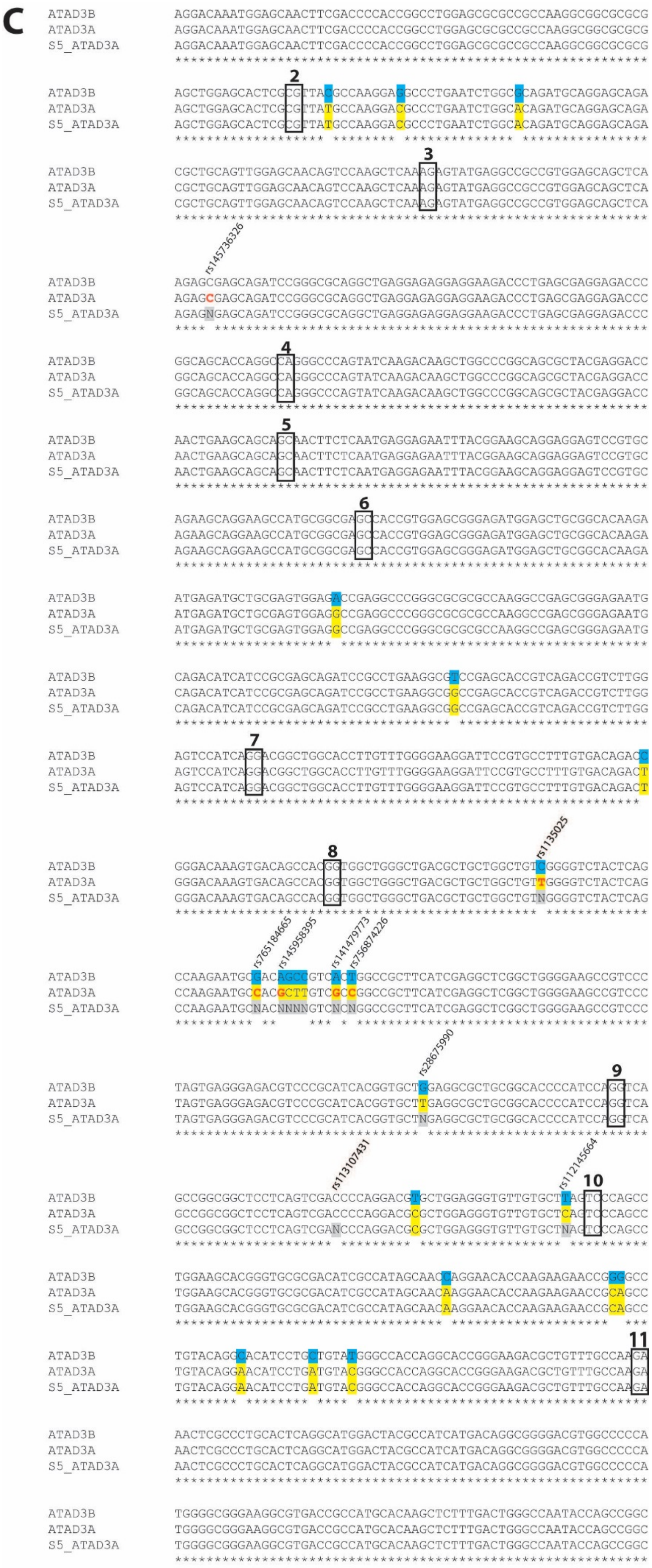
C
ATAD3B AGGACAAATGGAGCAACTTCGACCCCACCGGCCTGGAGCGCGCCGCCAAGGCGGCGCGCG
ATAD3A AGGACAAATGGAGCAACTTCGACCCCACCGGCCTGGAGCGCGCCGCCAAGGCGGCGCGCG
S5_ATAD3A AGGACAAATGGAGCAACTTCGACCCCACCGGCCTGGAGCGCGCCGCCAAGGCGGCGCGCG
2
ATAD3B AGCTGGAGCACTCGCGTTACGCCAAGGAGGCCCTGAATCTGGCGCAGATGCAGGAGCAGA
ATAD3A AGCTGGAGCACTCGCGTTATGCCAAGGACGCCCTGAATCTGGCACAGATGCAGGAGCAGA
S5_ATAD3A AGCTGGAGCACTCGCGTTATGCCAAGGACGCCCTGAATCTGGCACAGATGCAGGAGCAGA
3
ATAD3B CGCTGCAGTTGGAGCAACAGTCCAAGCTCAAAGAGTATGAGGCCGCCGTGGAGCAGCTCA
ATAD3A CGCTGCAGTTGGAGCAACAGTCCAAGCTCAAAGAGTATGAGGCCGCCGTGGAGCAGCTCA
S5_ATAD3A CGCTGCAGTTGGAGCAACAGTCCAAGCTCAAAGAGTATGAGGCCGCCGTGGAGCAGCTCA
rs145736326
ATAD3B AGAGCGAGCAGATCCGGGCGCAGGCTGAGGAGAGGAGGAAGACCCTGAGCGAGGAGACCC
ATAD3A AGAGCGAGCAGATCCGGGCGCAGGCTGAGGAGAGGAGGAAGACCCTGAGCGAGGAGACCC
S5_ATAD3A AGAGNGAGCAGATCCGGGCGCAGGCTGAGGAGAGGAGGAAGACCCTGAGCGAGGAGACCC
4
ATAD3B GGCAGCACCAGGCCAGGGCCCAGTATCAAGACAAGCTGGCCCGGCAGCGCTACGAGGACC
ATAD3A GGCAGCACCAGGCCAGGGCCCAGTATCAAGACAAGCTGGCCCGGCAGCGCTACGAGGACC
S5_ATAD3A GGCAGCACCAGGCCAGGGCCCAGTATCAAGACAAGCTGGCCCGGCAGCGCTACGAGGACC
5
ATAD3B AACTGAAGCAGCAGCAACTTCTCAATGAGGAGAATTTACGGAAGCAGGAGGAGTCCGTGC
ATAD3A AACTGAAGCAGCAGCAACTTCTCAATGAGGAGAATTTACGGAAGCAGGAGGAGTCCGTGC
S5_ATAD3A AACTGAAGCAGCAGCAACTTCTCAATGAGGAGAATTTACGGAAGCAGGAGGAGTCCGTGC
6
ATAD3B AGAAGCAGGAAGCCATGCGGCGAGCCACCGTGGAGCGGGAGATGGAGCTGCGGCACAAGA
ATAD3A AGAAGCAGGAAGCCATGCGGCGAGCCACCGTGGAGCGGGAGATGGAGCTGCGGCACAAGA
S5_ATAD3A AGAAGCAGGAAGCCATGCGGCGAGCCACCGTGGAGCGGGAGATGGAGCTGCGGCACAAGA
ATAD3B ATGAGATGCTGCGAGTGGAGACCGAGGCCCGGGCGCGCGCCAAGGCCGAGCGGGAGAATG
ATAD3A ATGAGATGCTGCGAGTGGAGGCCGAGGCCCGGGCGCGCGCCAAGGCCGAGCGGGAGAATG
S5_ATAD3A ATGAGATGCTGCGAGTGGAGGCCGAGGCCCGGGCGCGCGCCAAGGCCGAGCGGGAGAATG
ATAD3B CAGACATCATCCGCGAGCAGATCCGCCTGAAGGCGACCGAGCACCGTCAGACCGTCTTGG
ATAD3A CAGACATCATCCGCGAGCAGATCCGCCTGAAGGCGGCCGAGCACCGTCAGACCGTCTTGG
S5_ATAD3A CAGACATCATCCGCGAGCAGATCCGCCTGAAGGCGGCCGAGCACCGTCAGACCGTCTTGG
7
ATAD3B AGTCCATCAGGACGGCTGGCACCTTGTTTGGGGAAGGATTCCGTGCCTTTGTGACAGACC
ATAD3A AGTCCATCAGGACGGCTGGCACCTTGTTTGGGGAAGGATTCCGTGCCTTTGTGACAGACT
S5_ATAD3A AGTCCATCAGGACGGCTGGCACCTTGTTTGGGGAAGGATTCCGTGCCTTTGTGACAGACT
8
rs1135025
ATAD3B GGGACAAAGTGACAGCCACGGTGGCTGGGCTGACGCTGCTGGCTGTCGGGGTCTACTCAG
ATAD3A GGGACAAAGTGACAGCCACGGTGGCTGGGCTGACGCTGCTGGCTGTTGGGGTCTACTCAG
S5_ATAD3A GGGACAAAGTGACAGCCACGGTGGCTGGGCTGACGCTGCTGGCTGTNGGGGTCTACTCAG
rs765184665
rs145958395
rs141479773
rs756874226
ATAD3B CCAAGAATGCGACAGCCGTCACTGGCCGCTTCATCGAGGCTCGGCTGGGGAAGCCGTCCC
ATAD3A CCAAGAATGCCACGCTTGTCGCCGGCCGCTTCATCGAGGCTCGGCTGGGGAAGCCGTCCC
S5_ATAD3A CCAAGAATGCNACNNNNGTCNCNGGCCGCTTCATCGAGGCTCGGCTGGGGAAGCCGTCCC
rs28675990
9
ATAD3B TAGTGAGGGAGACGTCCCGCATCACGGTGCTGGAGGCGCTGCGGCACCCCATCCAGGTCA
ATAD3A TAGTGAGGGAGACGTCCCGCATCACGGTGCTTGAGGCGCTGCGGCACCCCATCCAGGTCA
S5_ATAD3A TAGTGAGGGAGACGTCCCGCATCACGGTGCTNGAGGCGCTGCGGCACCCCATCCAGGTCA
rs113107431
rs112145664
10
ATAD3B GCCGGCGGCTCCTCAGTCGACCCCAGGACGTGCTGGAGGGTGTTGTGCTTACTCCAGCC
ATAD3A GCCGGCGGCTCCTCAGTCGACCCCAGGACGCGCTGGAGGGTGTTGTGCTCACTCCAGCC
S5_ATAD3A GCCGGCGGCTCCTCAGTCGANCCCAGGACGCGCTGGAGGGTGTTGTGCTNACTCCAGCC
ATAD3B TGGAAGCACGGGTGCGCGACATCGCCATAGCAACCAGGAACACCAAGAAGAACCGGGGCC
ATAD3A TGGAAGCACGGGTGCGCGACATCGCCATAGCAACAAGGAACACCAAGAAGAACCGCAGCC
S5_ATAD3A TGGAAGCACGGGTGCGCGACATCGCCATAGCAACAAGGAACACCAAGAAGAACCGCAGCC
11
ATAD3B TGTACAGGCACATCCTGCTGTATGGGCCACCAGGCACCGGGAAGACGCTGTTTGCCAAGA
ATAD3A TGTACAGGAACATCCTGATGTACGGGCCACCAGGCACCGGGAAGACGCTGTTTGCCAAGA
S5_ATAD3A TGTACAGGAACATCCTGATGTACGGGCCACCAGGCACCGGGAAGACGCTGTTTGCCAAGA
ATAD3B AACTCGCCCTGCACTCAGGCATGGACTACGCCATCATGACAGGCGGGGACGTGGCCCCCA
ATAD3A AACTCGCCCTGCACTCAGGCATGGACTACGCCATCATGACAGGCGGGGACGTGGCCCCCA
S5_ATAD3A AACTCGCCCTGCACTCAGGCATGGACTACGCCATCATGACAGGCGGGGACGTGGCCCCCA
ATAD3B TGGGGCGGGAAGGCGTGACCGCCATGCACAAGCTCTTTGACTGGGCCAATACCAGCCGGC
ATAD3A TGGGGCGGGAAGGCGTGACCGCCATGCACAAGCTCTTTGACTGGGCCAATACCAGCCGGC
S5_ATAD3A TGGGGCGGGAAGGCGTGACCGCCATGCACAAGCTCTTTGACTGGGCCAATACCAGCCGGC

```
                   12                                                   13
ATAD3B          GCGGCCTCCTGCTCTTCATGGATGAAGCAGACGCCTTCCTTCGGAAGCGAGCCACTGAGG
ATAD3A          GCGGCCTCCTGCTCTTTGTGGATGAAGCGGACGCCTTCCTTCGGAAGCGAGCCACCGAGA
S5_ATAD3A       GCGGCCTCCTGCTCTTTGTGGATGAAGCGGACGCCTTCCTTCGGAAGCGAGCCACCGAGA
                ****************  ********** ************************** *** 

ATAD3B          AGATAAGCAAGGACCTCAGAGCCACACTGAACGCCTTCCTGTACCACATGGGCCAACACA
ATAD3A          AGATAAGCGAGGACCTCAGGGCCACACTGAACGCCTTCCTGTACCGCACGGGCCAGCACA
S5_ATAD3A       AGATAAGCGAGGACCTCAGGGCCACACTGAACGCCTTCCTGTACCGCACGGGCCAGCACA
                ******** ********** ************************* ** ****** ****

                      14
ATAD3B          GCAACAAATTCATGCTGGTCCTGGCCAGCAATCTGCCTGAGCAGTTCGACTGTGCCATCA
ATAD3A          GCAACAAGTTCATGCTGGTCCTGGCCAGCAACCAACCAGAGCAGTTCGACTGGGCCATCA
S5_ATAD3A       GCAACAAGTTCATGCTGGTCCTGGCCAGCAACCAACCAGAGCAGTTCGACTGGGCCATCA
                ******* *********************** *  ** ************** *******

ATAD3B          ACAGCCGCATTGACGTGATGGTCCACTTCGACCTGCCGCAGCAGGAGGAGCGGGAGCGCC
ATAD3A          ATGACCGCATCAATGAGATGGTCCACTTCGACCTGCCAGGGCAGGAGGAACGGGAGCGCC
S5_ATAD3A       ATGACCGCATCAATGAGATGGTCCACTTCGACCTGCCAGGGCAGGAGGAACGGGAGCGCC
                *   ******  * * *********************   ********* **********

                                                                      15
ATAD3B          TGGTGAGACTGCATTTTGACAACTGTGTTCTTAAGCCGGCCACAGAAGGAAAACGGCGCC
ATAD3A          TGGTGAGAATGTATTTTGACAAGTATGTTCTTAAGCCGGCCACAGAAGGAAAGCAGCGCC
S5_ATAD3A       TGGTGAGAATGTATTTTGACAAGTATGTTCTTAAGCCGGCCACAGAAGGAAAGCAGCGCC
                ******** ** ********** * *************************** * *****

ATAD3B          TGAAGCTGGCCCAGTTTGACTACGGGAGGAAGTGCTCGGAGGTCGCTCGGCTGACGGAGG
ATAD3A          TGAAGCTGGCCCAGTTTGACTACGGGAGGAAGTGCTCGGAGGTCGCTCGGCTGACGGAGG
S5_ATAD3A       TGAAGCTGGCCCAGTTTGACTACGGGAGGAAGTGCTCGGAGGTCGCTCGGCTGACGGAGG
                ************************************************************

                                                            16
ATAD3B          GCATGTCGGGCCGGGAGATCGCTCAGCTGGCCGTGTCCTGGCAGGCCACGGCATATGCCT
ATAD3A          GCATGTCGGGCCGGGAGATCGCTCAGCTGGCCGTGTCCTGGCAGGCCACGGCGTATGCCT
S5_ATAD3A       GCATGTCGGGCCGGGAGATCGCTCAGCTGGCCGTGTCCTGGCAGGCCACGGCGTATGCCT
                **************************************************** *******

ATAD3B          CCAAGGACGGGGTCCTCACTGAGGCCATGATGGACGCCTGTGTGCAAGATGCTGTCCAGC
ATAD3A          CCGAGGACGGGGTCCTGACCGAGGCCATGATGGACACCCGCGTGCAAGATGCTGTCCAGC
S5_ATAD3A       CCGAGGACGGGGTCCTGACCGAGGCCATGATGGACACCCGCGTGCAAGATGCTGTCCAGC
                ** ************* ** *************** ** * *******************

ATAD3B          AGTACCGACAGAAGATGCGCTGGCTGAAGGCGGAGGGGCCTGGGCGCGGGGTCGAGCACC
ATAD3A          AGCACCAGCAGAAGATGTGCTGGCTGAAGGCGGAAGGGCCTGGGCGTGGGGACGAGCCCT
S5_ATAD3A       AGCACCAGCAGAAGATGTGCTGGCTGAAGGCGGAAGGGCCTGGGCGTGGGGACGAGCCCT
                ** ***  ********* **************** *********** **** ***** * 

ATAD3B          CCCTATCCGGAGTCCAAGGCGAGACCCTCACCTCATGGAGCCTGGCCACGGACCCCTCCT
ATAD3A          CCCCATCCTGAGTCCACAGGGAGATCCACAGCTCACGGAGCCTGGCCGCGGACCCCTCCC
S5_ATAD3A       CCCCATCCTGAGTCCACAGGGAGATCCACANCTCACNGAGCCTGGCCGCGGACCCCTCCC
                *** **** *******  * **** ** ** ****  ********** *********** 

ATAD3B          ACCCCTGCCTTGCCGGCCCCTGCACATTTAGGATATGCTCCTGG[illegible]TGGGGACTGGGCTGT
ATAD3A          ACCCCTGCCTTGCCGGCCCCTGCACATTTAGGATATGCTCCTGGGTGGGGACTGGGCTGT
S5_ATAD3A       ACCCCTGCCTTGCCGGCCCCTGCACATTTAGGATATGCTCCTGGGTGGGGACTGGGCTGT
                ******************************************** ***************

ATAD3B          GCCCAGGGCCTCTGTCCCCCAGGATGTCTTGTGGTGGCGGTCGGCCGTTCTGCCCCCCAG
ATAD3A          GCCCAGGGCCTCTGTCCCCCAGGATGTCTTGTGGTGCGGGTCGGCCGTTCTGCCCCCCAG
S5_ATAD3A       GCCCAGGGCCTCTGTCCCCCAGGATGTCTTGTGGTGNNGGTCGGCCGTTCTGCCCCCCAG
                ************************************  **********************

ATAD3B          GGCACCCCCTGTTGTAGGCACTGGCTAGGGAGGGGCAGGCCTCCTTCCTGCCCCTCGAGA
ATAD3A          GGCACCCCCTGTTGTAGGCACTGGCTAGGGAGGGGCAGGCCTCCTTCCTGCCCCTCGAGA
S5_ATAD3A       GGCACCCCCTGTTGTAGGCACTGGCTAGGGAGGGGCAGGCCTCCTTCCTGCCCCTCGAGA
                ************************************************************

ATAD3B          CACTCTTGGGAGATGCATTTTCCGTCTGGCTCACAGGGGGAGGGTGAGGCTTTG[illegible]ACCCC
ATAD3A          CACTCTTGGGAGATGCATTTTCCGTCTGGCTCACAGGGGGAGGGTGAGGCTTTGCACCCC
S5_ATAD3A       CACTCTTGGGAGANGCATTTTCCGTCTGGCTCACAGGGGGAGGGTGAGGCTTTGCACCCC
                ************* **************************************** *****

ATAD3B          AGCCCCTGCCCAGGCCACTGTGAGGGTGGGTGCTGGCTGAGCCCCTGGGGCAGAAGGAGT
ATAD3A          AGCCCCTGCCCAGGCCACTGTGAGGGTGGGTGCTGGCTGAGCCCCCGGGGCAGCAGGAGC
S5_ATAD3A       AGCCCCTGCCCAGGCCACTGTGAGGGTGGGTGCTGGCTGAGCCCCCGGGGCAGCAGGAGC
                ********************************************* ******* ***** 

ATAD3B          GGGGCAGGCGGGGTCTTTGTTCTCGGCTCCCACAGCAGAGCCAGGTGAGGGGGGGCCTGC
ATAD3A          CAGGCAGGTGATGTCTTTGTTCTCGGCTCCCACAGCAGAGCCAGGTGAGGGGGCGCCTGC
S5_ATAD3A       CAGGCAGGTGATGTCTTTGTTCTCGGCTCCCACAGCAGAGCCAGGTGAGGGGGCGCCTGC
                  ****** *  ***************************************** ******

ATAD3B          CAGGACTAGACAGAAGTGGGGCGGCCTGAACCCTGCTTCCAGCCATGGCCAGGGGCCAC-
ATAD3A          CAGGGCCAGACCCAGGTGGGGCAGCCTGAACCCTGCTTCCCCCTGTGGCCGGCATGCCCC
S5_ATAD3A       CAGGGCCAGACCCAGGTGGGGCAGCCTGAACCCTGCTTCCCCCTGTGGCCGGCATGCCC-
                **** * ****  * ******* *****************  *  ***** *    * * 

ATAD3B          -----------------------------------------GGAACCCGGCAGGGGTGTC
ATAD3A          GATCTTTCACACACTGGTGACCCTGAGAGAGGAGGGAGGAGGGAACCTGGCGGGGGTGTC
S5_ATAD3A       ------------------------------------------------------------

ATAD3B          TGAGGCCGCCCTGTCAGCTGGCCGGTCCAAGCCTGTGGCTGGAGCTGGTGTGTGTTTATC
ATAD3A          TGAGGCCGCACTGTCAGCTGGCCGGTCCAAGCCTGTGGCTGGAGCTGGGGTCTGTTTACC
S5_ATAD3A       ------------------------------------------------------------

ATAD3B          TAATAAAGTCCCACAGGTGCCTCACCAAAAAAAAAAAAAAAAAAAAAAAA
ATAD3A          TAATAAAGTCCCACAGGTGCCTCATTAAAAAAAAAA--------------
S5_ATAD3A       --------------------------------------------------
```

D

SNP	Minor Allele Frequency (MAF)
rs144868573	T= 0.00006
rs747600018	C=0.00003
rs2478794	G=0.0003
rs747464654	G=0.0008
rs763031171	A=0.0044
rs140940347	G=0.0050
rs2767471	T=0.0018
rs3979437	T=0.0002
rs2767468	C=0.0002
rs771532394	T=0.00008
rs1135025	C=0.0154
rs765184665	G=0.00005
rs757691033	C=0.00004
rs145958395	A=0.00008
rs781603814	T=0.00001
rs141479773	A=0.00003
rs748131185	C=0.00001
rs756874226	T=0.00003
rs28675990	T=0.059
rs1781156	G=0.003
rs746324717	C=0.001
rs142299242	T=0.00005
rs774408088	C=0.00006
rs112145664	T=0.0062
rs819976	A=0.2476
rs860213	A=0.2149
rs530396032	T=0.00012
rs148709813	A=0.00061
rs819977	G=0.2539
rs819980	C=0.2546
rs145736326	T=0.0152
rs113107431	T=0.00073

E

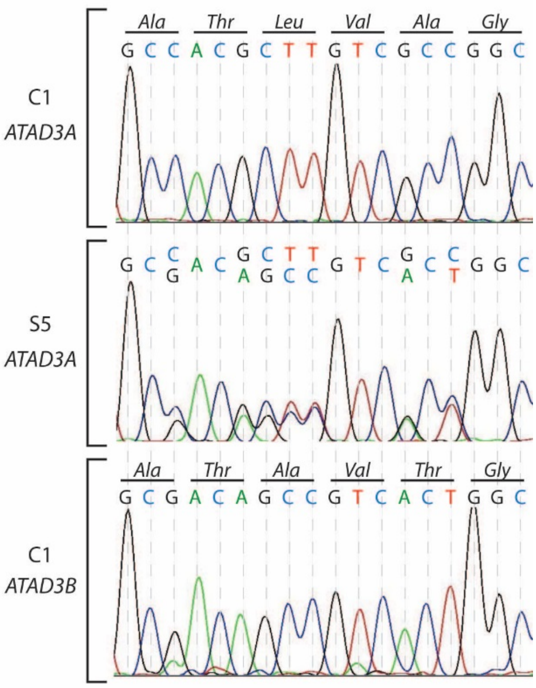

F

```
ATAD3B          MSWLFGVNKGPKGEGAGPPPPLPPAQPGAEGGGDRGLGDRPAPKDKWSNFDPTGLERAAK
ATAD3A          MSWLFGINKGPKGEGAGPPPPLPPAQPGAEGGGDRGLGDRPAPKDKWSNFDPTGLERAAK
Fusion          MSWLFGVNKGPKGEGAGPPPPLPPAQPGAEGGGDRGLGDRPAPKDKWSNFDPTGLERAAK
                ******:*****************************************************
                         2                         3
ATAD3B          AARELEHSRYAKEALNLAQMQEQTLQLEQQSKLKEYEAAVEQLKSEQIRAQAEERRKTLS
ATAD3A          AARELEHSRYAKDALNLAQMQEQTLQLEQQSKLKEYEAAVEQLKSEQIRAQAEERRKTLS
Fusion          AARELEHSRYAKEALNLAQMQEQTLQLEQQSKLKEYEAAVEQLKSEQIRAQAEERRKTLS
                ************:***********************************************
                         4                  5                      6
ATAD3B          EETRQHQARAQYQDKLARQRYEDQLKQQQLLNEENLRKQEESVQKQEAMRRATVEREMEL
ATAD3A          EETRQHQARAQYQDKLARQRYEDQLKQQQLLNEENLRKQEESVQKQEAMRRATVEREMEL
Fusion          EETRQHQARAQYQDKLARQRYEDQLKQQQLLNEENLRKQEESVQKQEAMRRATVEREMEL
                ************************************************************
                                                              7
ATAD3B          RHKNEMLRVETEARARAKAERENADIIREQIRLKASEHRQTVLESIRTAGTLFGEGFRAF
ATAD3A          RHKNEMLRVEAEARARAKAERENADIIREQIRLKAAEHRQTVLESIRTAGTLFGEGFRAF
Fusion          RHKNEMLRVEAEARARAKAERENADIIREQIRLKAAEHRQTVLESIRTAGTLFGEGFRAF
                **********:************************:************************
                           8
ATAD3B          VTDRDKVTATVAGLTLLAVGVYSAKNATAVTGRFIEARLGKPSLVRETSRITVLEALRHP
ATAD3A          VTDWDKVTATVAGLTLLAVGVYSAKNATLVAGRFIEARLGKPSLVRETSRITVLEALRHP
Fusion          VTDWDKVTATVAGLTLLAVGVYSAKNATLVAGRFIEARLGKPSLVRETSRITVLEALRHP
                *** ************************ *:*****************************
                  9                  10
ATAD3B          IQVSRRLLSRPQDVLEGVVLSPSLEARVRDIAIATRNTKKNRGLYRHILLYGPPGTGKTL
ATAD3A          IQVSRRLLSRPQDALEGVVLSPSLEARVRDIAIATRNTKKNRSLYRNILMYGPPGTGKTL
Fusion          IQVSRRLLSRPQDALEGVVLSPSLEARVRDIAIATRNTKKNRSLYRNILMYGPPGTGKTL
```

```
                *************.****************************.***.**:**********
                   11                                       12
ATAD3B          FAKKLALHSGMDYAIMTGGDVAPMGREGVTAMHKLFDWANTSRRGLLLFMDEADAFLRKR
ATAD3A          FAKKLALHSGMDYAIMTGGDVAPMGREGVTAMHKLFDWANTSRRGLLLFVDEADAFLRKR
Fusion          FAKKLALHSGMDYAIMTGGDVAPMGREGVTAMHKLFDWANTSRRGLLLFVDEADAFLRKR
                *************************************************:**********
                  13                       14
ATAD3B          ATEEISKDLRATLNAFLYHMGQHSNKFMLVLASNLPEQFDCAINSRIDVMVHFDLPQQEE
ATAD3A          ATEKISEDLRATLNAFLYRTGQHSNKFMLVLASNQPEQFDWAINDRINEMVHFDLPGQEE
Fusion          ATEKISEDLRATLNAFLYRTGQHSNKFMLVLASNQPEQFDWAINDRINEMVHFDLPGQEE
                ***:**:***********: ************** ***** ***.**: ******* ***
                                     15                                    16
ATAD3B          RERLVRLHFDNCVLKPATEGKRRLKLAQFDYGRKCSEVARLTEGMSGREIAQLAVSWQAT
ATAD3A          RERLVRMYFDKYVLKPATEGKQRLKLAQFDYGRKCSEVARLTEGMSGREIAQLAVSWQAT
Fusion          RERLVRMYFDKYVLKPATEGKQRLKLAQFDYGRKCSEVARLTEGMSGREIAQLAVSWQAT
                ******::**: *********:**************************************

ATAD3B          AYASKDGVLTEAMMDACVQDAVQQYRQKMRWLKAEGPGRGVEHPLSGVQGETLTSWSLAT
ATAD3A          AYASEDGVLTEAMMDTRVQDAVQQHQQKMCWLKAEGPGRGDEPSPS--------------
Fusion          AYASEDGVLTEAMMDTRVQDAVQQHQQKMCWLKAEGPGRGDEPSPS--------------
                ****:**********: *******::*** ********** *   *

ATAD3B          DPSYPCLAGPCTFRICSWMGTGLCPGPLSPRMSCGGGRPFCPPGHPLL
ATAD3A          ------------------------------------------------
Fusion          ------------------------------------------------
```

Figure S5. *ATAD3B* and *ATAD3A* mRNA and predicted protein sequences. (A) cDNA was generated from mRNA isolated from S1a, S3 and S4 fibroblasts, and primers OT441 and OT443 were used to amplify the *ATAD3B/A* fusion. The amplicons were subjected to Sanger sequencing and aligned with ATAD3B isoform 1 (NM_031921.5) and ATAD3A isoform 2 (NM_001170535.1) using the multiple sequence alignment tool MUSCLE (http://www.ebi.ac.uk/Tools/msa/muscle/). Primer OT441 is indicated in bold italic. Bases specific to *ATAD3B* are highlighted blue and specific to *ATAD3A* are highlighted in yellow. For all bases specific to *ATAD3B* or *ATAD3A*, SNPs are in red and dbSNP numbers indicated (http://www.ncbi.nlm.nih.gov/SNP/). Exon boundaries are boxed and numbered. Exons located within the *ATAD3* deletion breakpoint regions that cannot be further refined are indicated by underline (S1a and S4 paternal) and gray boxes (S3 and S4 maternal). Sanger sequencing of exons 10-16 in the *ATAD3B/A* fusion cDNA for all subjects showed no changes from the reference sequence. **(B** and **C)** cDNA was generated from mRNA isolated from S5. Primers OT441 and OT445 were used to amplify *ATAD3B* (**B**), while OT441 and OT443 were used to amplify *ATAD3A* (**C**) and the amplicon was analysed as in **A**, with relevant coding SNPs indicated in red. Stop codons are underlined and 3' UTRs for *ATAD3B* and *ATAD3A* indicated in italics. Of note are several heterozygous bases detected in *ATAD3A* exon 8 from S5 that are not noted as SNPs in the databases. **(D)** The minor allele frequency (MAF) for the relevant SNPs sourced from ExAC (http://exac.broadinstitute.org/) or gnomAD (http://gnomad.broadinstitute.org/). **(E)** Sanger sequencing traces of the region in *ATAD3A* exon 8 that showed multiple heterozygous bases in S5, aligned with traces from control (C1) *ATAD3B* and *ATAD3A* cDNA. All heterozygous bases correspond to either the *ATAD3B* or *ATAD3A* reference sequence, indicating that one *ATAD3A* allele in S5 contains a region of *ATAD3B* sequence, resulting in two missense variants, p.L269A and p.A271T, in the ATAD3A isoform 2 protein sequence. **(F)** Based on cDNA sequencing and the identified deletion breakpoints, an identical ATAD3B/A fusion protein was predicted for S1a/S1b, S2,

S3 and S4. Alignment of this fusion protein to ATAD3B variant 1 (NP_114127) and ATAD3A variant 2 (NP_001164006) using Clustal Omega (http://www.ebi.ac.uk/Tools/msa/clustalo/) indicates the predicted protein is identical to ATAD3A variant 2, with the exception of p.I7V and p.D73E (highlighted). Amino acids located at the exon boundaries are indicated in bold, with corresponding exons numbered. The deletion breakpoints are predicted to occur in proximity to exon 5 for S1a/S1b and S4 (paternal), and to exon 3 and 4 for S2, S3 and S4 (maternal).

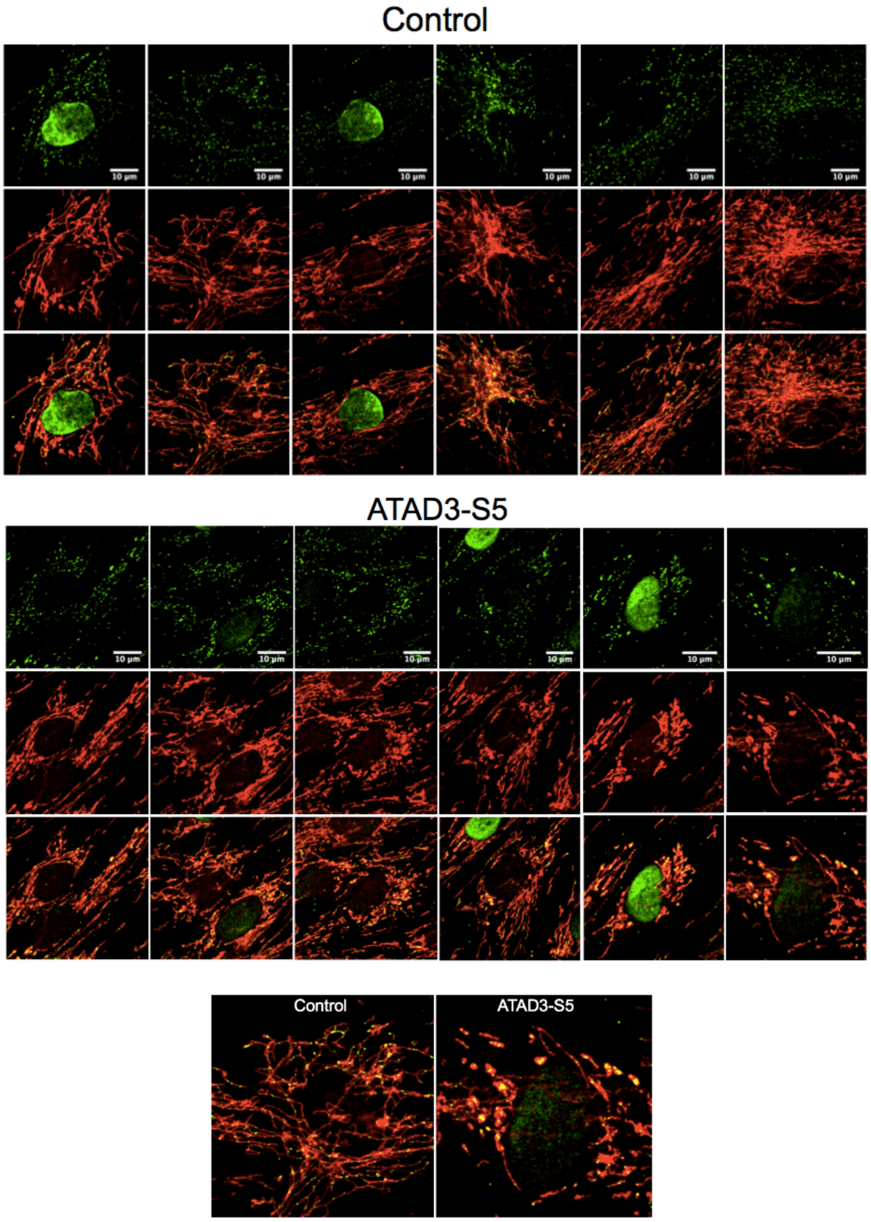

Figure S6. ATAD3 deficiency is associated with mtDNA abnormalities. Representative images of anti-DNA labeled cells imaged by confocal microscopy for C5 and S5.

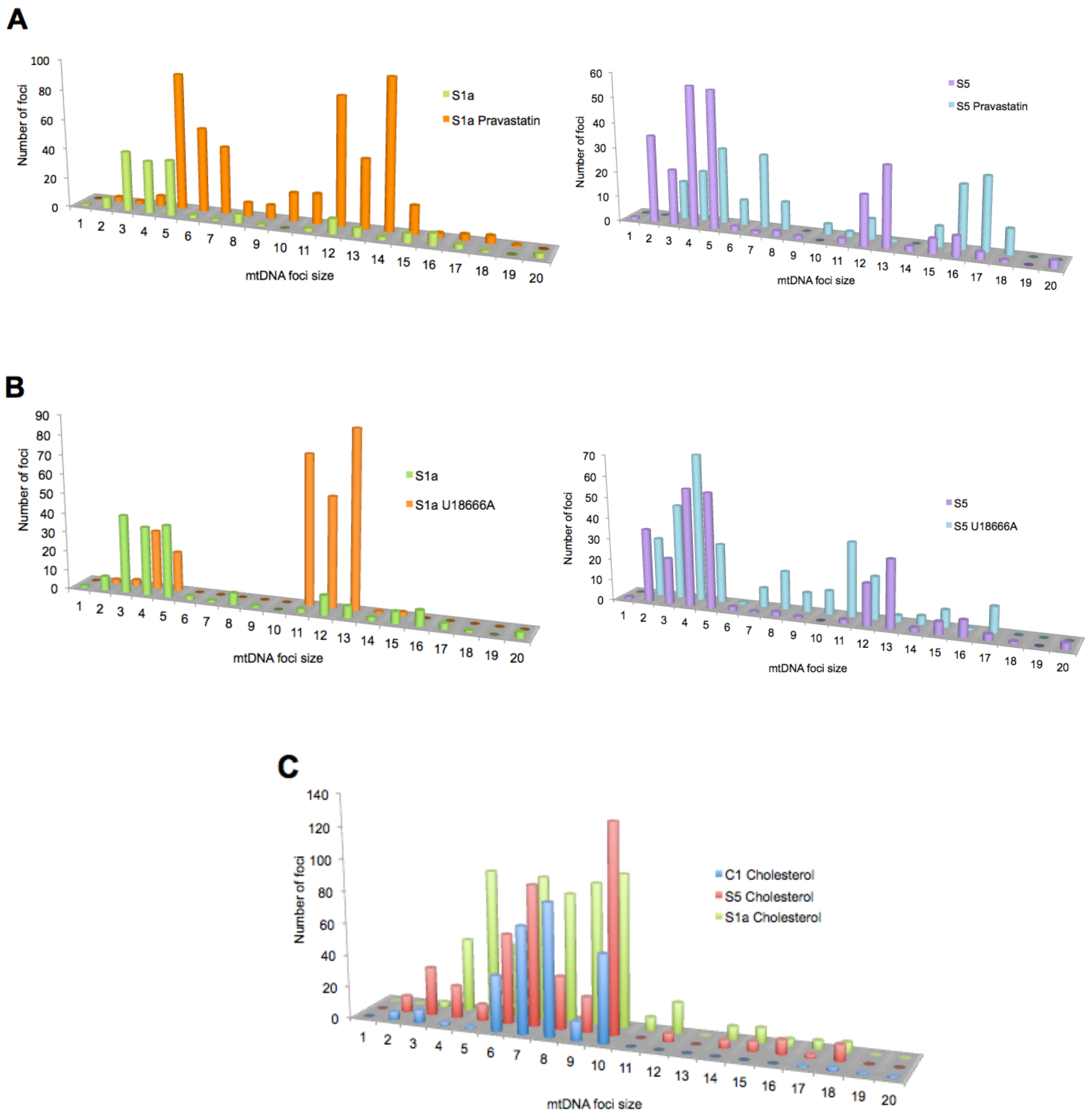

Figure S7 Pravastatin or U186666A treatments exacerbate the mtDNA disorganization phenotype of ATAD3 mutant fibroblasts. Mitochondrial DNA foci were detected by immune-fluorescence in fibroblasts of subjects S1a and S5 after treating cells with **(A)** pravastatin or **(B)** U18666A. **(C)** Mitochondrial DNA foci size in S1a, S5 and Control (C5) cells treated with 5 mM cholesterol (data for C5 are reproduced from Fig. 5C).

A

#	Network	p Value	FDR	Ratio	p Value	FDR	Ratio
1	Cholesterol Biosynthesis	7.84E-07	0.000573	17/103	2E-06	0.000751	18/103
2	Transcription_Sirtuin6 regulation and functions	0.000241	0.022021	10/64	1.8E-06	0.000751	14/64
3	Signal transduction_Cyclic AMP signaling	0.000119	0.014555	8/38	9.4E-06	0.002342	10/38
4	Adiponectin in pathogenesis of type 2 diabetes	1.45E-05	0.005291	8/29	0.00235	0.083935	6/29
5	Protein folding and maturation_Angiotensin system maturation	4.71E-05	0.010437	9/43	3.1E-05	0.00572	10/43

B

Network Objects S5 vs C1<br>FDR 5.73e-4, p-value 7.84e-7, Ratio 17/103		
1	CYP51A1	1.817
2	DHC24	1.671
3	DHCR7	1.468
4	EBP	1.251
5	ER24	1.190
6	ERG1	2.117
7	FDFT1	1.378
8	FDPS	1.601
9	HMDH	2.118
10	HMGCS1	2.390
11	HSD17B7	1.782
12	IDI1	1.971
13	LSS	1.256
14	MVD	1.640
15	MVK	1.255
16	SC4MOL	1.256
17	SC5D	1.608

Network Objects S5 (+ps) vs C1 (+ps)<br>FDR 7.51e-4, p-value 2.01e-6, Ratio 18/103		
1	CYP51A1	1.701
2	DHC24	1.731
3	DHCR7	1.875
4	EBP	2.074
5	ER24	2.600
6	ERG1	1.852
7	FDFT1	1.720
8	FDPS	2.458
9	HMDH	1.738
10	HMGCS1	2.264
11	HSD17B7	2.341
12	IDI1	2.198
13	LSS	1.315
14	MVD	2.494
15	MVK	1.782
16	NSDHL	1.265
17	SC4MOL	1.548
18	SC5D	1.178

Figure S8. The most up-regulated metabolic pathway in cells of subject S5 is cholesterol biosynthesis based on Meta-Core^{TM} analysis of four independent replicates. (A) The five networks (pathways) most enriched in subject S5 cells compared to a control. **(B)** Individual factors for cholesterol biosynthesis S5 vs. control. In total 17 factors involved in cholesterol biosynthesis displayed higher expression in S5 vs the control, producing an overall FDR of 0.0006. Because only 1 sample passed QC for S5, we also compared three replicates for S5 treated with pravastatin (ps) with 3 control replicates treated with pravastatin (light gray in **(A)** and **(B)**). The effects of pravastatin are expected to cancel out, more especially as the drug alone had almost no appreciable effect on the gene expression profile of the control cells. Therefore, it was to be expected that cholesterol biosynthesis (and other parameters) would show similar differences in S5 vs. control comparisons irrespective of pravastatin exposure and this proved to be the case (panels **A** and **B**).

A

#	Network	p Value	FDR	Ratio	p Value	FDR	Ratio
1	Steroid metabolism_Cholesterol biosynthesis	1.2E-07	1.36E-05	17/88	3.67E-06	0.000114	17/88
2	1,2-dioleoyl-sn-glycerol_3-phosphate pathway	2.15E-06	0.000114	12/94	2.15E-06	0.000114	18/94
3	1-linoleoyl-glycerol_3-phosphate pathway	2.96E-06	0.000114	12/96	2.96E-06	0.000114	18/96
4	1,2-didocosahexaenoyl-sn-glycerol_3-phosphate p/w	4.04E-06	0.000114	15/98	4.04E-06	0.000114	18/98
5	Phosphatidylcholine pathway	5.39E-06	0.000122	17/130	5.39E-06	0.000122	21/130
6	1,2-didocosapentaenoyl-sn-glycerol_3-phosphate p/w	2.5E-05	0.000465	13/91	2.5E-05	0.000465	16/91
7	Phosphatidylinositol-4,5-diphosphate pathway	2.88E-05	0.000465	11/92	2.88E-05	0.000465	16/92
8	1-docosahexaenoyl-glycerol_3-phosphocholine p/w	0.00033	0.007459	15/128	0.088646	0.385268	11/128
9	Phosphatidylinositol-3,4,5-triphosphate pathway	0.000437	0.008225	10/66	0.00076	0.01073	11/66
10	1-oleoyl-sn-glycero-3-phosphocholine pathway	0.001724	0.019847	13/120	0.061639	0.290219	11/120
11	D-glucuronic acid pathway	0.001793	0.022509	6/73	0.001793	0.022509	11/73
12	N-acyl-sphingosine phosphate pathway	0.002002	0.022623	11/108	0.002002	0.022623	14/108

B

#	Network	p Value	FDR	Ratio
1	N-acyl-sphingosine phosphate pathway	1.28E-05	1.29E-03	13/108
2	1,2-dioleoyl-sn-glycerol_3-phosphate pathway	3.76E-04	1.90E-02	10/94
3	1,2-didocosapentaenoyl-sn-glycerol_3-phosphate pathway	1.26E-03	3.44E-02	9/91
4	Phosphatidylcholine pathway	1.36E-03	3.44E-02	11/130
5	Sphingomyelin pathway	1.98E-03	3.58E-02	9/97
6	1,2-didocosahexaenoyl-sn-glycerol_3-phosphate pathway	2.13E-03	3.58E-02	9/98
7	Acyl-L-carnitine pathway	3.21E-03	4.63E-02	8/85
8	1-oleoyl-glycerol_3-phosphate pathway	4.28E-03	5.40E-02	10/129
9	1-linoleoyl-glycerol_3-phosphate pathway	6.76E-03	7.58E-02	8/96
10	2-oleoyl-glycerol_3-phosphate pathway	9.62E-03	9.72E-02	5/45

Figure S9. The most up-regulated endogenous metabolic networks are linked to lipid metabolism in S5 (A), and S1a (B) cells, based on MetaCore™ analysis. As in figure S8 light gray background indicates results for samples of subject S5 treated with pravastatin compared with 3 control replicates treated with pravastatin.

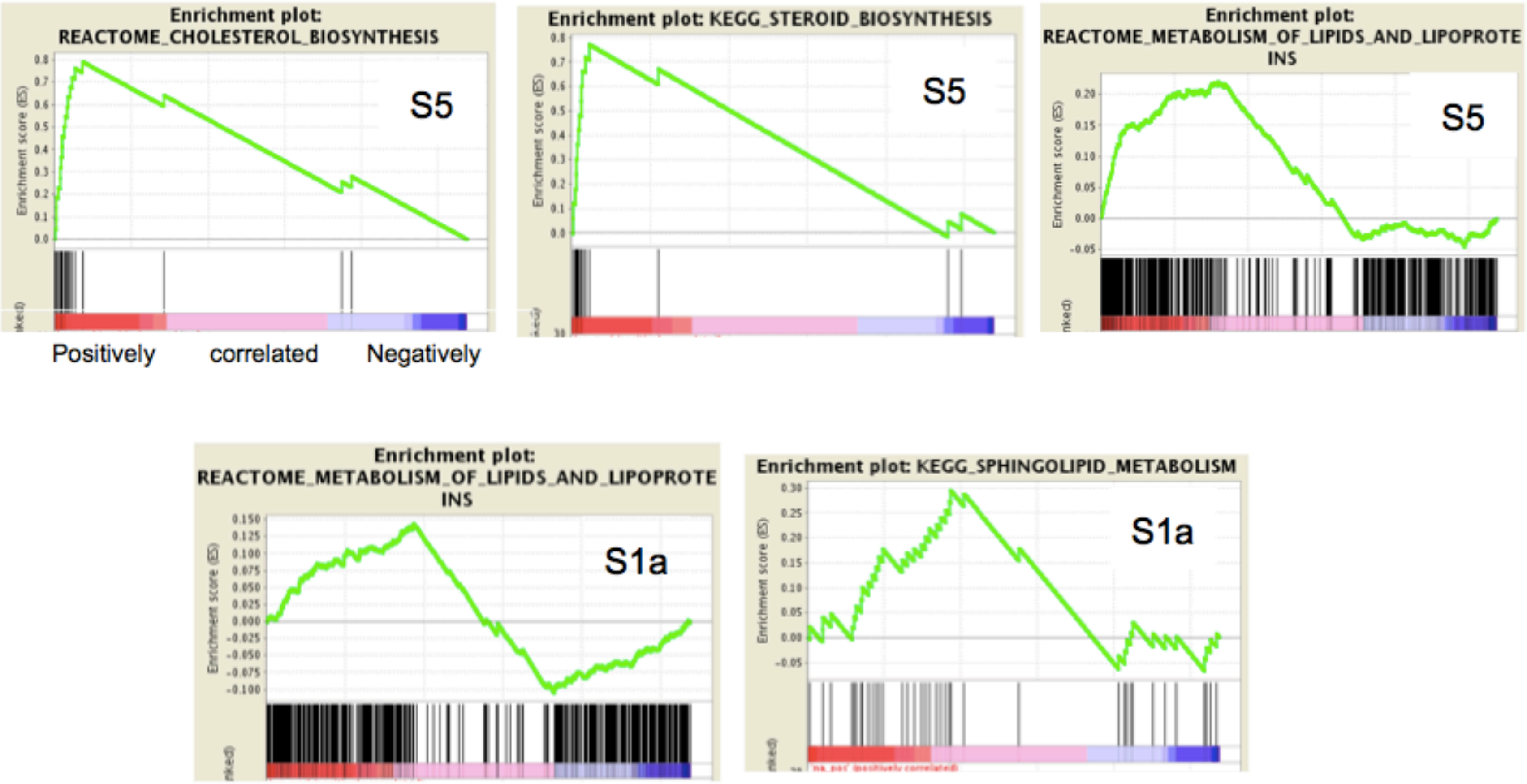

Figure S10. GSEA reveals elevated lipid metabolism and lipoproteins in the cells of subjects S5 and S1a. Selected Gene set enrichment profiles for lipid and cholesterol metabolism for S1a and S5 vs the control. Scores and rankings for these and other related gene sets were as follows. S1a: Reactome of lipids and lipoproteins - FDR q-value 0, p-value 0, Gene set ranking (GSR) 15; Reactome Phospholipid metabolism - FDR q-value 0, p-value 0.004, GSR126 KEGG sphingolipid metabolism - FDR q-value 0.002, p-value 0.007; GSR 133. S5: Reactome of lipids and lipoproteins - FDR q-value 0, p-value 0, GSR2; cholesterol biosynthesis - FDR q-value 0, p-value 0, GSR4; sterol biosynthesis - FDR q-value 0, p-value 0; GSR7; KEGG glycosphingolipid biosynthesis ganglio series - FDR q-value 0.002, p-value 0; GSR48.

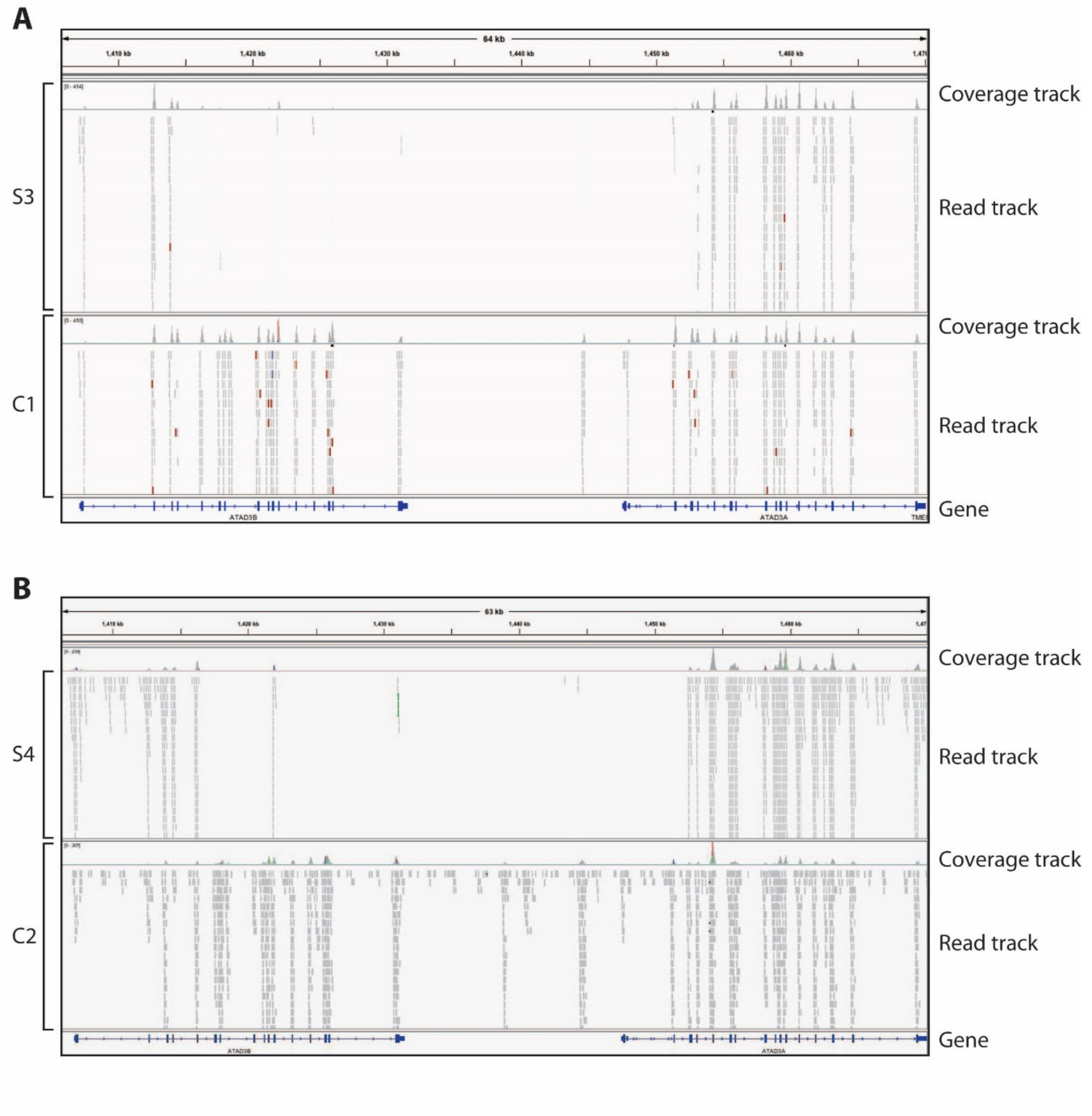

Figure S11. Whole exome sequencing of genomic DNA from S3 and S4. (A) Whole exome sequencing was performed using an Illumina Nextera Exome Capture kit and HiSeq4000 to an average depth of 100x in subject S3. An IGV screen grab (Integrated Genomics Viewer, Broad Institute [1; 2]) shows aligned reads from S3 and control (C1) to *ATAD3B* and *ATAD3A*. Open boxes indicate reads with ambiguous alignment. **(B)** Whole exome sequencing was performed using an Agilent SureSelect Human All Exon V5 kit and HiSeq2000 to an average depth of 100x in subject S4.

Table S1 Respiratory chain enzymes and mitochondrial DNA levels in subjects with *ATAD3* mutations

	Residual Enzyme Activity (% CS Ratio)								
**Muscle**	**S1a**	**S1b**	**S2**	**S3**	**(Ref Range)**	**S4**	**(Ref Range)**	**S5**	**(Ref Range)**
Complex I	57	35			36-269			113	67-144
Complex II	146	128			52-156			64	65-138
Complex III	46	45			62-185			115	69-146
Complex IV	65	52			36-192			99	66-144
Citrate Synthase	51[a]	71[a]			66-139[a]			63*	70-139[a]
**Liver**									
Complex I		67			65-137				
Complex II		51			59-127				
Complex III		41			77-127				
Complex IV		153			75-134				
Citrate Synthase		218[a]			93-111[a]				
**Skin Fibroblasts**									
Complex I	84			68	50-145	57	55-143		
Complex II	178			161	57-144	42	67-152		
Complex III	117			230	42-189	62	53-148		
Complex IV	107			99	44-170	58	66-180		
Citrate Synthase	108[a]			116	42-145[a]	109	36-163		
	**mtDNA:nDNA Ratio (as % of wild-type)**								
**Muscle**	9[b]	40			50-208				
**Liver**		107			78-119				
**Skin Fibroblasts - Proliferating**	169±5		100±1		100±19			74±11	100±19
**Skin Fibroblasts - Quiescent**	117±11				161±44			135±7	161±44

Activities of Complexes I to IV are expressed as % CS ratios, which represents % of the normal control mean value when expressed relative to citrate synthase. Reference ranges are observed normal ranges from pediatric controls for muscle (n=9), liver (n=8) and skin fibroblasts (n=35).

[a]Citrate synthase activities are expressed as % of the normal control mean value when expressed relative to protein.

q-PCR estimation of mitochondrial DNA:nuclear DNA ratio was performed as described, with observed reference ranges for 6 pediatric control muscle samples expressed relative to protein [3] or 6 paediatric control liver samples [4]. Data for fibroblast cell lines were obtained from proliferating and quiescent cells as described [5] and expressed as % of mean value for 2 proliferating control cell lines of 100%, ± SD. The copy number of mtDNA was approximately one-third lower than control average in subject S5 cells, and quiescent fibroblasts of subject S1a underwent a modest decrease in copy number, rather than the usual increase that accompanies the cessation of cell division.

[b] Skeletal muscle mtDNA levels increase in the first few months of life [6] and can be secondarily decreased in individuals with severe muscle disease [7], so the apparent decrease in mtDNA is likely exaggerated; however, this developmental change is unlikely to account for the 10-fold lower level of muscle mtDNA of subject S1a. On the other hand, the mtDNA copy number was normal in the liver of subject S1b (Table S2), the skeletal muscle and liver of subject S2 [8].

Mitochondrial DNA organization appears to be the feature of the organelle most susceptible to cholesterol perturbation. Among the OXPHOS enzymes, complex III was one of the lowest activities in muscle and liver of subjects S1a and S1b, and this could be another manifestation of cholesterol insufficiency, as statin-induced myopathy is associated with decreased complex III activity [9]. Therefore, subjects with mitochondrial diseases of unknown cause with a specific complex III deficiency might have defects in cholesterol metabolism. Clearly, these defects could result from, yet not be restricted to, defects in ATAD3.

Table S2. Deletions in *ATAD3* locus detected by Cyto-SNP array for subjects S1a, S1b, S2 and S3 and by CoreExome array for subject S5

Subject	HumanCytoSNP - 12 v2.1[a] or CoreExome-24 v1.1	Custom Array CGH
S1a	chr1:1,418,112-1,451,769 (min) (33,657 bp)<br><br>chr1:1,415,099-1,465,382 (max) (50,283 bp)[b]	chr1: 1,417,345-1,454,163 (min) (36,818 bp)<br>chr1: 1,416,476-1,455,796 (max) (39,320 bp)
S1b	chr1:1,418,112-1,451,769 (min) (33,657 bp)<br>chr1:1,415,099-1,465,382 (max) (50,283 bp)[c]	
S2	chr1:1,415,099-1,451,769 (min) (36,670 bp)<br>chr1:1,404,810-1,465,382 (max) (60,572 bp)[d]	chr1: 1,415,562-1,453,389 (min) (37,827 bp)<br>chr1: 1,414,611-1,454,138 (max) (39,527 bp)
S3	chr1:1,418,112-1,451,769 (min) (33,657 bp)<br>chr1:1,415,099-1,465,382 (max) (50,283 bp)[e]	
S5	chr1:1,387,747-1,431,163 (min) (43,416 bp)<br>chr1:1,386,089-1,447,325 (max) (61,236 bp)	

[a]LCSH >1Mbp were reported in subjects S1a, S1b, S2 and S3, consistent with 1st/2nd cousins.

[b]Located within a predicted 3.3 Mbp region of LCSH.

[c]Located within a predicted 7.6 Mbp region of LCSH.

[d]Located within a predicted 2.1 Mbp region of LCSH.

[e]Located within a predicted 1.2 Mbp region of LCSH.

Supplementary notes to Figure 7A

1. *SREBF2* was significantly increased (FDR <0.05) in expression in S5; however, there were no data for the micro RNA miR33A, located in an intron of *SREBF2* near the 3´ end of the gene, as the sequencing protocol was not designed to detect micro RNAs. Nevertheless, one of the targets of miR33A, ABC transporter A1, *ABCA1* [10], was 2 and 3 fold higher than controls in S1a and S5 cells (log_2FC 1.02 and 1.58), respectively, and the function of ABCA1 is to facilitate cholesterol trafficking from late endosome/lysosomes to the plasma membrane to facilitate efflux. Other ABC transporters with links to cholesterol efflux were also more highly expressed in the patient-derived cell lines than the control, again suggesting cholesterol export is elevated to avoid toxicity owing to increased biosynthesis. A marked exception to the up-regulation of ABCA transporters was *ABCA8*, which was among the 10 most down-regulated genes in subject S5 samples.

2. Elevated cholesterol and lipid production have been shown to be inversely correlated with insulin metabolism, and this was evident here not only in the decrease in Leptin (*LEP* log_2FC -6.00, S1a; -3.28, S5), but also other genes such as *BCHE*, *SEL1L2* and IGF1 (respectively, log_2 FC -5.49, -4.75 and -5.17, in S1a and -6.23, -4.72 and -4.98, in S5).

3. Altered expression of proposed partners of ATAD3. *CYP11A1* was strongly down-regulated in S5 (log_2FC -4.57). *TSPO* expression was significantly decreased (FDR < 0.05) in subjects' fibroblasts (log_2FC -1.01, S1a; -0.80, S5). *STARD9* (not illustrated) was significantly upregulated (log_2FC 2.13) in S5, but not in S1a (log_2FC 0.34). As its name indicates, STARD9 contains a steroidgenic acute regulatory protein-related lipid transfer (START) domain; and it belongs to the kinesin-3 family [11], whose members have roles in the transport of vesicles and organelles, including mitochondria [12]; thus STARD9 potentially ensures mitochondrial (DNA) distribution is accompanied by an appropriate lipid/steroid context. SPTLC, serine palmitoyltransferase is the key enzyme of sphingolipid biosynthesis, individual subunits were mostly up-regulated: log_2FC *SPTLC1* 0.81 (S5) and 0.39 (S1a), *SPTLC3* 0.86 (S5) and 1.09 (S1a), *SPTLC2* 0.62 (S1a), the exception was *SPTLC2* (log_2FC -0.16) in subject S5.

4. Although transcriptome analysis reported an up-regulation of factors involved in lipid metabolism in the ATAD3 deficient cells, there were suggestions that lipid metabolism in the mitochondria was down-regulated. Glycerol-3-Phosphate Acyltransferase 2 (GPAT2), which performs an essential step in mitochondrial glycerolipid biosynthesis, and Glycine-N-Acyltransferase (GLYAT), which has links to fatty acid metabolism in the mitochondria, were both significantly down-regulated in subjects S1a and S5 (Figure 7A), implying that the production of membrane lipids in the organelle is compromised as a consequence of ATAD3 deficiency.

5. PGC1α (PPARGC1A) is a major regulator of mitochondrial biogenesis [13] and an important factor in lipid metabolism and adipogenesis [14]. PGC1α expression was greatly up-regulated in the mutant ATAD3 cell lines (16- or 32-fold compared to the control cells, in which it was barely expressed). The GSEA suggests that the increase in PGC1α stimulates the expression of factors involved in lipid metabolism (Figure S10); notwithstanding this, it might also limit the extent of mitochondrial dysfunction associated with ATAD3 deficiency through compensatory mitochondrial biogenesis.

6. None of the factors known to engage directly in mtDNA replication was significantly altered in expression as a result of ATAD3 deficiency. There were some significant (FDR < 0.05) increases in topoisomerase expression: *TOP2A* log_2FC 1.34 (S1a); *TOP1MT* log_2FC 0.91 (S5); and *TOP2B* log_2FC 0.65 (S5) that might reflect topological changes contributing to the mtDNA organization phenotypes of the subjects.

Supplementary References

1. Robinson, J.T., Thorvaldsdottir, H., Winckler, W., Guttman, M., Lander, E.S., Getz, G., and Mesirov, J.P. (2011). Integrative genomics viewer. Nat Biotechnol 29, 24-26.
2. Thorvaldsdottir, H., Robinson, J.T., and Mesirov, J.P. (2013). Integrative Genomics Viewer (IGV): high-performance genomics data visualization and exploration. Brief Bioinform 14, 178-192.
3. Pagnamenta, A.T., Taanman, J.W., Wilson, C.J., Anderson, N.E., Marotta, R., Duncan, A.J., Bitner-Glindzicz, M., Taylor, R.W., Laskowski, A., Thorburn, D.R., et al. (2006). Dominant inheritance of premature ovarian failure associated with mutant mitochondrial DNA polymerase gamma. Hum Reprod 21, 2467-2473.
4. Wiltshire, E., Davidzon, G., Dimauro, S., Akman, H.O., Sadleir, L., Haas, L., Zuccollo, J., McEwen, A., and Thorburn, D.R. (2008). Juvenile alpers disease. Arch Neurol 65, 121-124.
5. Dalla Rosa, I., Camara, Y., Durigon, R., Moss, C.F., Vidoni, S., Akman, G., Hunt, L., Johnson, M.A., Grocott, S., Wang, L., et al. (2016). MPV17 Loss Causes Deoxynucleotide Insufficiency and Slow DNA Replication in Mitochondria. PLoS genetics 12, e1005779.
6. Poulton, J., Sewry, C., Potter, C.G., Bougeron, T., Chretien, D., Wijburg, F.A., Morten, K.J., and Brown, G. (1995). Variation in mitochondrial DNA levels in muscle from normal controls. Is depletion of mtDNA in patients with mitochondrial myopathy a distinct clinical syndrome. Journal of inherited metabolic disease 18, 4-20.
7. Ripolone, M., Ronchi, D., Violano, R., Vallejo, D., Fagiolari, G., Barca, E., Lucchini, V., Colombo, I., Villa, L., Berardinelli, A., et al. (2015). Impaired Muscle Mitochondrial Biogenesis and Myogenesis in Spinal Muscular Atrophy. JAMA neurology 72, 666-675.
8. de Koning, T.J., de Vries, L.S., Groenendaal, F., Ruitenbeek, W., Jansen, G.H., Poll-The, B.T., and Barth, P.G. (1999). Pontocerebellar hypoplasia associated with respiratory-chain defects. Neuropediatrics 30, 93-95.
9. Schirris, T.J., Renkema, G.H., Ritschel, T., Voermans, N.C., Bilos, A., van Engelen, B.G., Brandt, U., Koopman, W.J., Beyrath, J.D., Rodenburg, R.J., et al. (2015). Statin-Induced Myopathy Is Associated with Mitochondrial Complex III Inhibition. Cell Metab 22, 399-407.
10. Rayner, K.J., Suarez, Y., Davalos, A., Parathath, S., Fitzgerald, M.L., Tamehiro, N., Fisher, E.A., Moore, K.J., and Fernandez-Hernando, C. (2010). MiR-33 contributes to the regulation of cholesterol homeostasis. Science 328, 1570-1573.
11. Torres, J.Z., Summers, M.K., Peterson, D., Brauer, M.J., Lee, J., Senese, S., Gholkar, A.A., Lo, Y.C., Lei, X., Jung, K., et al. (2011). The STARD9/Kif16a kinesin associates with mitotic microtubules and regulates spindle pole assembly. Cell 147, 1309-1323.
12. Nangaku, M., Sato-Yoshitake, R., Okada, Y., Noda, Y., Takemura, R., Yamazaki, H., and Hirokawa, N. (1994). KIF1B, a novel microtubule plus end-directed monomeric motor protein for transport of mitochondria. Cell 79, 1209-1220.
13. Mootha, V.K., Lindgren, C.M., Eriksson, K.F., Subramanian, A., Sihag, S., Lehar, J., Puigserver, P., Carlsson, E., Ridderstrale, M., Laurila, E., et al. (2003). PGC-1alpha-responsive genes involved in oxidative phosphorylation are coordinately downregulated in human diabetes. Nat Genet 34, 267-273.
14. Medina-Gomez, G., Gray, S., and Vidal-Puig, A. (2007). Adipogenesis and lipotoxicity: role of peroxisome proliferator-activated receptor gamma (PPARgamma) and PPARgammacoactivator-1 (PGC1). Public health nutrition 10, 1132-1137.

15. Reyes, A., He, J., Mao, C.C., Bailey, L.J., Di Re, M., Sembongi, H., Kazak, L., Dzionek, K., Holmes, J.B., Cluett, T.J., et al. (2011). Actin and myosin contribute to mammalian mitochondrial DNA maintenance. Nucleic acids research 39, 5098-5108.

Web Resources

dbSNP, http://www.ncbi.nlm.nih.gov/SNP/

ExAC http://exac.broadinstitute.org/

GeneGo MetaCore, https://portal.genego.com/

MUSCLE, http://www.ebi.ac.uk/Tools/msa/muscle/

1000 Genomes Project (http://www.1000genomes.org/)https://portal.genego.com/
